# 11th German Conference on Chemoinformatics (GCC 2015): Fulda, Germany. 8–10 November 2015

**DOI:** 10.1186/s13321-016-0119-5

**Published:** 2016-04-26

**Authors:** Uli Fechner, Chris de Graaf, Andrew E. Torda, Stefan Güssregen, Andreas Evers, Hans Matter, Gerhard Hessler, Nicola J. Richmond, Peter Schmidtke, Marwin H. S. Segler, Mark P. Waller, Stefanie Pleik, Joan-Emma Shea, Zachary Levine, Ryan Mullen, Karina van den Broek, Matthias Epple, Hubert Kuhn, Andreas Truszkowski, Achim Zielesny, Johannes (Hans) Fraaije, Ruben Serral Gracia, Stefan M. Kast, Krishna C. Bulusu, Andreas Bender, Abraham Yosipof, Oren Nahum, Hanoch Senderowitz, Timo Krotzky, Robert Schulz, Gerhard Wolber, Stefan Bietz, Matthias Rarey, Markus O. Zimmermann, Andreas Lange, Manuel Ruff, Johannes Heidrich, Ionut Onlia, Thomas E. Exner, Frank M. Boeckler, Marcel Bermudez, Dzmitry S. Firaha, Oldamur Hollóczki, Barbara Kirchner, Christofer S. Tautermann, Andrea Volkamer, Sameh Eid, Samo Turk, Friedrich Rippmann, Simone Fulle, Noureldin Saleh, Giorgio Saladino, Francesco L. Gervasio, Elke Haensele, Lee Banting, David C. Whitley, Jana Sopkova-de Oliveira Santos, Ronan Bureau, Timothy Clark, Achim Sandmann, Harald Lanig, Patrick Kibies, Jochen Heil, Franziska Hoffgaard, Roland Frach, Julian Engel, Steven Smith, Debjit Basu, Daniel Rauh, Oliver Kohlbacher, Frank M. Boeckler, Jonathan W. Essex, Michael S. Bodnarchuk, Gregory A. Ross, Arndt R. Finkelmann, Andreas H. Göller, Gisbert Schneider, Tamara Husch, Christoph Schütter, Andrea Balducci, Martin Korth, Fidele Ntie-Kang, Stefan Günther, Wolfgang Sippl, Luc Meva’a Mbaze, Fidele Ntie-Kang, Conrad V. Simoben, Lydia L. Lifongo, Fidele Ntie-Kang, Philip Judson, Jiří Barilla, Miloš V. Lokajíček, Hana Pisaková, Pavel Simr, Natalia Kireeva, Alexandre Petrov, Denis Ostroumov, Vitaly P. Solovev, Vladislav S. Pervov, Nils-Ole Friedrich, Kai Sommer, Matthias Rarey, Johannes Kirchmair, Eugen Proschak, Julia Weber, Daniel Moser, Lena Kalinowski, Janosch Achenbach, Mark Mackey, Tim Cheeseright, Gerrit Renner, Gerrit Renner, Torsten C. Schmidt, Jürgen Schram, Marion Egelkraut-Holtus, Albert van Oeyen, Tuomo Kalliokoski, Denis Fourches, Akachukwu Ibezim, Chika J. Mbah, Umale M. Adikwu, Ngozi J. Nwodo, Alexander Steudle, Brian B. Masek, Stephan Nagy, David Baker, Fred Soltanshahi, Roman Dorfman, Karen Dubrucq, Hitesh Patel, Oliver Koch, Florian Mrugalla, Stefan M. Kast, Qurrat U. Ain, Julian E. Fuchs, Robert M. Owen, Kiyoyuki Omoto, Rubben Torella, David C. Pryde, Robert Glen, Andreas Bender, Petr Hošek, Vojtěch Spiwok, Lewis H. Mervin, Ian Barrett, Mike Firth, David C. Murray, Lisa McWilliams, Qing Cao, Ola Engkvist, Dawid Warszycki, Marek Śmieja, Andrzej J. Bojarski, Natalia Aniceto, Alex Freitas, Taravat Ghafourian, Guido Herrmann, Valentina Eigner-Pitto, Alexandra Naß, Rafał Kurczab, Andrzej J. Bojarski, Andreas Lange, Marcel B. Günther, Susanne Hennig, Felix M. Büttner, Christoph Schall, Adrian Sievers-Engler, Francesco Ansideri, Pierre Koch, Thilo Stehle, Stefan Laufer, Frank M. Böckler, Barbara Zdrazil, Floriane Montanari, Gerhard F. Ecker, Christoph Grebner, Anders Hogner, Johan Ulander, Karl Edman, Victor Guallar, Christian Tyrchan, Johan Ulander, Christian Tyrchan, Wolfgang Klute, Fredrik Bergström, Christian Kramer, Quoc Dat Nguyen, Roland Frach, Patrick Kibies, Steven Strohfeldt, Saraphina Böttcher, Tim Pongratz, Dominik Horinek, Stefan M. Kast, Bernd Rupp, Raed Al-Yamori, Michael Lisurek, Ronald Kühne, Filipe Furtado, Karina van den Broek, Ludger Wessjohann, Miriam Mathea, Knut Baumann, Siti Zuraidah Mohamad-Zobir, Xianjun Fu, Tai-Ping Fan, Andreas Bender, Maximilian A. Kuhn, Christoph A. Sotriffer, Azedine Zoufir, Xitong Li, Lewis Mervin, Ellen Berg, Mark Polokoff, Wolf D. Ihlenfeldt, Wolf D. Ihlenfeldt, Jette Pretzel, Zayan Alhalabi, Robert Fraczkiewicz, Marvin Waldman, Robert D. Clark, Neem Shaikh, Prabha Garg, Alexander Kos, Hans-Jürgen Himmler, Achim Sandmann, Christophe Jardin, Heinrich Sticht, Thomas B. Steinbrecher, Markus Dahlgren, Daniel Cappel, Teng Lin, Lingle Wang, Goran Krilov, Robert Abel, Richard Friesner, Woody Sherman, Ina A. Pöhner, Joanna Panecka, Rebecca C. Wade, Stefan Bietz, Karen T. Schomburg, Matthias Hilbig, Matthias Rarey, Christian Jäger, Vivien Wieczorek, Lance M. Westerhoff, Oleg Y. Borbulevych, Hans-Ulrich Demuth, Mirko Buchholz, Denis Schmidt, Thomas Rickmeyer, Timo Krotzky, Peter Kolb, Sumit Mittal, Elsa Sánchez-García, Mauro S. Nogueira, Tiago B. Oliveira, Fernando B. da Costa, Thomas J. Schmidt

**Affiliations:** 1 GDCh-CIC Division Associated Board Member, Beilstein-Institut zur Förderung der Chemischen Wissenschaften, Trakehner Str. 7–9, 60487 Frankfurt, Germany; 2 Division Medicinal Chemistry, Amsterdam Institute for Molecules, Medicines and Systems (AIMMS), VU University, Amsterdam, The Netherlands; 3 Centre for Bioinformatics, Uni Hamburg, Bundesstr. 43, 20146, Hamburg Germany; 4 Sanofi-Aventis Deutschland GmbH, 65926 Frankfurt am Main, Germany; 5 GlaxoSmithKline, Stevenage, SG1 2NY UK; 6 Discngine, Paris, 75011 France; 7 Organisch-Chemisches Institut, Westfälische Wilhelms-Universität, Münster, Germany; 8 Bundeskriminalamt Wiesbaden, Central Analytics II, 65173 Wiesbaden, Germany; 9 Department of Chemistry and Biochemistry, University of California, Santa Barbara, CA 93111 USA; 10 Center for Drug Research, Ludwig-Maximilians University Munich, Munich, Germany; 11 Inorganic Chemistry and Center for Nanointegration, University of Duisburg-Essen, Essen, Germany; 12 CAM-D Technologies, Essen, Germany; 13 Institute for Bioinformatics and Chemoinformatics, Westphalian University of Applied Sciences, Recklinghausen, Germany; 14 Leiden University, Leiden, Netherlands; 15 Culgi BV, Berlin, Germany; 16 Physikalische Chemie III, TU Dortmund, 44227 Dortmund, Germany; 17 Centre for Molecular Informatics, Department of Chemistry, University of Cambridge, Lensfield Road, Cambridge, CB2 1EW United Kingdom; 18 Unilever Centre for Molecular Informatics, Department of Chemistry, Lensfield Road, Cambridge, CB2 1EW UK; 19 Centre for Molecular Science Informatics, Department of Chemistry, University of Cambridge, Cambridge, CB2 1EW UK; 20 Department of Chemistry, Bar-Ilan University, Ramat-Gan, 5290002 Israel; 21 Department of Pharmaceutical Chemistry, University of Marburg, Marburg, Germany; 22 Pharmaceutical and Medicinal Chemistry, Institute of Pharmacy, Freie Universität Berlin, Königin-Luise Straße 2+4, 14195 Berlin, Germany; 23 Computer-Aided Drug Design, Institute of Pharmacy, Freie Universität Berlin, 14195 Berlin, Germany; 24 Center for Bioinformatics, University of Hamburg, 20146 Hamburg, Germany; 25 Department of Pharmaceutical and Medicinal Chemistry, Eberhard Karls University Tuebingen, Tuebingen, Germany; 26 Department of Pharmaceutical and Medicinal Chemistry, Institute of Pharmaceutical Sciences, Eberhard Karls University Tübingen, Tübingen, Germany; 27 Center for Bioinformatics Tuebingen (ZBIT), Eberhard Karls University Tuebingen, Tuebingen, Germany; 28 Departement of Pharmaceutical Science, Mol. Design, Eberhard Karls University Tuebingen, Auf der Morgenstelle 8, 72076 Tuebingen, Germany; 29 Mulliken Center for Theoretical Chemistry, University of Bonn, 53115 Bonn, Germany; 30 Boehringer Ingelheim Pharma GmbH & Co. KG, Lead Identification and Optimization Support, Birkendorfer Str. 65, 88397 Biberach a.d. Riss, Germany; 31 BioMed X Innovation Center, Im Neuenheimer Feld 583, 69120 Heidelberg, Germany; 32 Merck KGaA, Merck Serono, Global Computational Chemistry, Frankfurter Str. 250, 64293 Darmstadt, Germany; 33 Computer-Chemie-Centrum and Interdisciplinary Center for Molecular Materials Friedrich-Alexander-Universität Erlangen-Nürnberg, Nägelsbachstraße 25, 91052 Erlangen, Germany; 34 Department of Chemistry and Institute of Structural and Molecular Biology, University College London, London, WC1E 6BT United Kingdom; 35 Centre for Molecular Design, School of Pharmacy and Biomedical Sciences, University of Portsmouth, St Michael’s Building, White Swan Road, Portsmouth, PO1 2DT United Kingdom; 36 Centre d’Etudes et de Recherche sur le Médicament de Normandie, UPRES EA 4258 - FR CNRS 3038 INC3M, Boulevard Becquerel, 14032 CAEN Cedex, France; 37 Department of Chemistry and Pharmacy, Computer Chemistry Center, FAU Erlangen-Nürnberg, Naegelsbachstr. 25, 91052 Erlangen, Germany; 38 Bioinformatics, Institute for Biochemistry, FAU Erlangen-Nürnberg, Fahrstr. 17, 91054 Erlangen, Germany; 39 Chemische Biologie, TU Dortmund, 44227 Dortmund, Germany; 40 School of Chemistry, University of Southampton, Southampton, SO17 1BJ UK; 41 Swiss Federal Institute of Technology (ETH), Institute of Pharmaceutical Sciences, 8093 Zürich, Switzerland; 42 Institute for Theoretical Chemistry, Ulm University, 89081 Ulm, Germany; 43 Helmholtz Institute Ulm, Karlsruhe Institute of Technology, 89081 Ulm, Germany; 44 Department of Chemistry, University of Buea, Buea, South West Region Cameroon; 45 Institut für Pharmazie, Martin-Luther University of Halle-Wittenberg, Halle, 06120 Germany; 46 Institut für Pharmazeutische Wissenschaften, Universität Freiburg, 79104 Freiburg, Germany; 47 Institute of Pharmacy, University of Halle, 06120 Halle (Saale), Germany; 48 Institute of Pharmacy, Martin-Luther University Halle-Wittenberg, Halle-Wittenberg, Germany; 49 Department of Chemistry, University of Douala, Douala, Littoral Region Cameroon; 50 Department of Chemistry, Chemical and Bioactivity Information Centre, University of Buea, Buea, South West Region Cameroon; 51 Chemical and Bioactivity Information Centre, Department of Chemistry, University of Buea, Buea, South West Region Cameroon; 52 Chemical Bioactivity Information Centre, Heather Lea, Bland Hill, Norwood, Harrogate, HG3 1TE UK; 53 Faculty of Science, J. E. Purkinje University in Usti nad Labem, Ústí nad Labem, 400 96 Czech Republic; 54 Institute of Physics, Academy of Sciences of the Czech Republic, Praha, 182 21 Czech Republic; 55 Frumkin Institute of Physical Chemistry and Electrochemistry RAS, Moscow, 119071 Russia; 56 Moscow Institute of Physics and Technology, Dolgoprudny, Russia, 141700 Russia; 57 Kurnakov Institute of General and Inorganic Chemistry, Moscow, 119071 Russia; 58 University of Hamburg, Center for Bioinformatics, Hamburg, 20146 Germany; 59 Institute of Pharmaceutical Chemistry, Goethe University, Frankfurt, 60438 Germany; 60 Cresset, Litlington, Cambridgeshire, SG8 0SS UK; 61 Faculty of Chemistry, University of Applied Sciences Niederrhein, Krefeld, 47798 Germany; 62 Instrumental Analytical Chemistry, University of Duisburg-Essen, Essen, 45141 Germany; 63 Shimadzu Europa GmbH, Duisburg, 47269 Germany; 64 CARAT GmbH, Bocholt, 46395 Germany; 65 Lead Discovery Center GmbH, Otto-Hahn-Straße 15, 44227 Dortmund, Germany; 66 Department of Chemistry, Bioinformatics Research Center, North Carolina State University, Raleigh, NC 27695 USA; 67 Department of Pharmaceutical and Medicinal Chemistry, Faculty of Pharmaceutical Science, University of Nigeria, Nsukka, 410001 Nigeria; 68 Department of Pharmaceutics, Faculty of Pharmaceutical Science, University of Nigeria, Nsukka, 410001 Nigeria; 69 Certara International, Martin-Kollar-Straße 17, 81829 München, Germany; 70 Certara, St Louis, MO 63101 USA; 71 Department of Chemistry and Chemical Biology, TU Dortmund, Dortmund, Germany; 72 Department of Chemistry and Chemical Biology, TU Dortmund University, Otto-Hahn-Str. 6, 44227 Dortmund, Germany; 73 Worldwide Medicinal Chemistry, Pfizer Neusentis, The Portway Building, Granta Park, Great Abington, Cambridge, Cb21 6GS United Kingdom; 74 Department of Biochemistry, University of Chemistry and Technology, Prague, Technická 3, Prague 6, 166 28 Czech Republic; 75 Discovery Sciences, AstraZeneca R&D Cambridge, Cambridge Science Park, UK; 76 Discovery Sciences, AstraZeneca R&D Alderley Park, Alderley Park, UK; 77 Discovery Sciences, AstraZeneca R&D, Boston, MA USA; 78 Chemistry Innovation Centre, AstraZeneca R&D, Mölndal, Sweden; 79 Institute of Pharmacology Polish Academy of Sciences, Krakow, 31-343 Poland; 80 Faculty of Mathematics and Computer Science Jagiellonian University, Krakow, 30-348 Poland; 81 Medway School of Pharmacy, Universities of Kent and Greenwich, Kent, ME4 4TB UK; 82 School of Computing, University of Kent, Canterbury, Kent CT2 7NF UK; 83 Georg Thieme Verlag KG, Stuttgart, 70469 Germany; 84 InfoChem GmbH, München, 81241 Germany; 85 Institut für Pharmazie, Freie Universität Berlin, 14195 Berlin, Deutschland; 86 Department of Medicinal Chemistry, Institute of Pharmacology Polish Academy of Sciences, 12 Smetna Street, 31-343 Cracow, Poland; 87 Departement of Pharmaceutical Science, Medicinal Chemistry, Eberhard Karls University Tuebingen, Auf der Morgenstelle 8, 72076 Tuebingen, Germany; 88 Interfaculty Institute of Biochemistry, Eberhard Karls University Tuebingen, Hoppe-Seyler-Str. 4, 72076 Tuebingen, Germany; 89 Departement of Pharmaceutical Analysis and Bioanalysis, Eberhard Karls University Tuebingen, Auf der Morgenstelle 8, 72076 Tuebingen, Germany; 90 Department of Pharmaceutical Chemistry, Division of Drug Design and Medicinal Chemistry, Pharmacoinformatics Research Group, University of Vienna, Althanstraße 14, 1090 Vienna, Austria; 91 CVMD, AstraZeneca, Mölndal, Sweden; 92 Discovery Sciences, AstraZeneca, Mölndal, Sweden; 93 Joint BSC-IRB Research Program in Computational Biology, BSC, Barcelona, Spain; 94 Institució Catalana de Recerca i Estudis Avançats (ICREA), Barcelona, Spain; 95 RIA, AstraZeneca, Mölndal, Sweden; 96 AstraZeneca, CVMD iMED, Mölndal, Sweden; 97 RIA iMED, Mölndal, Sweden; 98 RDI, Mölndal, Sweden; 99 F. Hoffmann-La Roche, Pharma Early Research and Development, Basel, Switzerland; 100 Institut für Physikalische und Theoretische Chemie, Universität Regensburg, 93040 Regensburg, Germany; 101 Structural Biology, AG Computational Chemistry/Drug Design, Leibniz-Institut für Molekulare Pharmakologie (FMP), 13125 Berlin, Germany; 102 Department of Bioorganic Chemistry, Leibniz-Institute of Plant Biochemistry, Weinberg 3, 06120 Halle (Saale), Germany; 103 Chemistry Department, Ludwig-Maximilians-Universität München, Butenandtstr. 7, 81377 Munich, Germany; 104 Institute of Medicinal and Pharmaceutical Chemistry, Braunschweig University of Technology, Braunschweig, Germany; 105 School of Information Management, Shandong University of Traditional Chinese Medicine, 250355 Jinan, China; 106 Department of Pharmacology, University of Cambridge, Tennis Court Road, Cambridge, CB2 1PD United Kingdom; 107 Institute of Pharmacy and Food Chemistry, University of Würzburg, 97074 Würzburg, Germany; 108 BioSeek, Inc., 310 Utah 100, South San Francisco, CA 94080 USA; 109 Xemistry GmbH, 61462 Königstein, Germany; 110 Simulations Plus, Inc., Lancaster, CA 93534 USA; 111 Department of Pharmacoinformatics, National Institute of Pharmaceutical Education and Research (NIPER), Sector-67, S.A.S. Nagar, Punjab, 160 062 India; 112 AKos GmbH, Steinen, Germany; 113 Schrödinger GmbH, Dynamostr. 13, 68165 Mannheim, Germany; 114 Schrödinger Inc., 120 West 45th Street, 17th Floor, New York, NY 10036 USA; 115 Department of Chemistry, Columbia University, 3000 Broadway, New York, NY 10027 USA; 116 Molecular and Cellular Modeling Group, Heidelberg Institute for Theoretical Studies (HITS) gGmbH, Heidelberg, Germany; 117 ZMBH-DKFZ Alliance, Center for Molecular Biology, Heidelberg University, Heidelberg, Germany; 118 Center for Bioinformatics, University of Hamburg, Hamburg, Germany; 119 Fraunhofer Institute for Cell Therapy and Immunology, Department of Drug Design and Target Validation (IZI-MWT), 06120 Halle (Saale), Germany; 120 QuantumBio Inc, 2790 West College Avenue, Suite 900, State College, PA 16801 USA; 121 Pharmaceutical Chemistry, Philipps-University, Marburg, Germany; 122 The Cambridge Crystallographic Data Centre, Cambridge, UK; 123 Max-Planck-Institut für Kohlenforschung, Kaiser-Wilhelm-Platz 1, 45470 Mülheim an der Ruhr, Germany; 124 Institute of Pharmaceutical Biology and Phytochemistry, University of Muenster, Correnstraße 48, 48149 Muenster, Germany; 125 University of São Paulo, Av. do Café S/N, Ribeirão Preto, Brazil; 126 Central Institute for Scientific Computing (ZISC), FAU-Erlangen-Nürnberg, Martensstr. 5a, 91058 Erlangen, Germany; 127 School of Mechanical Engineering, Imperial College London, London, SW1 2AZ UK; 128 Bayer Pharma AG, Global Drug Discovery, 42096 Wuppertal, Germany

## I1 11th German Conference on Chemoinformatics

### Uli Fechner

#### GDCh-CIC Division Associated Board Member, Beilstein-Institut zur Förderung der Chemischen Wissenschaften, Trakehner Str. 7–9, 60487 Frankfurt, Germany

##### **Correspondence:** Uli Fechner - ufechner@beilstein-institut.de

*Journal of Cheminformatics* 2016, **8(Suppl 1)**:I1

 The Chemistry-Information-Computer (CIC) division [1] of the German Chemical Society (Gesellschaft Deutscher Chemiker e.V.) hosted the 11th German Conference on Chemoinformatics (GCC2015) from the 8th to the 10th of November 2015 in Fulda, Germany [2]. The conference reflected the new role of chemoinformatics in the modern information world. Discussed topics were related to the utilization of computers in chemistry, pharmacy, material sciences and biology. The Program Committee took great care to compile a scientific program which covers a wide range of subjects: from chemo- and bioinformatics to molecular modelling, from fundamental academic research to industrial applications.

**Fig. 1:**
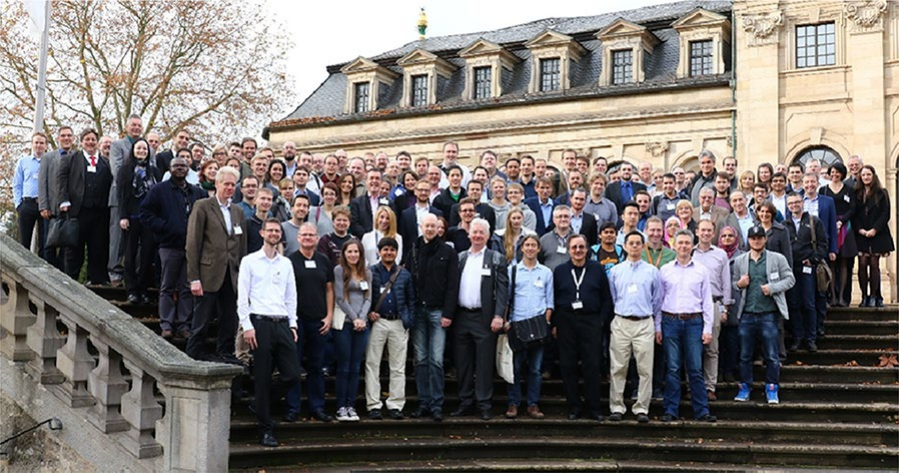
Participants of the 11th German Conference on Chemoinformatics (GCC2015), November 8–10, 2015 in Fulda, Germany

The plenary sessions, which comprised a total of 28 oral presentations, started off with a newly introduced session *Sunday Highlights* characterized by three keynotes from high-profile speakers. Subsequent sessions were a mix of invited keynote speakers and selected contributions submitted by the community. The session *Research Telegrams* was exclusively dedicated to the presentation of the current status of PhD students’ work. In addition, 61 poster contributions were presented in two poster sessions. The more than 135 attendees from 12 nations proofed that the German Conference on Chemoinformatics is a well-established event in the international Chemoinformatics and Modelling community (Fig. 1).

**Fig. 2:**
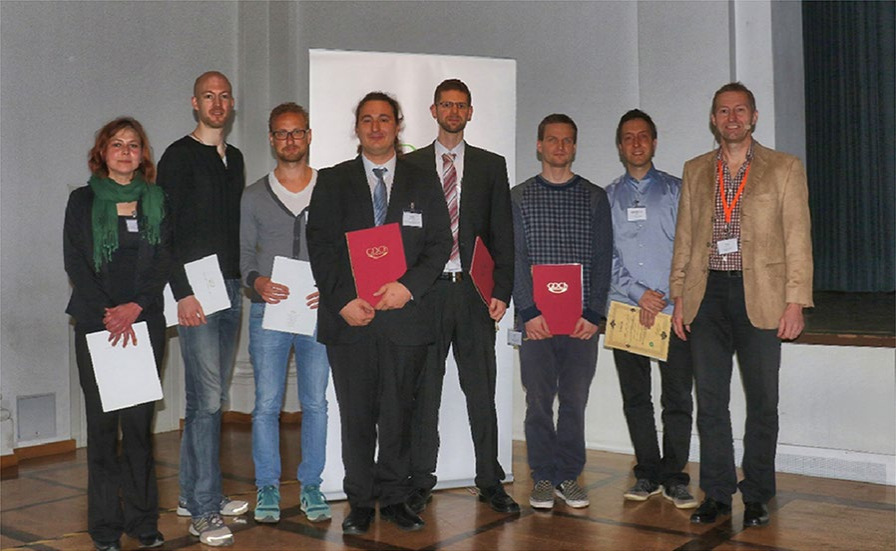
The awardees of the CIC Poster Award 2015 and the CIC-Award for Computational Chemistry 2014 and 2015: from left to right, Christiane Ehrt (poster award, TU Dortmund University, Germany), Tobias Brinkjost (poster award, TU Dortmund, Germany), Stefan Bietz (poster award, Universität Hamburg, Germany), Patrick J. Kibies (CIC-Award for Computational Chemistry, master thesis, TU Dortmund, Germany), Dr. Achim Sandmann (CIC-Award for Computational Chemistry, dissertation thesis, Friedrich-Alexander-Universität Erlangen-Nürnberg, Germany), Manuel Ruff (CIC-Award for Computational Chemistry, master thesis, Eberhard Karls University Tübingen, Germany), Markus O. Zimmermann (CIC-Award for Computational Chemistry, dissertation thesis, Eberhard Karls University Tübingen, Germany) and Thomas Engel (Chair of the GDCh CIC division)

The CIC-Award for Computational Chemistry honors outstanding doctoral dissertations and master thesis in the fields of the scientific specialties of the Chemistry-Information-Computer division. In 2014, the German Conference on Chemoinformatics was jointly held with the International Conference on Chemical Structures (ICCS) [3], which led to the awarding of the CIC-Award for Computational Chemistry for both the year 2014 and the year 2015 at the GCC2015. The CIC-Award for Computational Chemistry for the best dissertation thesis for 2014 was granted to Achim Sandmann, who worked on his dissertation thesis under the supervision of Dr. Harald Lahnig at the Friedrich-Alexander-Universität Erlangen-Nürnberg. Both Patrick Kibies, supervised by Prof. Dr. Stefan Kast from the TU Dortmund, and Manuel Ruff, supervised by Prof. Dr. Frank Böckler from the Eberhard Karls University Tübingen, were granted the CIC-Award for Computational Chemistry for their outstanding master theses. Among the six oral contributions presented in the plenary session *Research Telegrams* the work of Markus Zimmermann titled “ChemPLPXB: Implementation of QM-based terms for the recognition of halogen bonding in drug design” was selected as the best dissertation thesis 2015. Markus Zimmermann prepared his dissertation thesis under the supervision of Prof. Dr. Frank Böckler at the Eberhard Karls University Tübingen (Fig. 2).

**References**

1. GDCh CIC Division, http://www.gdch.de/cic. Accessed 11 Nov 2015.

2. German Conference on Cheminformatics 2015, http://www.gdch.de/gcc2015. Accessed 11. Nov 2015.

3. Wagener M, Oellien F, Fechner U, Rarey M. 10th ICCS/GCC conference: 40 years of cheminformatics. J Chem Inf Model 2015;55:1087–1087.

## O1 3D-e-Chem: structure-based navigation of medicinal chemistry space

### Chris de Graaf

#### Division Medicinal Chemistry, Amsterdam Institute for Molecules, Medicines and Systems (AIMMS), VU University Amsterdam, Netherlands

##### **Correspondence:** Chris de Graaf - c.de.graaf@vu.nl

*Journal of Cheminformatics* 2016, **8(Suppl 1)**:O1

This presentation will describe how structural biology, molecular pharmacology, and medicinal chemistry studies can be combined with molecular modeling and chemoinformatics analyses for a more accurate description and prediction of structural determinants of protein-ligand binding, functional activity, and selectivity. The challenges and possibilities of structural chemogenomics studies will be discussed, including the integration of large volumes of heterogeneous pharmacological and chemical data for different protein targets and the development of structure-based virtual screening and computer-aided drug design approaches to discover novel small molecule ligands with well defined functional activity and protein selectivity profiles. The potential of molecular dynamics simulation methods to complement hybrid structural biology studies will be demonstrated for the investigation the mechanisms of conformational selection and protein-ligand binding kinetics. In the final part of the presentation structural protein-ligand interaction databases will be described that link structure-based protein-ligand interaction maps to protein ligand topology and can be used as structural chemogenomics tools to navigate medicinal chemistry space.

**Acknowledgments:** The Netherlands eScience Center (NLeSC)/Netherlands Organisation for Scientific Research (NWO) (Enabling Technologies project: 3D-e-Chem) [027.014.201], EU/EFPIA Innovative Medicines Initiative Joint Undertaking, K4DD grant no 115366, COST Action CM1207 (GLISTEN).

## O2 Why I do not like Monte Carlo

### Andrew E. Torda

#### Centre for Bioinformatics, Uni Hamburg, Bundesstr. 43, 20146 Hamburg, Germany

##### **Correspondence:** Andrew E. Torda - torda@zbh.uni-hamburg.de

*Journal of Cheminformatics* 2016, **8(Suppl 1)**:O2

Children who misbehave are often punished by being made to implement simulated annealing on top of a Monte Carlo scheme. Students who cannot afford a Gucci handbag strive to be fashionable by programming up a genetic algorithm. Computer scientists often take problems from chemistry, beat them until their bones have turned to jelly and squash them into a form suitable for a dynamic programming method.

The question is, whether these approaches are really the best for problems like docking or RNA folding, where you may want optimal solutions or a sampling of likely solutions. If your problem has gradient information or a well-defined statistical distribution, maybe you should use them with pride in your voice and a bounce in your step.

## O3 Predictive modelling for biologics: chemoinformatics meets bioinformatics

### Stefan Güssregen, Andreas Evers, Hans Matter, Gerhard Hessler

#### Sanofi-Aventis Deutschland GmbH, Frankfurt am Main, 65926, Germany

##### **Correspondence:** Stefan Güssregen - stefan.guessregen@sanofi.com

*Journal of Cheminformatics* 2016, **8(Suppl 1)**:O3

More than 20 years after their first climax in the 80s and 90s, therapeutic peptides and proteins are currently experiencing a renaissance as drug molecules as it is believed that they offer key advantages over traditional small molecules, such as fewer toxic side effects. Identification of compounds for clinical development still requires multiparameter optimization as these modalities may suffer from potential liabilities such as chemical stability, solubility and aggregation propensity.

In this talk some of the work in the area of structure-activity and structure-property modelling and its application in the design of new peptides and proteins will be highlighted. Traditionally, many computational prediction tools that have been reported in the literature are purely based on sequence-based descriptors. Our strategy bridges the worlds of bioinformatics and chemoinformatics by using approaches that are based on 3D structures of individual amino acids as well as on the full 3D structure of peptides or proteins.

Here, the applicability of such methods to unnatural or modified amino acids is of particular importance as introducing those into the peptide sequence might significantly impact biophysical peptide properties. While the Z-scale descriptors introduced originally by Wold [1] are based on experimental values, we have applied computational amino acid descriptors [2] derived using the CoMFA method [3] for building QSAR models, which are amenable to general amino acids. Details will be shown on how the descriptors are derived as well as approaches for the analysis and interpretation of the resulting models.

**References**

1. Hellberg S, Sjoestroem M, Skagerberg B, Wold S. Peptide Quantitative Structure-activity relationships, a multivariate approach. J Med Chem 1987;30:1126–35.

2. Matter H. A validation study of molecular descriptors for the rational design of peptide libraries. J Pept Res 1998;52:305–14.

3. Cramer RD, Patterson DE, Bunce JD. Comparative molecular field analysis (CoMFA). 1. Effect of shape on binding of steroids to carrier proteins. J Am Chem Soc 1988;110:5959–67.

## O4 Target deconvolution of phenotypic screens using Formal Concept Analysis

### Nicola J. Richmond^1^, Peter Schmidtke^2^

#### ^1^GlaxoSmithKline, Stevenage, SG1 2NY, UK; ^2^Discngine, Paris, 75011, France

##### **Correspondence:** Nicola J. Richmond - nicola.j.richmond@gsk.com

*Journal of Cheminformatics* 2016, **8(Suppl 1)**:O4

With the advent of the Human Genome Project came the industrialisation of the drug discovery process and a belief that combinatorial chemistry and high throughput screening would deliver molecules with increased potency, against a single target of interest. Yet the attrition rate is still at the 10% mark and there remain many human diseases for which no effective treatment exists. As Swinney et al have shown [1], there is compelling evidence that first-in-class drugs are more likely to be found by assays that measure a clinically meaningful phenotype in a physiologically relevant system rather than a single target based screening approach in an artificial setting.

One perceived issue with phenotypic screening is the lack of mechanistic knowledge. Whilst understanding mechanism of action (MOA) is not a prerequisite for FDA approval, it can guide a medicinal chemistry effort, predict potential toxicities and help define patient populations for clinical trials and ultimately the market place. There are a number of *in vitro* approaches to target deconvolution. However, these tend to be of lower throughput and better placed later in a screening cascade. So there is a real need for *in silico* based approaches that can be deployed early on in a drug discovery programme to identify potential MOAs.

Using publicly available data on the Published Kinase Inhibitor Set (PKIS) [2,3], we describe the application of Formal Concept Analysis (FCA), an association mining technique with roots in set theory, to the problem of deconvoluting a phenotypic screen. We describe each compound in the PKIS by the set of kinases it inhibits. We then construct a Galois Lattice, whose nodes correspond to a set of compounds inhibiting a common set of kinases and where two nodes are connected if the compound set of the child node is a subset of the compound set of the parent node. Lattice nodes enriched with compounds that promote neurite outgrowth in rat inform on which kinases should be targeted when seeking small molecules that encourage CNS axon repair following injury. The targets we identify using this unsupervised and interpretable approach, are in line with those identified in [3] where here the authors use a combination of support vector machines, considered a black box method, and mutual information, then confirm in siRNA studies.

**References**

1. Swinney DC, Anthony J. How were new medicines discovered? Nat Rev Drug Discov. 2011;10:507–19.

2. Drewry DH, Willson TM, Zuercher WJ. Seeding collaborations to advance kinase science with the GSK Published Kinase Inhibitor Set (PKIS). Curr Top Med Chem 2014;14:340–2.

3. Al-Ali H, Lee DH, Danzi MC, Nassif H, Gautam P, Wennerberg K, Zuercher WJ, Drewry DH, Lee JK, Lemmon VP, Bixby JL. Rational polypharmacology: systematically identifying and engaging multiple drug targets to promote axon growth. ACS Chem Biol 2015;10:1939–51.

## O5 Discovering unprecedented catalytic reactions by chemical machine reasoning

### Marwin H. S. Segler, Mark P. Waller

#### Organisch-Chemisches Institut, Westfälische Wilhelms-Universität Münster, Germany

##### **Correspondence:** Marwin H. S. Segler - marwin.segler@wwu.de

*Journal of Cheminformatics* 2016, **8(Suppl 1)**:O5

Chemists pursue the discovery of new chemical reactions to meet the demand for transformations that are more efficient, are more environmentally benign or allow the synthesis of previously inaccessible molecules [1]. Usually, discovery starts with an idea derived from knowledge, which is subsequently tested in experiments. The outcome of the experiments then increases knowledge, leading to a cyclic process. In Cognitive Science, initial hypothesis generation is attributed to analogical reasoning, and creativity is described as a process of combining seemingly unrelated knowledge [2]. However, as published chemical knowledge increases rapidly, many interesting hypotheses may never be recognized. Computational approaches for the discovery of unprecedented reactions are therefore desirable, but only seldom reported and not applicable to modern catalytic reactions [3,4]. We have developed a scalable model to obtain hypotheses for unprecedented catalytic chemical reactions based on a set of known reactions. Given just the starting materials as the input, our model proposes hypotheses about the product and the reaction conditions by identifying analogies in the reactivity the reactants. In a validation study, the model correctly assigns 94% of a reaction set unknown to the model as plausible reactions and provides the correct product structures. In further studies, we show that our model can infer correct products and accurate (or highly similar and reasonable) reaction conditions incl. catalysts and reagents for recently published reactions.

**References**

1. Collins KD, Gensch T, Glorius F. Contemporary screening approaches to reaction discovery and development. Nature Chem. 2014;6:859–71.

2. Klahr D. Exploring science: the cognition and development of discovery processes. Boston, CA: MIT Press; 2002.

3. Ugi I, Bauer J, Bley K, Dengler A, Dietz A, Fontain E, Gruber B, Herges R, Knauer M, Reitsam K. Computer-assisted solution of chemical problems—the historical development and the present state of the art of a new discipline of chemistry. Angew Chem Int Ed Engl 1993;32:201–27.

4. Herges R, Hoock C. Reaction planning: computer-aided discovery of a novel elimination reaction. Science 1992;255:711–13.

## O6 Fight against growing crime: Forensic science research

### Stefanie Pleik

#### Bundeskriminalamt Wiesbaden, Central Analytics II, 65173 Wiesbaden, Germany

##### **Correspondence:** Stefanie Pleik - stefanie.pleik@bka.bund.de

*Journal of Cheminformatics* 2016, **8(Suppl 1)**:O6

Nowadays crime scene investigations and evidence analysis are enormously challenged by the rapidly growing crime. In the last decade crime situation has changed dramatically and forensic science must be fitted to current challenges (e.g. cybercrime) at its best. Keeping the forensics up to date, it is essential to perform research projects for the development of new and powerful methods. Over the course of time analytical achievements provide additional possibilities for case work analysis we could not imagine a couple of years earlier. Forensic science must benefit from these efforts in the field of analytics. It needs to be proved frequently, how new analytical methods can support forensic investigations. So far unsolved problems can be faced by introducing new techniques in the forensics considering the applicability in case work. It is an important point to always think about how feasible certain methods are for case work. Besides the development of new techniques, established methods need to be optimized frequently to always obtain most reliable results for crime scene investigations. Due to mentioned reasons, research and development in general is a big issue in forensics and especially for the forensic science institute of the Bundeskriminalamt Germany. Some selected research projects will be presented to give an overview of potential forensic methods for the future.

## O7 Effect of surfaces in modulating protein folding and aggregation mechanisms

### Joan-Emma Shea, Zachary Levine, Ryan Mullen

#### Department of Chemistry and Biochemistry, University of California, Santa Barbara, CA, 93111, USA

##### **Correspondence:** Joan-Emma Shea - shea@chem.ucsb.edu

*Journal of Cheminformatics* 2016, **8(Suppl 1)**:O7

Protein-surface interactions are ubiquitous in the crowded cytosol, where proteins encounter a variety of surfaces, ranging from membranes surfaces, to the surfaces presented by chaperone molecules. Protein-surface interactions are also at the heart of a number of emerging technologies, including protein micro-arrays, biosensors and biomaterials. The effect of surfaces on protein structure and stability can vary substantially depending on the chemical composition of the surface. In this talk, I will present coarse-grained as well as detailed atomistic simulations of the folding of small proteins in the presence of surfaces of relevance to biology and biotechnology. Examples will range from adhesion of intrinsically disordered marine mussel proteins on organic thin films, to globular protein adsorption on membrane-mimics.

## O8 Mesoscopic simulation of biomolecular systems

### Karina van den Broek^1^, Matthias Epple^2^, Hubert Kuhn^3^, Andreas Truszkowski^2,4^, Achim Zielesny^4^

#### ^1^Center for Drug Research, Ludwig-Maximilians University Munich, Munich, Germany; ^2^Inorganic Chemistry and Center for Nanointegration, University of Duisburg-Essen, Essen, Germany; ^3^CAM-D Technologies, Essen, Germany; ^4^Institute for Bioinformatics and Chemoinformatics, Westphalian University of Applied Sciences, Recklinghausen, Germany

##### **Correspondence:** Achim Zielesny - achim.zielesny@w-hs.de

*Journal of Cheminformatics* 2016, **8(Suppl 1)**:O8

A basic challenge for biomolecular simulations is the necessity to study comparatively large and complex structures in the order of at least tens of nanometers for comparatively long times up to the microseconds scale. A mesoscopic coarse-grained approach neglects precise atomic interactions but tries to keep essential features of the complex system of interest—and thus may be helpful to adequately describe average collaborative interactions within large biomolecular ensembles and their effects.

The new mesoscopic approach comprises the Molecular Fragment Dynamics (MFD) variant of the established mesoscopic Dissipative Particle Dynamics (DPD) simulation technique, adequate fragmentation schemata for all biomolecular species of interest (e.g., phospholipids, amino acids, peptides and proteins), the construction of a versatile biomolecular fragment set, Cheminformatics concepts and tools for fragment structure representations and handling, concepts for peptide and protein 3D structure treatment like backbone potentials for structural rigidity/flexibility and an integrative software architecture for practical realization [1]. First simulation results for phospholipid membranes, peptides/proteins and their mutual interactions demonstrate opportunities and limitations [2]. 

**References**

1. Truszkowski A, Daniel M, Kuhn H, Neumann S, Steinbeck C, Zielesny A, Epple M. A molecular fragment cheminformatics roadmap for mesoscopic simulation. J Cheminf 2014;6:45.

2. Truszkowski A, van den Broek K, Kuhn H, Zielesny A, Epple M: Mesoscopic simulation of phospholipid membranes, peptides and proteins with molecular fragment dynamics. J Chem Inf Model. 2015;55:983–97.

## O9 Automated mesoscale approach to biomolecular affinity calculations

### Johannes (Hans) Fraaije^1^, Ruben Serral Gracia^2^

#### ^1^Leiden University, Netherlands, ^2^Culgi BV, Berlin

##### **Correspondence:** Johannes (Hans) Fraaije - j.fraaije@chem.leidenuniv.nl

*Journal of Cheminformatics* 2016, **8(Suppl 1)**:O9

We investigate the methodological development of coarse grained ‘mesoscale’ simulations for biomolecular affinity calculations such as in ligand-protein interactions, where the ligand is potentially of macromolecular dimension. The ambitious aim is to reach thermodynamic accuracy of 1–3 kT in free energy of binding prediction, by including all relevant interactions, using soft potentials, thermodynamic integration and calibration using engineering thermodynamics data. In the approach, small groups of 2–3 heteroatoms are combined in a single simulation unit. Such bead, typical of the mesoscale nomenclature, is to be considered a tiny molecular fragment. Since in drug discovery screening of many thousands if not millions of compounds is the rule, the all-determining factor for the success of the approach is the fast automation of fragmentation and ensuing parameterization (Automated Fragmentation Parameterization, AFP). We have developed an AFP protocol that combines elements from chemical informatics (for finding optimal fragments) and engineering thermodynamics (for parameterization through surface charge interactions). The first results look promising. We have calculated fragments for ~100k molecules (PUBCHEM data base) by rule based simulated annealing. Classification of fragments shows a distribution with a typical Zipf regression. We estimate that five to ten thousand unique fragments are enough to cover all organic molecules in the CAS database. As for the parameterization: we rely heavily on non-biological engineering thermodynamics data, such as Vapor-Liquid Equlibrium diagrams from the NIST database (Thermodynamics Data Engine). This unique set of data that covers excess Gibbs energies for a wide range of binary fluid systems, and is an ultimate test for any simulation that aims to produce results of thermodynamic accuracy. So far, this data has been the bread and butter of ultrafast and accurate equation based engineering thermodynamics models, such as UNIFAC or COSMO-RS. Simulation methods up till now have been to slow and inaccurate for massive validation. Here our mesoscale simulation approach reaches an accuracy of a fraction of a kT per fragment (1–3 kT per molecule), this is on par, or better than the engineering methods, with the enormous advantage that our approach is intrinsically 3-dimensional. All is still within very reasonable calculation times on modest computational resources. We complement the presentation with results on logP prediction and lignine, keratine and kinase modelling. The software development is sponsored by CULGI, a large consortium from oil, personal care and chemical industries.

## O10 Computational solvation modeling: from simple liquids to biomolecules

### Stefan M. Kast

#### Physikalische Chemie III, TU Dortmund, 44227 Dortmund, Germany

##### **Correspondence:** Stefan M. Kast - stefan.kast@tu-dortmund.de

*Journal of Cheminformatics* 2016, **8(Suppl 1)**:O10

Solvation plays an essential role for all kinds of physiologically relevant systems and phenomena. Not only water as the key solvent for life but also electrolytes and osmo-lytes modulate molecular interactions with implications for structure, dynamics, and thermodynamics. Computational modeling of (bio-)molecular systems in solution re-quires taking into account the much larger number of solvent particles in comparison with solute species. This results in vast technical effort, e.g., to treat molecular dynam-ics by atomistic simulations in full detail although most of the explicitly considered sol-vent molecules have only statistical influence on biological function due to the sepa-ration of dynamical time scales of solute and solvent.

An efficient way to account for solvent-mediated effects in atomistic simulations is to treat the solvent statistically, either by structureless dielectric continuum models or by computing solvent distribution functions around solute systems. In this talk, the basic concepts of solvation modeling frameworks will be presented and illustrated. After de-veloping the hierarchy of models, particular attention will be paid to analytical theories based on the statistical-mechanical integral equation formalism. While originally de-veloped for simple liquids these methods have been adapted over the years to be ap-plicable to complex biomolecular scenarios [1,2]. Due to the massive reduction of the number of degrees of freedom compared to atomistic simulations, such a framework can also be coupled to polarizable potential models as well as to a quantum-mechanical description of solute-solvent interactions, thus allowing for enhanced pre-dictive capabilities [3,4]. Several example applications will be presented, including hy-dration patterns around biomolecules, chemistry under extreme thermodynamic condi-tions, and complicated problems of coupled protomeric, tautomeric [5] and conforma-tional equilibria of drug-like molecules in solution.

**References**

1. Kast SM, Heil J, Hoffgaard F: Integral equation theory as a solvation model for classical and quantum solute systems. In Sutmann G, Grotendorst J, Gompper G, Marx D, editors. Computational trends in solvation and transport in liquids. Jülich: IAS Series vol 28; 2015. p. 419–34.

2. Kast SM, et al.: A minimalist model for ion partitioning and competition in a K+ channel selectivity filter. J Gen Physiol 2011;138:371–3.

3. Kloss T, Heil J, Kast SM. Quantum chemistry in solution by combining 3D integral equation theory with a cluster embedding approach. J. Phys. Chem. B 2008;112:4337–43.

4. Frach R, Kast SM. Solvation effects on chemical shifts by embedded cluster integral equation theory. J Phys Chem A 2014;118:11620–8.

5. Kast SM, et al. Prediction of tautomer ratios by embedded cluster integral equation theory. J Comput-Aided Mol Des. 2010;24:343–53.

## O11 An integrated chemical, biological and pathological in silico approach towards identifying potent drug combinations

### Krishna C. Bulusu, Andreas Bender

#### Centre for Molecular Informatics, Department of Chemistry, University of Cambridge, UK, CB2 1EW

##### **Correspondence:** Krishna C. Bulusu - kcb27@cam.ac.uk

*Journal of Cheminformatics* 2016, **8(Suppl 1)**:O11

Recent technological advancements in the field of health science brought with it a deluge of data that is waiting to be interpreted. It is critical to understand the information that can be derived from this wealth of data to not just identify the chemical, biological and/or genetic markers/patterns of a particular disease but also to utilize this knowledge to design more potent and selective medicines. However, partly driven by the urgency of this need, we are generating more data than we are able to analyze and interpret.

Most diseases display a ‘pathological footprint’ on a systems biology level, even if the observed phenotype is localized to a tissue or organ. In order to understand and explain such activity, integration of biological and chemical information such as chemical fingerprints with bioactivity profiles, gene expression patterns, pathway annotations, protein interaction networks etc., is vital. This study focuses on integrating experimentally derived chemical, biological and pathological data to identify and explain compound mode-of-action and its impact on disease regulation. Such analysis will not only help define a therapeutic approach towards more efficient and less promiscuous drugs, but also expedite this process by identifying current approved drugs for repurposing that could potentially reverse disease signatures. Drug combinations, which has been shown previously to be a strong approach towards tackling the issues of drug resistance and specificity, will be the primary focus of this study.

This talk will highlight in silico approaches towards next-generation drug discovery along with relevant resources and datasets that are/can be integrated to achieve a ‘systems biology’ picture of disease progression and regulation.

## O12 Statistical modeling in material sciences

### Abraham Yosipof, Oren Nahum, Hanoch Senderowitz

#### Department of Chemistry, Bar-Ilan University, Ramat-Gan, 5290002, Israel

##### **Correspondence:** Hanoch Senderowitz - hsenderowitz@gmail.com

*Journal of Cheminformatics* 2016, **8(Suppl 1)**:O12

Statistical modeling (also termed QSAR/QSPR) is a general name for a host of methods that correlate a specific activity for a set of compounds with their structure-derived descriptors by means of a mathematical model. The method has been widely applied in many fields including chemistry, biology, and environmental sciences with a particular emphasis in drug design.

In recent years less “traditional” QSAR studies have emerged focusing for example on the design of food, cosmetics and oil products, high energy materials and solar cells. These QSAR models are often referred to as M(material)QSAR and form part of the now growing field of Material Informatics.

This seminar will focus on the application of statistical modeling techniques in material sciences discussing, through selected applications, methods typically used in this field (e.g., PCA, Clustering and linear and non-linear regression), material descriptors (e.g., material composition, material spectra which are particularly useful when the exact composition / structure of the material are unknown) and the challenges in obtaining them. Special emphasis will be put on the application of MQSAR in the design of photovoltaic (PV) cells of various types. In particular cells entirely made of metal oxides (MO) have the potential to provide clean and affordable energy if their power conversion efficiencies are improved. Such improvements require the development of new MOs which in turn could benefit from combining combinatorial material sciences for producing solar cells libraries with statistical tools to direct synthesis efforts. For this purpose we developed a QSAR workflow with several novel components [1,2] and applied it to the analysis of a diversity of solar cell libraries [3]. Our results demonstrate that MQSAR models with good prediction statistics for multiple solar cells properties could be developed and that these models highlight important factors affecting these properties in accord with experimental findings. The resulting models are therefore suitable for designing better solar cells. We further demonstrate that the similar property principle commonly invoked in pharmaceuticals design could be extended to PV cells.

**References**

1. Yosipof A, Senderowitz H: Optimization of molecular representativeness. J Chem Inf Model. 2014;54:1567–77.

2. Yosipof A, Senderowitz H. k-Nearest Neighbors optimization based outlier removal. J Comput Chem 2015;36:493–506.

3. Yosipof A, Nahum OE, Anderson AY, Barad H, Zaban A, Senderowitz H. Data mining and machine learning tools for combinatorial material science of all-oxide photovoltaic cells. Mol Inf 2015;34:367–79.

## O13 Development of methods for the efficient comparison of protein binding sites

### Timo Krotzky

#### Department of Pharmaceutical Chemistry, University of Marburg, Germany

##### **Correspondence:** Timo Krotzky - krotzky@uni-marburg.de

*Journal of Cheminformatics* 2016, **8(Suppl 1)**:O13

Determination of similarities between protein binding pockets is an important challenge in computer-aided drug design. To this end, Cavbase was developed as a tool for the automated detection, storage, and classification of putative protein binding sites [1]. Binding sites are characterized as sets of pseudocenters, which denote surface-exposed physicochemical properties, and can be used to enable mutual binding site comparisons. However, these comparisons tend to be computationally very demanding and often lead to very slow computations of the similarity measures.

In this contribution, improved and accelerated methods for the comparison of protein binding sites are presented. We propose a novel and efficient modeling formalism that does not increase the size of the graph model used in Cavbase, but leads to graphs containing considerably more information assigned to the nodes, due to the introduction of additional descriptors which consider local surface characteristics. Combined with a heuristic for the efficient detection of maximum common subgraphs, this leads to a gain of information and enables much faster but still very accurate comparisons between different structures. Moreover, another accelerated version is discussed which makes use of graph partitioning [3]. Therefore, graphs are split into disjoint components with regard to pseudocenter types prior to their comparisons. This leads to seven much smaller graphs than the original one and thus to another significant speed-up with only a small loss of accuracy.

Finally, a program is introduced, which allows for ultra-fast similarity comparisons, as protein binding sites are represented by sets of distance histograms that are both generated and compared with linear complexity [4]. This method attains a speed of more than 20,000 comparisons per second, which makes screenings across large datasets and even entire databases easily feasible. The practical use of the new methods is proven by a successful prospective virtual screening study that aimed at the identification of novel inhibitors of the NMDA receptor.

**References**

1. Schmitt S, Kuhn D, Klebe G. A new method to detect related function among proteins independent of sequence and fold homology. J Mol Biol. 2002;323:387–406.

2. Krotzky T, Fober T, Hüllermeier E, Klebe G. Extended graph-based models for enhanced similarity search in Cavbase. IEEE/ACM Trans Comput Biol Bioinf 2014;11:878–90.

3. Krotzky T, Klebe G. Acceleration of binding site comparisons by graph partitioning. Mol Inform. in press.

4. Krotzky T, Grunwald C, Egerland U, Klebe G. Large-scale mining for similar protein binding pockets: with RAPMAD retrieval on the fly becomes real. J Chem Inf. Model. 2015;55:165–79.

## O14 Fragment-based design of viral protease inhibitors by virtual screening and chemical space sampling

### Robert Schulz, Gerhard Wolber

#### Pharmaceutical and Medicinal Chemistry, Institute of Pharmacy, Freie Universität Berlin, Königin-Luise Straße 2+4, 14195 Berlin, Germany

##### **Correspondence:** Robert Schulz - robert.schulz8@fu-berlin.de

*Journal of Cheminformatics* 2016, **8(Suppl 1)**:O14

Fragment-based methodologies have become an alternative to conventional High Throughput Screening during the last decade [1]. One of the advantages is the higher hit rate due to the much smaller fragment space. Identified hits not only serve as starting points for drug design but also represent sweet spots in the chemical space. Further development can therefore be promoted by sampling the chemical around the fragment hits.

This was applied to evolve fragment hits identified through a 3D pharmacophore-based virtual screening [2] against the viral 3C protease via fragment growing. At first fragment growing was carried out through a de novo design [3] workflow in which the fragment hit core is combined with tangible chemical building blocks via organic synthesis reactions encoded as SMIRKS patterns to ensure synthetic feasibility later on. To select de novo designed fragment hit analogues for chemical synthesis the generated library was analyzed through virtual screening for target binding along fragment growing vectors defined through structure-based design and for chemical space population by principle component analysis. A subset with promising binding characteristics was selected for chemical synthesis and subsequent biological evaluation.

In this work we show how fragment growing can be supported by efficient chemical space sampling to suggest promising de novo designed analogues, which are synthetically feasible and thus enable rapid fragment growing.

**References**

1. Erlanson DA. Introduction to fragment-based drug discovery. Top Curr Chem. 2012;317:1–32.

2. Wolber G, et al. Strategies for 3D pharmacophore-based virtual screening. Drug Discov Today Technol. 2010;7:e203–70.

3. Schneider G. De novo design—hop(p)ing against hope. Drug Discov Today Technol. 2013;10:e453–60.

## O15 Identification and preprocessing of alternative protein binding site conformations for modeling protein flexibility

### Stefan Bietz, Matthias Rarey

#### Center for Bioinformatics, University of Hamburg, Hamburg, 20146, Germany

##### **Correspondence:** Stefan Bietz - bietz@zbh.uni-hamburg.de

*Journal of Cheminformatics* 2016, **8(Suppl 1)**:O15

Modelling protein flexibility is still a highly challenging objective in various fields of computer aided drug design. Many applications make use of structure ensembles as a convenient way for incorporating structural variability of proteins. Due to the steadily growing amount of available protein structures, alternative conformations can meanwhile be provided for a multitude of different targets. However, ensemble generation is mostly a time-consuming process which often requires manual interaction. In order to simplify the usage of protein ensembles, we developed a fully automated preprocessing workflow for the enrichment and the analysis of protein flexibility information [1]. In a first step, alternative conformations of a given binding site are extracted from an arbitrary selection of protein structures, e.g. the current version of the Protein Data Base (PDB). Using an indexed database that has been specially geared to this purpose makes this step highly efficient. Afterwards, the resulting structures are being aligned with our new active site alignment algorithm ASCONA [2] which has been developed with a focus on an accurate detection of protein backbone variations. Based on the active site alignment, a further step detects flexible regions within the binding site and uses a remaining rigid core for a superimposition of the generated ensemble. If required, a subsequent filtering reduces the ensemble to a set of relevant protein conformations. Finally, the resulting ensemble structures are protonated with our hydrogen prediction tool Protoss [3], considering tautomerism and protonation states of both protein and ligand molecules. In summary, our preprocessing workflow constitutes a very convenient, reliable, and particularly fast way to generate structural ensembles for any application of interest.

**References**

1. Bietz S, Rarey M. SIENA: Efficient compilation of selective protein binding site ensembles. J Chem Inf Model. submitted.

2. Bietz S, Rarey M. ASCONA: Rapid detection and alignment of protein binding site conformations. J Chem Inf Model. 2015;55:1747–56.

3. Bietz S, Urbaczek S, Schulz B, Rarey M. Protoss: a holistic approach to predict tautomers and protonation states in protein-ligand. J Cheminf. 2014;6:12.

## O16 ChemPLPXB: implementation of QM-based terms for the recognition of halogen bonding in drug design

### Markus O. Zimmermann, Andreas Lange, Manuel Ruff, Johannes Heidrich, Ionut Onlia, Thomas E. Exner, Frank M Boeckler

#### Department of Pharmaceutical and Medicinal Chemistry, Eberhard Karls University Tuebingen, Germany

##### **Correspondence:** Markus O. Zimmermann - frank.boeckler@uni-tuebingen.de

*Journal of Cheminformatics* 2016, **8(Suppl 1)**:O16

Halogen bonding is rapidly gaining recognition in medicinal chemistry and related fields with a concomitant need for reliable evaluation of the quality of the interaction [1]. Several MM parameterizations and QM/MM methods have been recently developed to facilitate the study of these interactions. We extensively used QM model calculations on the MP2/TZVPP-level of theory to systematically map the relationship between strength and geometry of halogen bonds to different interaction partners (carbonyl backbone, sulfur contacts, nitrogen contacts, carboxylates, and π-systems) [2–4]. We evaluated the potential for molecular design of additional halogen bonds in existing protein-ligand complexes of the PDB by applying XBScore, our first QM-derived scoring function for the recognition of contacts to the carbonyl backbone [5]. This approach has been experimentally validated on the protein kinase p38α with halogenated ligands using DSF and FP assays. In addition, we used support vector regression to develop a QM-based scoring function for the recognition of halogen bonds targeting methionine. We integrated both scoring functions in ChemPLP and investigated their potential to improve pose retrieval for a test set of crystal structures featuring halogen bonds.

**References**

1. Wilcken R, Zimmermann MO, Lange A, Joerger AC, Boeckler FM. Principles and applications of halogen bonding in medicinal chemistry and chemical biology. J Med Chem. 2013;56:1363–1388.

2. Wilcken R, Zimmermann MO, Lange A, Zahn S, Kirchner B, Boeckler FM. Addressing methionine in molecular design through directed sulfur-halogen bonds. J. Chem Theory Comput. 2011;7:2307–15.

3. Wilcken R, Zimmermann MO, Lange A, Zahn S, Boeckler FM. Using halogen bonds to address the protein backbone: a systematic evaluation. J Comput Aided Mol Des. 2012;26:935–45.

4. Lange A, Zimmermann MO, Wilcken R, Zahn S, Boeckler FM. Targeting histidine side chains in molecular design through nitrogen–halogen bonds. J Chem Inf Model. 2013;53:3178–89.

5. Zimmermann MO, Lange A, Boeckler FM. Evaluating the potential of halogen bonding in molecular design: automated scaffold decoration using the new scoring function XBScore. J Chem Inf Model. 2015;55:687–99.

## O17 Dynamic and mechanistic models for the ligand-dependant modulation of G protein coupled receptors

### Marcel Bermudez, Gerhard Wolber

#### Computer-Aided Drug Design, Institute of Pharmacy, Freie Universität Berlin, 14195 Berlin

##### **Correspondence:** Marcel Bermudez - m.bermudez@fu-berlin.de

*Journal of Cheminformatics* 2016, **8(Suppl 1)**:O17

Computational approaches have become indispensable for drug design campaigns but also as auxiliary tool for structural biology [1]. In the field of G protein coupled receptors (GPCRs), the combination of *in silico* methods and pharmacological experiments represent a strong alliance for novel functional insights. Recent achievements in GPCR crystallography provide us with new structural data on GPCRs. However, these structures represent only single static views of highly flexible proteins. The combination of crystallographic data with state-of-the-art computer-driven simulations allows for a mechanistic view on this highly important class of drug targets. Taking muscarinic acetylcholine receptors (MAChRs) as representative examples we explained, how GPCRs can be modulated in a ligand dependent and predictable manner.

Starting from carefully developed homology models of MAChRs, extensive binding mode analyses were performed by means of protein-ligand docking and 3D pharmacophore modeling. In order to sample the flexibility and the dynamic properties of the receptor-ligand complexes we carried out all-atom molecular dynamics simulations [2]. This combination led to mechanistic GPCR models that comprise both inactive and active-like receptor states. After characterization of the orthosteric binding pocket, we focused on *dualsteric ligand binding* [3]. Such ligands simultaneously bind to the orthosteric and the allosteric binding site and combine the high affinity of orthosteric ligands with the high specificity of the allosteric binding site.

We show, for the first time, the structural basis for dualsteric GPCR modulation on a molecular level. Our functional GPCR models are able to rationalize subtype selectivity and biased signaling. Additionally, our models explain a novel concept for partial agonism, termed dynamic ligand binding. Taken together, our dynamic models illustrate how distinct conformational states can be stabilized in a ligand-dependent manner. This offers the possibility to rationally design specific modulators for MAChRs but also for other GPCRs.

**References**

1. Bermudez M, Wolber G. Structure versus function—the impact of computational methods on the discovery of specific GPCR–ligands. Bioorg Med Chem. 2015;23:3907–12.

2. Bermudez M, et al. Structural characteristics of the allosteric binding site represent a key to subtype selective modulators of muscarinic acetylcholine receptors. Mol Inf. 2015;e-pub ahead of print. doi:10.1002/minf.201500025.

3. Schmitz J, et al. Dualsteric muscarinic antagonists–orthosteric binding pose controls allosteric subtype selectivity. J Med Chem. 2014;57:6739–50.

## O18 Computer design of ionic liquids for CO_2_ absorption

### Dzmitry S. Firaha, Oldamur Hollóczki, Barbara Kirchner

#### Mulliken Center for Theoretical Chemistry, University of Bonn, Bonn, Germany, 53115, Germany

##### **Correspondence:** Barbara Kirchner - kirchner@thch.uni-bonn.de, Oldamur Hollóczki - holloczki@gmail.com

*Journal of Cheminformatics* 2016, **8(Suppl 1)**:O18

**Fig. 3:**
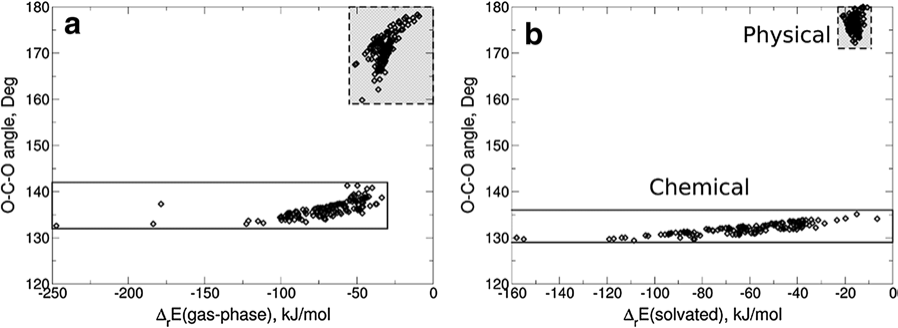
Binding energy versus O-C-O angle for the anion-CO_2_ complexes optimized in gas-phase (A) and including continium solvation model (B)

CO2 absorption in ionic liquids (ILs)—which are suitable solvents with tunable properties—caught great interest of scientists in the last decade. Having a huge variety of possible ILs (~10^15^) the CO_2_ solubility can vary strongly with selected ions. In order to save the time for choosing the most promising IL candidates from the experiment, we suggest simple theoretical protocols to predict the CO_2_ absorption behaviour of ionic liquids [1]. It was found that strong interaction with the anions of the IL, i.e., the formation of carboxylates with O-C-O angles <140°, corresponds chemical absorption whereas weak interaction (O-C-O angle >170°) indicates physical absorption (Fig. 3). A predictive estimate with a clear exact distinction between physical and chemical absorption can be simply obtained by carrying out the geometry optimization in the presence of a solvation model instead of optimizing it only in gas phase as has been done so far. From these solvated geometries free energies are derived which compare very well with experiment [2]. We also correlate the calculated energies with experimental gas capacities (mol CO_2_ per mol IL), in order to estimate the potential capacity close to the room temperature. Within the suggested protocol the most promising anions, potentially useful to design ionic liquids for reversible chemical absorption of CO_2_, are defined by the reaction Gibbs free energy in a range from −30 to 16 kJ mol^−1^.

**References**

1. Firaha DS, Hollóczki O, Kirchner B. In silico design of ionic liquids as CO_2_ absorbent. Angew Chem Int Ed. 2015;accepted.

2. Seo S, Quiroz-Guzman M, DeSilva MA, Lee TB, Huang Y, Goodrich BF, Schneider WF, Brennecke JF. Chemically tunable ionic liquids with aprotic heterocyclic anion (AHA) for CO_2_ capture. J Phys Chem. B 2014;118:5740–51.

## O19 Molecular basis for the long duration of action and kinetic selectivity of tiotropium for the muscarinic M3 receptor: insights from molecular dynamics simulations

### Christofer S. Tautermann

#### Boehringer Ingelheim Pharma GmbH & Co. KG, Lead Identification and Optimization Support, Birkendorfer Str. 65, D-88397 Biberach a.d. Riss, Germany

##### **Correspondence:** Christofer S. Tautermann

*Journal of Cheminformatics* 2016, **8(Suppl 1)**:O19

Antagonizing the human M3 muscarinic receptor (hM3R) over a long time is a key feature of modern bronchodilating COPD drugs aiming at symptom relief. The long duration of action of the antimuscarinic drug tiotropium and its kinetic subtype selectivity over hM2R have been investigated by kinetic mapping of the binding site and the exit channel of hM3R. Hence, dissociation experiments have been performed with a set of molecular matched pairs of tiotropium on a large variety of mutated variants of hM3R. The exceedingly long half-life of tiotropium (of more than 24 h) is attributed to interactions in the binding site; particularly a highly directed interaction of the ligands’ hydroxy group with an asparagine (N508^6.52^) prevents rapid dissociation via a snap-lock mechanism. The kinetic selectivity over hM2R, however, is caused by differences in the electrostatics and in the flexibility of the extracellular vestibule.

Extensive molecular dynamics (MD) simulations based on the M3R X-ray structure are performed and various unexpected changes in receptor flexibility and solvent networks upon ligand binding are seen. Minor changes in the ligand structure can lead to breathing-motions on long time-scales of the entry channel in hM3R and only the most optimized ligand (i.e. tiotropium) tends to freeze all large scale movements. Similar effects are seen in MD simulations of mutated receptor variants. These observations may help to understand differences in residence times of various ligands. Investigations like this may prove to be useful to qualitatively predict differences in receptor residence times for structurally closely related ligands.

Putting together the pieces: building a reaction-centric electronic lab notebook for mobile devices.

## O20 Prioritization of less-explored protein kinases as drug targets

### Andrea Volkamer^1^, Sameh Eid^1^, Samo Turk^1^, Friedrich Rippmann^2^, Simone Fulle^1^

#### ^1^BioMed X Innovation Center, Im Neuenheimer Feld 583, 69120 Heidelberg, Germany; ^2^Merck KGaA, Merck Serono, Global Computational Chemistry, Frankfurter Str. 250, 64293 Darmstadt, Germany

##### **Correspondence:** Andrea Volkamer - volkamer@bio.mx, Simone Fulle - fulle@bio.mx

*Journal of Cheminformatics* 2016, **8(Suppl 1)**:O20

Protein kinases are involved in a variety of diseases including cancer, inflammation, and autoimmune disorders. Thus, the development of new kinase inhibitors has been a major focus in pharmaceutical research over the last decades. Although, this resulted to date in 25 FDA approved drugs, only a small subset of kinases are established key targets, while most kinase inhibitors are unintentionally promiscuous.

Using the wealth of available kinase structures—i.e. over 3600 PDB structures—we analysed the druggability of the entire human kinome and prioritized (yet) untapped kinases for drug discovery efforts [1]. For this, representative kinase structures were selected and the missing part of the kinome was modelled via homology modelling. DoGSiteScorer [2] was used to calculate geometrical and physicochemical properties of the ATP pockets and to predict the potential of each kinase to be druggable. The results indicate that—from a structural perspective—around 75% of the kinome have binding site characteristics that should allow the design of drug-like compounds. Top ranking structures comprise kinases that are primary targets of known approved drugs but additionally point to so far less explored kinases.

Several aspects will be discussed including the top ranking kinases, the information gained from experimental hit rates [3] as well as our attempts to use structural information to enhance compound selectivity.

**References**

1. Volkamer A, Eid S, Turk S, Jaeger S, Rippmann F, Fulle S: Pocketome of human kinases: prioritizing the ATP binding sites of (yet) untapped protein kinases for drug discovery. J Chem Inf Model. 2015;55:538–49.

2. Volkamer A, Kuhn D, Grombacher T, Rippmann F, Rarey M. Combining global and local measures for structure-based druggability predictions. J Chem Inf Model. 2012;52:360–72.

3. Bento P, et al. The ChEMBL bioactivity database: an update. Nucleic Acids Res. 2014;42:D1083–90.

## O21 A three-site mechanism for agonist/antagonist action on the vasopressin receptors

### Noureldin Saleh^1^, Giorgio Saladino^2^, Francesco L. Gervasio^2^, Elke Haensele^3^, Lee Banting^3^, David C. Whitley^3^, Jana Sopkova-de Oliveira Santos^4^, Ronan Bureau^4^, Timothy Clark^1,3^

#### ^1^Computer-Chemie-Centrum and Interdisciplinary Center for Molecular Materials Friedrich-Alexander-Universität Erlangen-Nürnberg, Nägelsbachstraße 25, 91052 Erlangen, Germany; ^2^Department of Chemistry and Institute of Structural and Molecular Biology, University College London, London WC1E 6BT, United Kingdom; ^3^Centre for Molecular Design, School of Pharmacy and Biomedical Sciences, University of Portsmouth, St Michael’s Building, White Swan Road, Portsmouth PO1 2DT, United Kingdom; ^4^Centre d’Etudes et de Recherche sur le Médicament de Normandie, UPRES EA 4258—FR CNRS 3038 INC3M, Boulevard Becquerel, 14032 CAEN Cedex, France

##### **Correspondence:** Noureldin Saleh - noureldin.saleh@fau.de

*Journal of Cheminformatics* 2016, **8(Suppl 1)**:O21

Extensive classical molecular-dynamics simulations including metadynamics enhanced sampling reveal three distinct binding sites for arginine vasopressin (AVP) at its V_2_-receptor (V_2_R). Two of these, the vestibule and intermediate sites, block (antagonize) the receptor and the third is the orthosteric activation (agonist) site. The contacts found for the orthosteric site satisfy all the requirements deduced from mutagenesis experiments, including the involvement of residues near the extracellular N-terminus of the receptor. The biologically active conformation of AVP has been determined for each binding site. Metadynamics simulations on V_2_R and its V_1a_R-analog give an excellent correlation with experimental binding free energies by assuming that the most stable binding site in the simulations corresponds to the experimentally determined binding free energy in each case. We extended our results to the β_2_-adrenergic receptor to study the co-operative mechanism of the ligand and G-protein on GPCR activation. The resulting three-site mechanism for both β_2_ and V_2_-activity is compatible with the ternary complex model.

## O22 Analysis of µs MD simulations of the p53 core domain

### Achim Sandmann^1^, Harald Lanig^2^, Timothy Clark^3^

#### ^1^Bioinformatics, Institute for Biochemistry, FAU Erlangen-Nürnberg, Fahrstr. 17, Erlangen, 91054 Germany; ^2^Central Institute for Scientific Computing (ZISC), FAU-Erlangen-Nürnberg, Martensstr. 5a, 91058 Erlangen, Germany; ^3^Department of Chemistry and Pharmacy, Computer Chemistry Center, FAU Erlangen-Nürnberg, Naegelsbachstr. 25, 91052 Erlangen, Germany

##### **Correspondence:** Achim Sandmann - Achim.Sandmann@fau.de

*Journal of Cheminformatics* 2016, **8(Suppl 1)**:O22

Increased computational performance makes possible MD simulations of biologically relevant timescales exceeding 1 µs, which can be used to sample the conformational space of proteins. Of the available methods, the brute force method has the advantage of being highly analogous to the actual processes and of using little *a priori* knowledge [1]. In this work, we sought methods that can be useful for interpreting the vast amount of data associated with these kinds of simulations.

The system under investigation, the p53 core domain, has several loop regions protruding from a stable β-barrel that B-factor plots suggest to be flexible. Cluster analysis of simulations several microseconds long showed that no structural convergence is reached for the system. Subsequently, the flexible regions were investigated individually.

A set of cluster analyses were performed, in which only residues of one individual region were regarded in each. This vastly simplified data allowed reoccurring structures to be identified, while it also showed that no convergence is reached for the individual regions. Structural homogeneity within clusters, and structural differences between clusters with high RMSD differences of those individual regions, can be shown by hydrogen-bond analyses. They can also show thermodynamic explanations for sudden changes in the RMSD trajectory of the different regions. Clustering of the flexible loop regions using a DASH analysis [2], which is based on torsional angles, showed a good match to the results of Cartesian clustering based on atomic positions. Correlation analysis showed that flexible regions are mostly isolated by the β-barrel and move independently as long as they have no direct contacts and none of the regions makes a large move.

These analyses show that regarding flexible regions individually can improve the evaluation of the incompletely sampled conformational space of a protein. This knowledge can then be used when it is compared with a similar system like a mutated or complex-bound version of the protein.

**References**

1. Zwier MC, Chong LT. Reaching biological timescales with all-atom molecular dynamics simulations. Curr Opin Pharmacol. 2010;10:745–52.

2. Salt DW, Hudson BD, Banting L, Ellis MJ, Ford MG. DASH: a novel analysis method for molecular dynamics simulation data. Analysis of ligands of PPAR-γ. J Med Chem. 2005;48:3214–20.

## O23 Theoretical approach to conformational preconfiguration of drug-like molecules in solution

### Patrick Kibies^1^, Jochen Heil^1^, Franziska Hoffgaard^1^, Roland Frach^1^, Julian Engel^2^, Steven Smith^2^, Debjit Basu^2^, Daniel Rauh^2^, Stefan M. Kast^1^

#### ^1^Physikalische Chemie III, TU Dortmund, 44227 Dortmund, Germany; ^2^Chemische Biologie, TU Dortmund, 44227 Dortmund, Germany

##### **Correspondence:** Patrick Kibies - patrick.kibies@tu-dortmund.de

*Journal of Cheminformatics* 2016, **8(Suppl 1)**:O23

Accurate assessment of the conformational space of drug-like molecules in free solution is a frequently underestimated, yet relevant ingredient of molecular design. In particular, predicting and controlling free ligand conformations is essential for minimizing the entropic penalty to reorganize a ligand’s geometry upon binding to a protein. Overcoming the deficiencies of common small molecule force fields represents a particular challenge due to the considerable computational cost of high-level quantum-chemical calculations for predicting the conformational manifold.

Here, we demonstrate the performance of a hierarchical filtering scheme that allows for the identification of dominant conformations together with their proper statistical weights measured by their free energies in solution with quantum-chemical accuracy. The automated workflow implies a sequence of force field-based high-temperature molecular dynamics simulations using implicit solvent models, clustering and filtering steps, and high-level geometry optimizations in solution employing the polarizable continuum model (PCM) as well as the embedded cluster reference interaction site model (EC-RISM) [1] for scoring, and calculation of theoretical NMR spectra [2] to be compared with experiments. We apply the workflow to variants of the protein kinase inhibitor WZ4002 and several novel EGFR inhibitors that are highly active against a drug-resistant mutant of the epidermal growth factor receptor (EGFR-T790M) [3–5]. The relative significance of conformational preconfiguration in comparison with modulation of direct protein-ligand interactions upon chemical substitution is discussed.

**References**

1. Kloss T, Heil J, Kast SM. Quantum chemistry in solution by combining 3d integral equation theory with a cluster embedding approach. J Phys Chem B 2008;112:4337–43.

2. Frach R, Kast SM. Solvation effects on chemical shifts by embedded cluster integral equation theory. J Phys Chem *A* 2014;118:11620–28.

3. Zhou W, et al. Novel mutant-selective EGFR Kinase inhibitors against EGFR T790M. Nature 2010;462:1070–4.

4. Zhou W, et al. Discovery of selective irreversible inhibitors for EGFR-T790M. Bioorg Med Chem Lett. 2011;21:638–43.

5. Engel J, et al. Targeting drug resistance in EGFR with covalent inhibitors: a structure-based design approach. J Med Chem. 2015;58:6844–63.

## O24 Using support vector regression to develop a quantum chemical-based scoring function for the recognition of halogen bonds targeting methionine

### Manuel Ruff^1,2^, Markus O. Zimmermann^1,2^, Andreas Lange^1,2^, Ionut Onlia^1,2^, Oliver Kohlbacher^2^, Thomas E. Exner^1,2^, Frank M. Boeckler^1,2^

#### ^1^Department of Pharmaceutical and Medicinal Chemistry, Institute of Pharmaceutical Sciences, Eberhard Karls University Tübingen, Germany; ^2^Center for Bioinformatics Tuebingen (ZBIT), Eberhard Karls University Tuebingen, Germany

##### **Correspondence:** Frank M. Boeckler - frank.boeckler@uni-tuebingen.de

*Journal of Cheminformatics* 2016, **8(Suppl 1)**:O24

Halogen bonding is a rather new type of non-classical interaction. Recently, the field of halogen bonding has attracted much attention in life sciences and drug discovery [1–4] and various halogen bonding examples have been reported and shown to be viable. Therefore, halogen bonding needs to be integrated into the molecular and drug design process. This talk presents a new scoring function for the halogen bonding interaction between halogenated molecules and the side chain sulfur of methionine based on quantum chemical calculations using the MP2/TZVPP level of theory. For this purpose, we have extended our previously reported QM studies quite significantly [5] by an exhaustive, systematical generation and evaluation of interaction geometries using small model systems. From this data, we derived two separate scoring terms for the interaction using support vector regression and 4-fold cross validation: a σ-hole factor and a SphericalScore. The σ-hole factor describes the directionality of the interaction and the SphericalScore addresses the spatial position of the halogenated ligand around the sulfur atom. A combination of these two scoring terms yields the overall halogen bonding score. Validation was done through randomly generated interaction geometries not contained in the original data. Concomitant evaluation of these geometries through QM calculations and prediction through the scoring function showed very good correlations. The herein presented scoring function is a blueprint for integration into empirical scoring functions and docking programs.

**References**

1. Wilcken R, Zimmermann MO, Lange A, Joerger AC, Boeckler FM: J Med Chem. 2013;56:1363–1388.

2. Zimmermann MO, Lange A, Wilcken R, Cieslik MB, Exner TE, Joerger AC, Koch P, Boeckler FM. Future Med Chem. 2014;6:617–39.

3. Wilcken R, Liu X, Zimmermann MO, Rutherford TJ, Fersht AR, Joerger AC, Boeckler FM. J Am Chem Soc. 2012;134:6810–18.

4. Zimmermann MO, Lange A, Boeckler FM. J Chem Inf Model 2015;55:687–699.

5. Wilcken R, Zimmermann MO, Lange A, Zahn S, Kirchner B, Boeckler FM. J Chem Theory Comput. 2011;7:2307–2315.

## O25 Recent progress in exploring the role of water in protein-ligand binding

### Jonathan W. Essex^1^, Michael S. Bodnarchuk^2^, Gregory A. Ross^1^

#### ^1^School of Chemistry, University of Southampton, Southampton, SO17 1BJ, UK; ^2^School of Mechanical Engineering, Imperial College London, London, SW1 2AZ, UK

##### **Correspondence:** Jonathan W. Essex - jwe1@soton.ac.uk; Gregory A. Ross - G.A.Ross@soton.ac.uk

*Journal of Cheminformatics* 2016, **8(Suppl 1)**:O25

Water molecules play integral roles in the formation of many protein-ligand complexes, and recent computational efforts have been focused on predicting the thermodynamic properties of individual waters and how they may be exploited in rational drug design. However, when water molecules form highly coupled hydrogen bonding networks, there is, as yet, no method that can rigorously calculate the free energy to bind the entire network, or asses the degree of cooperativity between waters.

In this work, we revisit the grand canonical Monte Carlo simulation technique [1] and show how it can be used to calculate efficiently the binding free energies of water networks of arbitrary size and complexity. Using a single set of simulations, our methods can locate waters, estimate their binding affinities, capture the cooperativity of the water network, and evaluate the hydration free energy of entire protein binding sites. Our techniques have been applied to multiple test systems and compare favourably to thermodynamic integration simulations and experimental data. The implications of these methods in drug design will be discussed.

**Reference**

1. Adams DJ. Chemical potential of hard-sphere fluids by Monte-Carlo methods. Mol Phys. 1974;28:1241–52.

## O26 Ab initio derived descriptors as a promising perspective for regioselectivity prediction of metabolic reactions

### Arndt R. Finkelmann^1^, Andreas H. Göller^2^, Gisbert Schneider^1^

#### ^1^Swiss Federal Institute of Technology (ETH), Institute of Pharmaceutical Sciences, 8093 Zürich, Switzerland; ^2^Bayer Pharma AG, Global Drug Discovery, 42096 Wuppertal, Germany

##### **Correspondence:** Arndt R. Finkelmann -arndt.finkelmann@pharma.ethz.ch

*Journal of Cheminformatics* 2016, **8(Suppl 1)**:O26

The complexity and diversity of chemical transformations involved in drug metabolism renders Site of Metabolism (SoM) prediction difficult [1]. One strategy to increase performance of machine-learning models is to develop appropriate molecular representations in terms of new descriptors. Quantum-chemically derived molecular descriptors encoding the reactivity of individual atoms appear to be an intuitive starting-point for model improvements. Here, we address the challenge of SoM prediction with descriptors based on atomic partial charges. To this end we assessed various partial charge schemes with respect to their dependence on quantum mechanical methods as well as their dependence on molecular conformation and charge state [2]. Based on the results of this study, we implemented various atomic reactivity descriptors derived from the atomic charge neighborhood information. These descriptors performed well for the task of predicting cytochrome P450 SoMs in drug molecules. The methodological concept is generally applicable to reactivity prediction and not limited to metabolism.

**References**

1. Kirchmair M, Göller AH, Lang D, Kunze J, Testa B, Wilson ID, Glen RC, Schneider G. Predicting drug metabolism: experiment and/or computation? Nat Rev Drug Discov. 2015;doi:10.1038/nrd4581.

2. Finkelmann AR, Göller AH, Schneider G. Manuscript in preparation.

## O27 Integrating Chemoinformatics and Quantum Chemistry for computational electrochemistry: the search for new electrolyte materials

### Tamara Husch^1^, Christoph Schütter^2^, Andrea Balducci^2^, Martin Korth^1^

#### ^1^Institute for Theoretical Chemistry, Ulm University, 89081 Ulm, Germany; ^2^Helmholtz Institute Ulm, Karlsruhe Institute of Technology, 89081 Ulm, Germany

##### **Correspondence:** Martin Korth - martin.korth@uni-ulm.de

*Journal of Cheminformatics* 2016, **8(Suppl 1)**:O27

Molecular materials are a crucial component of electrochemical storage devices like Lithium ion batteries [1]. Electrolyte solvents for instance have a strong influence on all relevant properties of the electrolyte, which in turn has great impact on the performance of the storage device. Physical limitations of electrolyte solvents are more and more often found to be roadblocks for the further improvement of batter technology [1]. Despite this, only a tiny fraction of the large number of possible solvents has been experimentally investigated, because experimental high-throughput work is complicated and costly. Computational work at the atomic scale on the other hand is still in the early stages in this field [2]. Like many parts of materials science, battery research is dominated by Solid State Physics, though in the case of molecular materials, methods from Quantum Chemistry and Chemoinformatics offer many much needed opportunities. We have made first steps to integrate existing computational approaches from these fields as well as Chemical Engineering within a tool-box for the design of optimization of liquid electrolyte systems as a filter for subsequent experimental work [3–5]. In addition, combinatorial Quantum Chemistry based tools were developed to estimate complex properties related to the reactivity of electrolyte materials with each other and with electrode materials. We have then investigated the known chemical space and complete subspaces of the most promising compound classes for new electrolyte materials.

**References**

1. Xu K. Nonaqueous liquid electrolytes for lithium-based rechargeable batteries. Chem Rev. 2004;104:4303.

2. Korth M. Computational studies on solid electrolyte interphase formation. Chem Model. 2015;11:57.

3. Korth M. Large-scale virtual high-throughput screening for the identification of new battery electrolyte solvents: evaluation of electronic structure theory methods. Phys Chem Chem Phys. 2014;16:7919.

4. Husch T, Yilmazer ND, Balducci A, Korth M. Large-scale virtual high-througput screening for the identification of new battery electrolyte solvents: computing infrastructure and collective properties. Phys Chem Chem Phys. 2015;17:3394.

5. Schütter C, Husch T, Korth M, Balducci A. Toward New solvents for EDLCs: from computational screening to electrochemical validation. J Phys Chem C 2015;accepted.

## P1 Towards a unified natural products library from African medicinal plants

### Fidele Ntie-Kang^1,2^, Stefan Günther^3^, Wolfgang Sippl^2^, Luc Meva’a Mbaze^4^

#### ^1^Department of Chemistry, University of Buea, Buea, South West Region, Cameroon; ^2^Institut für Pharmazie, Martin-Luther University of Halle-Wittenberg, Halle, 06120, Germany; ^3^Institut für Pharmazeutische Wissenschaften, Universität Freiburg, Freiburg, 79104, Germany; ^4^Department of Chemistry, University of Douala, Douala, Littoral Region, Cameroon

##### **Correspondence:** Fidele Ntie-Kang - ntiekfidele@gmail.com

*Journal of Cheminformatics* 2016, **8(Suppl 1)**:P1

We have previously developed a 3D virtual screening library of >3,000 compounds derived from Central African flora [1,2] and evaluated the pharmacokinetics profile [3]. This work has been extended to include West Africa [4], North Africa and Southern Africa, as well as a small library of compounds with remarkable potencies from African flora [5], including anti-mycobacterial infections, malaria, onchocerciasis and cancer [6]. In this work, we present an SQL platform for searching natural products for drug discovery from African medicinal plants. Data has been collected from major natural products journals and PhD theses from university libraries. The unified library contains > 5,000 unique structures from all regions of the African continent. The known uses of the plant species in African Traditional Medicine (ATM) have been previously related with the measured biological activities of the isolated plant metabolites [7]. Apart from the pan-African natural products library [8], each compound in this library is linked to known biological activities, as well as several physicochemical properties that can be used to evaluate drug-likeness. 3D structures are available for virtual screening purposes and each compound was classified to a chemical class, known biological activities and the plant species from which the compound was originally isolated. All compounds are available for download, thus the present data supports computer-aided drug design (CADD) and virtual screening (VS) campaigns.

**References**

1. Ntie-Kang F, et al. CamMedNP: building the Cameroonian 3D structural natural products database for virtual screening. BMC Complement Altern Med. 2013;13:88.

2. Ntie-Kang F, et al. ConMedNP: a natural product library from medicinal plants in Central Africa. i 2014;4:409–419.

3. Ntie-Kang F, et al.: In silico drug metabolism and pharmacokinetic profiles of natural products from medicinal plants in the Congo Basin. In Silico Pharmacol. 2013;1:12.

4. Ntie-Kang F, et al. The uniqueness and therapeutic value of natural products from West African medicinal plants, part II: terpenoids, geographical distribution, drug discovery. RSC Adv. 2014;4:35348–70.

5. Ntie-Kang F, et al.. AfroDb: a select highly potent and diverse natural product library from African medicinal plants. PLoS ONE 2013;8:e78085.

6. Ntie-Kang F, et al. Molecular modeling of potential anticancer agents from African medicinal plants. J Chem Inf Model. 2014;54:2433–2450.

7. Zofou D, et al. Bioactive natural products derived from the Central African flora against neglected tropical diseases and HIV. Nat Prod Rep. 2013;30:1098–1120.

8. Ntie-Kang F, et al. Virtualizing the p-ANAPL library: a step towards drug discovery from African medicinal plants. PLoS ONE 2014;9:e90655.

## P2 Application of computer modeling in the evaluation of naturally occurring anticancer compounds from African flora

### Fidele Ntie-Kang^1,2^, Conrad V. Simoben^1^, Lydia L. Lifongo^1^, Wolfgang Sippl^2^, Luc Meva’a Mbaze^3^

#### ^1^Department of Chemistry, Chemical and Bioactivity Information Centre, University of Buea, Buea, South West Region, Cameroon; ^2^Institut für Pharmazie, Martin-Luther University of Halle-Wittenberg, Halle, 06120, Germany; ^3^Department of Chemistry, University of Douala, Douala, Littoral Region, Cameroon

##### **Correspondence:** Fidele Ntie-Kang - ntiekfidele@gmail.com

*Journal of Cheminformatics* 2016, **8(Suppl 1)**:P2

Cancer stands amongst the most common disease-related causes of death; with ~7.6 million deaths within the human population and is expected to worsen in some few decades [1]. Previous studies have shown that about 48% of anticancer drugs approved were either natural products (NPs) or directly derived from NP lead compounds by semi-synthesis [2]. Our goal is to develop a small potent and less toxic NP library for virtual screening within the African setting. Computer-aided drug design methods, has become a promising part of the drug discovery process nowadays. This study was focused on generating a 3D structural library of potential anticancer compounds from the African flora and to evaluate the “drug-likeness” and toxicity of the new compound library. Virtually screening and *in silico* toxicity assessment were carried in comparison with the dataset of Naturally Occurring Plant-based Anticancer Compound-Activity-Target (NPACT), comprising ~1,500 published naturally occurring plant-based compounds from around the world [3]. From this study, about 400 compounds have been identified from African flora and their drug-likeness properties evaluated in comparison with NPACT and DNP. A successful docking attempt was carried out for some fourteen selected known anticancer drug targets [4]. Pharmacophore-based virtual screening and *in silico* toxicity assessment have also been done. Pharmacophore models were validated through receiver operating characteristic (ROC) and Güner-Henry (GH) scoring methods [5], indicating that several of the models generated could be useful for the identification of potential anticancer agents from natural product databases. The validated pharmacophore models were used as 3D search queries for virtual screening of the AfroCancer, along with the NPACT. Additionally, the *in silico* assessment of toxicity of the two datasets was carried out by use of eighty eight (88) toxicity end points predicted by Lhasa’s expert knowledge-based system (Derek) [6], showing that only an insignificant proportion of the promising anticancer agents would be likely to show high toxicity profiles. Diversity analysis of the AfroCancer and NPACT datasets was carried out using the analysis of principal components [7].

**References**

1. WHO Media Centre. Fact Sheet No. 297, 2013. http://www.who.int/mediacentre/factsheets/fs297/en/index.html. Accessed 4 Feb 2014.

2. Newman DJ, Cragg GM. Natural products as sources of new drugs over the 30 years from 1981 to 2010. J Nat Prod. 2010;75:311–35.

3. Ntie-Kang F, et al. How “drug-like” are naturally occurring anti-cancer compounds? J Mol Model. 2014;20:1–13.

4. Ntie-Kang F, et al.: Molecular modeling of potential anticancer agents from African medicinal plants. J Chem Inf Model. 2014;54:2433–50.

5. Güner OF, Henry DR, Pharmacophore perception, development, and use in drug design. In Güner OF editors. In metric for analyzing hit lists and pharmacophores. La Jolla, IUL Biotechnology Series, International University Line; 2000. p. 191–212.

6. Ridings JE, et al. Computer prediction of possible toxic action from chemical structure: an update on the DEREK system. Toxicology 1996;106:267–79.

7. Wold S, et al. Principal component analysis. Chemom Intell Lab Syst. 1987;2:37–52.

## P3 Knowledge base development for the prediction of acute aquatic toxicity of chemicals

### Fidele Ntie-Kang^1,2^, Philip Judson^3^

#### ^1^Chemical and Bioactivity Information Centre, Department of Chemistry, University of Buea, Buea, South West Region, Cameroon; ^2^Institut für Pharmazie, Martin-Luther University of Halle-Wittenberg, Halle, 06120, Germany; ^3^Chemical Bioactivity Information Centre, Heather Lea, Bland Hill, Norwood, Harrogate HG3 1TE, UK

##### **Correspondence:** Fidele Ntie-Kang - ntiekfidele@gmail.com

*Journal of Cheminformatics* 2016, **8(Suppl 1)**:P3

There is widespread concern about the toxicological effects of man-made chemicals in the environment, including those of pharmaceutical products and their metabolites. There are databases of, and computer models to predict, aquatic narcosis and, in some cases, excess aquatic toxicity such as ECOSAR [1] but there remains a need for better sharing of human knowledge about the full range of environmental and human toxicological hazards. Computer programs such as Derek [2] are used to share human knowledge about mammalian toxicity and a proof of concept application, Eco-Derek, using the same technology to share knowledge about toxicity to the ciliate Tetrahymena pyriformis and human skin sensitisation has been described [3]. We are developing new alerts covering other species not included in the original knowledgebase for Eco-Derek. The knowledgebase currently contains 52 alerts, 207 patterns, 138 reasoning rules, 79 literature sources, 5 species and 6 toxicity endpoints. For mammalian toxicity, Derek reports confidence in predictions of activity, or likelihood that activity will be seen, using terms such as “probable”, “plausible”. Eco-Derek reports the estimated potency of toxicity of a query chemical to *T. pyriformis*, using terms such as “High”, “Moderate”, “Low”. It takes into account both narcosis and mechanism-based toxicity (often called “excess toxicity” in cases where it is more severe than narcosis). The new knowledge-based system for predicting environmental toxicity is being built using the Nexus platform from Lhasa Limited [4]. It is intended that the system will report both potency and confidence in predictions (e.g. “Predicted toxicity to fish: Moderate. Confidence in the prediction: High”). Nexus currently only supports reporting of confidence (likelihood). So, for the present, knowledge about toxicity to *T. pyriformis* contained in eco-Derek has been reworked and assessed in terms of confidence about its predictive reliability and incorporated into the new system. In addition to the knowledge base, a database covering the environmental toxicity of chemicals is being built, emphasising, but not restricted to, aquatic toxicity, with effort concentrated on data from African sources not previously easily-accessible electronically. The data are being studied to develop new rules for the knowledge base of the prediction system. Species covered include *Daphnia magna*, *Escherichia coli*, *Pseudokirchneriella subcapitata*, *Danio rerio*, *Pimephales promelas* etc. The prediction system will be demonstrated during the presentation if time allows, and will be available for demonstration during conference breaks. We have currently incorporated the Eco-Derek knowledge into Lhasa’s Nexus platform. The purpose of this project is to facilitate the exchange of knowledge about environmental toxicity between scientists. It is a collaborative effort between disciplines and across continents. The authors invite and encourage other scientists to become involved.

**References**

1. Reuschenbach P et al. ECOSAR model performance with a large test set of industrial chemicals. Chemosphere 2008;71:1986–95.

2. Ridings JE, et al. Computer prediction of possible toxic action from chemical structure: an update on the DEREK system. Toxicology 1996;106:267–79.

3. Payne MP, Button WG. Prediction of acute aquatic toxicity in Tetrahymena pyriformis—‘Eco-Derek’, a knowledge-based system approach. SAR & QSAR Environ Res. 2013;24:439–60.

4. Lhasa Limited, Granary Wharf House, 2 Canal Wharf, Leeds LS11 5PS, UK.

## P4 Modeling of the influence of oxygen and N_2_O on the chemical stage of radiobiological mechanism using Petri Nets

### Jiří Barilla^1^, Miloš V Lokajíček^2^, Hana Pisaková^2^, Pavel Simr^1^

#### ^1^Faculty of Science, J. E. Purkinje University in Usti nad Labem, Ústí nad Labem, 400 96, Czech Republic; ^2^Institute of Physics, Academy of Sciences of the Czech Republic, Praha, 182 21, Czech Republic

##### **Correspondence:** Jiří Barilla - Jiri.Barilla@ujep.cz

*Journal of Cheminformatics* 2016, **8(Suppl 1)**:P4

The modelling of the chemical stage of radiobiological mechanism may be very helpful in the study of the radiobiological effect of ionizing radiation when the water radicals formed by the densely ionizing ends of primary or secondary charged particles react with DNA molecules in living cells and damage them. These radicals arise in clusters that diffuse while the radicals react mutually or with other species (radiomodifiers) present in water medium; oxygen and N_2_O being very important. The proposed mathematical model has been created with the help of Continuous Petri nets. It enables us to describe and study the influence of both the main parallel processes: chemical reactions and diffusion of radical clusters. It is possible to study the time change of concentration of individual radicals during this diffusion process, which may be very helpful when the efficiency of different substances present in medium in the DNA damage (radiobiological effect) is to be studied. We have started to study the corresponding problem earlier; the contemporary result of chemical reactions and cluster diffusion having been described with the help of corresponding differential equations. The given model has been applied to the experimental data obtained for Co60 radiation (see [1]). Continuous Petri nets have been then used to simulate time dynamics of chemical stage under anoxic conditions (see [2]). Oxygen (if present) may act in two different directions: at small concentrations the interaction with hydrogen radicals prevails and final biological effect diminishes while at higher concentrations additional efficient oxygen radicals may be formed. Molecules N_2_O react then with hydrated electrons; the number of OH radicals increases, which results in a greater damage of DNA molecules.

**Acknowledgements:** This work was supported by the project LG130131 of Ministry of Education, Youth and Sports of the Czech Republic.

**References**

1. Barilla J, Lokajíček M, Pisaková H, Simr P. Analytical model of chemical phase and formation of DSB in chromosomes by ionizing radiation. Austral Phys Eng Sci Med. 2013.

2. Barilla J, Lokajíček M, Pisaková H, Simr P. Applying Petri nets to modeling the chemical stage of radiobiological mechanism. Phys. Chem. Solids 2015;78:127–36. doi:10.1016/j.jpcs.2014.11.016.

## P5 Prediction of the functional properties of electroceramic materials using chemoinformatics approaches

### Natalia Kireeva^1,2^, Alexandre Petrov^1,2,3^, Denis Ostroumov^1,2^, Vitaly P. Solov’ev^1^, Vladislav S. Pervov^3^

#### ^1^Frumkin Institute of Physical Chemistry and Electrochemistry RAS, Moscow, 119071, Russia; ^2^Moscow Institute of Physics and Technology, Dolgoprudny, Russia, 141700, Russia; ^3^Kurnakov Institute of General and Inorganic Chemistry, Moscow, 119071, Russia

##### **Correspondence:** Natalia Kireeva - nkireeva@gmail.com

*Journal of Cheminformatics* 2016, **8(Suppl 1)**:P5

Our research focuses on the development of chemoinformatics methods and algorithms to design new functional electroceramic materials. Ceramic materials are widely used in electrochemical devices such as oxygen separation membranes, solid oxide fuel cell (SOFC) cathodes, dielectric materials etc. Solid oxide fuel cells are of great interest as economical, clean and efficient power generation devices. Fuel cells have several advantages over conventional power generation techniques. They characterized by high-energy conversion efficiency and high power density. The propagation of mobile telecommunication technologies require the development of new ceramic materials that can be used as dielectric resonators and filters. Due to the difficult and time consuming process of conventional compound synthesis, the development of materials often joins experimental synthesis efforts with in silico materials design [1]. Studying materials with a complex phase structure as well as the prediction of the properties of materials obtained under different conditions are of particular interest.

In this study we propose methods and descriptors efficient for the assessment of “composition-structure-functional property” relationship. Involved approaches include complementary machine learning methods.

**Acknowledgements:** Financial support by Russian Foundation for Basic Research (Projects 15-03-09075 and 14-29-04084) and President Grant for Government Support of Young Russian Scientists (MК-1003.2014.3) are gratefully acknowledged.

**Reference**

1. Scott DJ et al. Functional Ceramic Materials Database: An Online Resource for Materials Research. J Chem Inf Model. 2008;48:449–55.

## P6 A new benchmarking dataset for conformer ensemble generators

### Nils-Ole Friedrich, Kai Sommer, Matthias Rarey, Johannes Kirchmair

#### University of Hamburg, Center for Bioinformatics, Hamburg, 20146, Germany

##### **Correspondence:** Johannes Kirchmair - kirchmair@zbh.uni-hamburg.de

*Journal of Cheminformatics* 2016, **8(Suppl 1)**:P6

The representation of the relevant conformational space of small molecules (e.g. drug-like molecules) is a thoroughly studied, non-trivial problem. A wide variety of different methodologies have been explored, with the aim to devise an algorithm that achieves optimum performance with respect to accuracy, conformational ensemble size, and computing time.

Here, we present a comprehensive benchmarking study, in which we assess and compare the performance of a variety of free and commercial tools for computing conformer ensembles. Inspired by the Iridium-HT benchmarking dataset [1], we have devised a new, high-quality library of protein-bound ligand conformations from the PDB, with the aim to improve statistical significance by use of a substantially larger dataset, while adhering to strict quality criteria.

Our benchmarking tests show that several freely available conformer ensemble generators (e.g. RDKit [2], Balloon [3], CONFECT [4]) achieve a level of accuracy which is comparable to that of commercial software (e.g. MOE [5]). However, substantial differences with respect to runtimes and the percentage of molecules for which the algorithms fail to produce any solutions can be observed. Also, longer computing times do not per se lead to better conformer ensembles. From this statistical analysis we devised guidelines on how to parameterize tools for best performance in different application scenarios (e.g. computation of small data sets with maximum accuracy vs. large-scale virtual screening applications).

**References**

1. Warren GL, Do TD, Kelley BP, Nicholls A., Warren SD. Essential considerations for using protein–ligand structures in drug discovery. Drug Discov Today 2012;17:1270–81.

2. Tosco P, Stiefl N, Landrum G. Bringing the MMFF force field to the RDKit: implementation and validation. J Cheminf. 2014;6:37.

3. Vainio MJ, Johnson MS. Generating conformer ensembles using a multiobjective genetic algorithm. J Chem Inf Model. 2007;47:2462–74.

4. Schärfer C, Schulz-Gasch T, Hert J, Heinzerling L, Schulz B, Inhester T, Stahl M, Rarey M. CONFECT: Conformations from an expert collection of torsion patterns. ChemMedChem 2013;8:1690–700.

5. Molecular Operating Environment (MOE), 2013.08. Chemical Computing Group Inc., Montreal, QC.

## P7 3D-Matched Molecular Pairs: What can we use it for?

### Eugen Proschak, Julia Weber, Daniel Moser, Lena Kalinowski, Janosch Achenbach

#### Institute of Pharmaceutical Chemistry, Goethe University, Frankfurt, D-60438, Germany

##### **Correspondence:** Eugen Proschak - Proschak@pharmchem.uni-frankfurt.de

*Journal of Cheminformatics* 2016, **8(Suppl 1)**:P7

Matched Molecular Pairs is a well-established concept in Medicinal Chemistry which was successfully employed for optimization of pharmacokinetic or physicochemical properties [1]. Several recent publications from different groups suggest the extension of the MMP towards structure-based lead optimization in context of protein environment [2,3]. We introduce a strategy to relate the substitution effect within MMPs to the atom environment within the co-crystallized protein-ligand complex implemented within the VAMMPIRE database and the supplementary web interface (http://vammpire.pharmchem.uni-frankfurt.de) [4].

In this presentation we discuss different applications of the VAMMPIRE database. VAMMPIRE-LORD (lead optimization by rational design) describes an innovative strategy to improve the binding affinity of a defined lead compound using 3D-MMPs [5]. We demonstrate that the created model is able to extrapolate the knowledge of a chemical transformation and the associated effect on ligand affinity to any similar system. In a second application we discuss the use of a subset of the VAMMPIRE database for validation of scoring functions. Co-crystallized 3D-MMPs with measured affinity data are suitable for evaluation of scoring functions independent from the underlying docking algorithm. We present our findings considering the performance of scoring functions on the validation dataset derived from the VAMMPIRE database.

**References**

1. Leach AG, Jones HD, Cosgrove DA, Kenny PW, Ruston L, MacFaul P, Wood JM, Colclough N, Law B. Matched molecular pairs as a guide in the optimization of pharmaceutical properties; a study of aqueous solubility, plasma protein binding and oral exposure. J Med Chem. 2006;49:6672–82.

2. Posy SL, Claus BL, Pokross ME, Johnson SR. 3D matched pairs: integrating ligand- and structure-based knowledge for ligand design and receptor annotation. J Chem Inf Model. 2013;53:1576–88.

3. Bradley AR, Wall ID, Green DV, Deane CM, Marsden BD. OOMMPPAA: a tool to aid directed synthesis by the combined analysis of activity and structural data. J Chem Inf Model. 2014;54:2636–46.

4. Weber J, Achenbach J, Moser D, Proschak E. VAMMPIRE: a matched molecular pairs database for structure-based drug design and optimization. J Med Chem. 2013;56:5203–5207.

5. Weber J, Achenbach J, Moser D, Proschak E: VAMMPIRE-LORD: a web server for straightforward lead optimization using matched molecular pairs. J Chem Inf Model. 2015;55:207–13.

## P8 Examining the diversity of large collections of building blocks in 3D

### Mark Mackey, Tim Cheeseright

#### Cresset, Litlington, Cambridgeshire, SG8 0SS, UK

##### **Correspondence:** Mark Mackey - mark@cresset-group.com

*Journal of Cheminformatics* 2016, **8(Suppl 1)**:P8

**Fig. 4:**
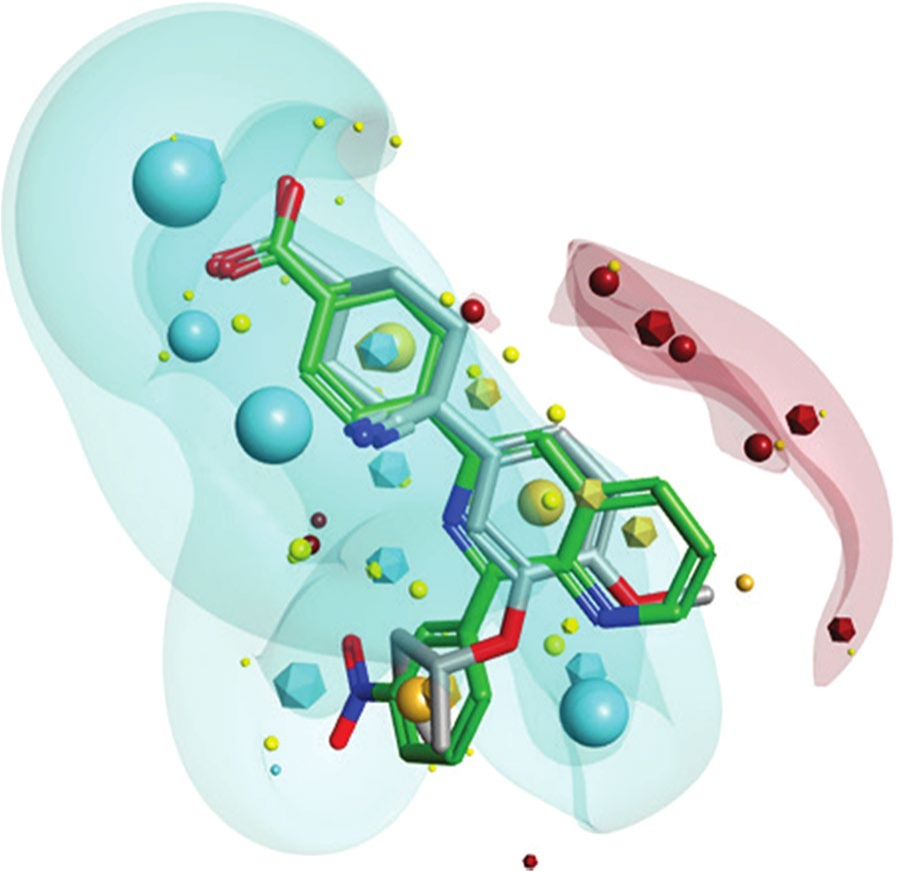
3D Cresset similarity for this pair of molecules is 0.723, whereas 2D ECFP4 fingerprint similarity is only 0.185. Electrostatic field isocontours and field point extrema are shown

One of the most widely used molecular similarity metrics to cluster large compounds sets is 2D fingerprint-based Tanimoto distance, due the overall good balance between speed and effectiveness [1]. However, there are significant limitations in the ability of a 2D fingerprint-based method to capture the biological similarity between molecules, especially when conformationally flexible structures are involved. Structures which appear to largely differ in terms of 2D structure may give rise to quite similar steric/electrostatic properties (Fig. 4), which are what actually determine their recognition by biological macromolecules [2].

We were recently confronted with the task of clustering a collection of building blocks for drug discovery consisting of about 800K heterocyclic scaffolds with variable functional group decoration. The structural diversity of this collection was not adequately captured by 2D ECFP4 fingerprint Tanimoto distances, as shown by the rather flat distribution of 2D similarity values across the set, and by their lack of correlation with the 3D similarity metrics.

The initial step of any clustering procedure is the computation of an upper triangular matrix holding similarity values between all pairs of compounds.

This step becomes computationally demanding when using 3D methods, since an optimum alignment between the molecules needs be found taking into account multiple conformers.

The talk will cover the methodological and technical solutions adopted to enable 3D clustering of such a large set of compounds. Selected examples will be presented to compare the quality and the informative content of 3D vs 2D clusters.

**References**

1. Raymond JW, Blankley CJ, Willett P: Comparison of chemical clustering methods using graph-and fingerprint-based similarity measures. J Mol Graph Model. 2003;21:421–33.

2. Cheeseright T, Mackey M, Rose S, Vinter A: Molecular field extrema as descriptors of biological activity: definition and validation. J Chem Inf Model. 2006;46:665–676.

## P9 Development of a multivariate chemometric method for the characterization of micro plastics in the marine environment

### Gerrit Renner^1,2^, Torsten C. Schmidt^2^, Jürgen Schram^1^, Marion Egelkraut-Holtus^3^, Albert van Oeyen^4^

#### ^1^Faculty of Chemistry, University of Applied Sciences Niederrhein, Krefeld, 47798, Germany; ^2^Instrumental Analytical Chemistry, University of Duisburg-Essen, Essen, 45141, Germany; ^3^Shimadzu Europa GmbH, Duisburg, 47269, Germany; ^4^CARAT GmbH, Bocholt, 46395, Germany

##### **Correspondence:** Gerrit Renner - grenner87@gmail.com

*Journal of Cheminformatics* 2016, **8(Suppl 1)**:P9

Plastics and its residues are washed into sea with severe consequences for the marine environment [1]. Due to the similar structure of some natural and synthesized polymer compounds (e.g. polyamides) it is difficult to estimate the real amount of plastic marine litter [1]. Furthermore, identification by reference spectra libraries does not succeed in combination with contaminated and / or degraded micro plastics. A new robust characterization method for high spatial resolution FTIR microscopy, using vibration band patterns to obtain specific characteristics for qualitative analysis, is introduced in the present study.

Several polymer types (PE, PP, PA, bio polymers) in different sizes (macro and micro particles) and different qualities (new, recycling, plastic marine litter) were measured in transflection mode with FTIR microscope (72 scans, R=2 cm^−1^, 5–25 repeats). Due to the practice of sampling huge amounts of plastics, no specific sample preparation is performed. The basis of the new method is the correlation between physico-chemical properties, molecular structure and patterns of vibration bands [2]. In data processing all spectra are baseline corrected and fitted with asymmetric *Voigt* functions to obtain all the vibration band areas. All areas of one spectrum are divided by each other, which leads to a highly characteristic dataset. Measurement statistics allows filtering these data to extract just analyte information and to separate matrix information. For characterization, these data are compared to references which are treated as well by multidimensional scaling combined with a novel distance function and k-Means clustering.

By this method all measured samples could be classified accurately by their similarities and differences. Even small effects as those between HD- and LD-PE are identified correctly, which can be confirmed by reference literature [3]. The new method recognizes fundamental differences of synthetic and natural polymers and classifies even unknown synthetic and bio polyamides correctly.

**References**

1. Thompson RC, et al. Lost at sea: Where is all the plastic? Science 2004;304:838.

2. Herres W, Gronholz J. Understanding FT-IR data processing. 1984;20.

3. Gulmine JV, et al. Polyethylene characterization by FTIR. Polym Test. 2002;21:557–63.

## P10 Price of reagents Vs. chemical space: Insights from the European Lead Factory Project

### Tuomo Kalliokoski

#### Lead Discovery Center GmbH, Otto-Hahn-Straße 15, 44227 Dortmund, Germany

##### **Correspondence:** Tuomo Kalliokoski - kalliokoski@lead-discovery.de

*Journal of Cheminformatics* 2016, **8(Suppl 1)**:P10

The cost of the reagents is one of the most important limiting factors in large-scale combinatorial library synthesis [1]. One task of the European Lead Factory Project (ELF) is to synthesize a new combinatorial library of 200,000 compounds for high-throughput screening purposes [2]. In this presentation, commonly used reagent classes in ELF are analyzed with respect to their price and various properties like physicochemical descriptors, 2D-chemical diversity and 3D-likeness. The effect of different price cut-offs for building blocks to the combinatorial library design is illustrated using a library from ELF as a real-life example [3].

**References**

1. Kalliokoski T. Price-focused Analysis of Commercially Available Building Blocks for Combinatorial Library Synthesis. submitted.

2. https://www.europeanleadfactory.eu

3. Craven P, Aimon A, Dow M, Fleury-Bregeot N, Guilleux R, Morgentin R, Roche D, Kalliokoski T, Foster R, Marsden SP, Nelson A. Design, synthesis and decoration of molecular scaffolds for exploitation in the production of alkaloid-like libraries. Bioorg Med Chem. 2014. doi:10.1016/j.bmc.2014.12.048.

## P11 Next-generation technologies in cheminformatics

### Denis Fourches

#### Department of Chemistry, Bioinformatics Research Center, North Carolina State University, Raleigh, North Carolina, 27695, USA

##### **Correspondence:** Denis Fourches - dfourch@ncsu.edu

*Journal of Cheminformatics* 2016, **8(Suppl 1)**:P11

Cheminformatics is becoming an essential asset in the chemist’s toolbox, especially when it comes to guiding experiments. However, the compendium of chemical biological datasets generated by experimentalists and available in publicly-accessible repositories is skyrocketing [1]. As a consequence, there is a general need for new cheminformatics technologies capable of handling, analyzing, and modeling *Big Chemical Data* [1]. In this presentation, we introduce three next-generation cheminformatics approaches that can:

(i) Enumerate extremely large virtual libraries: we will introduce the PKS Enumerator technology that generate millions of macrolides with user-defined building blocks and constraints. This research is relevant for the development of novel antibiotics.

(ii) Screen very large libraries of virtual compounds using a GPU-accelerated platform: we will show how a GPU-powered ligand-based screening workflow can perform ultra-fast similarity searches among one billion molecules.

(iii) Use machine-learning techniques for analyzing molecular dynamics trajectories: we will discuss the rationale of examining both full-atom and coarse-grained molecular dynamics trajectories using machine-learning techniques, what additional knowledge can be extracted, and how next-generation QSAR models could benefit of such technology.

**References**

1. Fourches D. Cheminformatics: at the crossroads of eras. In Gorb L et al. editors. Application of computational techniques in pharmacy and medicine. Berlin: Springer;2014. p. 539–546.

## P12 Virtual screening of Trypanosoma Inhibitors from Nigeria

### Akachukwu Ibezim^1^, Chika J. Mbah^1^, Fidele Ntie-Kang^2^, Umale M. Adikwu^3^, Ngozi J. Nwodo^1^

#### ^1^Department of Pharmaceutical and Medicinal Chemistry, Faculty of Pharmaceutical Science, University of Nigeria, Nsukka.410001 Nigeria; ^2^Department of Chemistry, Chemical and Bioactivity Information Centre, Faculty of Science, University of Buea, P.O. Box 63, Buea 00237, Cameroon; ^3^Department of Pharmaceutics, Faculty of Pharmaceutical Science, University of Nigeria, Nsukka. 410001

##### **Correspondence:** Ngozi J. Nwodo - nwodong@hotmail.com

*Journal of Cheminformatics* 2016, **8(Suppl 1)**:P12

Plant products have always been the main source of medicine for man and several works on Nigerian medicinal plants used in treatment of trypanosomiasis have been documented [1–3]. In this study, virtual screening was performed to evaluate the “drug-likeness” and ligand-receptor interaction of trypanocidal compounds isolated from Nigerian medicinal plants (Nijatryp). Lipinski’s “rule of five” (Ro5) was used to assess the relationship between structures and drug-likeness. About 94.62 % of Nijatryp compound dataset complied with Ro5 and demonstrated a significant enhancement in “drug-like” properties over Dictionary of Natural Product (DNP) dataset. About 31.18 % of plant metabolites in Nijatryp dataset have inhibition constant (K*i*) in millimolar (mM) range and four compounds, we consider as hits, have K*i* in micro-molar (µM) range. Results lay a foundation for a rationale development of new leads from Nijatryp library.

**References**

1. Atawodi SE, Bulus T, Ibrahim S, Ameh DA, Nok AJ, Mamman M, Galadima M. In vitro trypanocidal effect of methanolic extract of some Nigerian savannah plants. African J Biotech. 2003;2:317–21.

2. Adeiza AA, Maikai VA. Hassan FB. Phytochemical screening and evaluation of some medicinal plants for their in vitro activities on Trypanosoma evansi. J Med Plants Res. 2009;3:315–18.

## P13 Reaction driven molecular invention: Generating synthetically feasible design ideas

### Alexander Steudle^1^, Brian B. Masek^2^, Stephan Nagy^2^, David Baker^2^, Fred Soltanshahi^2^, Roman Dorfman^2^, Karen Dubrucq^2^

#### ^1^Certara International, Martin-Kollar-Straße 17, 81829 München, Germany; ^2^Certara, St Louis, MO 63101, United States

##### **Correspondence:** Alexander Steudle - alexander.steudle@certara.com

*Journal of Cheminformatics* 2016, **8(Suppl 1)**:P13

Reaction driven de novo design uses a library of reactions and a database of reactants to perform a stochastic walk through “synthetic chemistry space.” Guided by appropriate drug design “scoring” such as docking or ligand shape similarity, such an approach provides a means to generate novel ideas for drug candidates that includes a proposed synthesis pathway. The methodology for reaction driven molecular evolution we have developed is independent of how the design ideas are scored. We will present studies where reaction driven de novo design software simulations are used to “re-invent” know drug molecules or close analogs. When successful, the simulations also provide a proposed synthesis pathway to the compound under study. Examples which compare the “in silico” synthesis with the known synthetic path will be presented. This approach could also provide a novel means of suggesting synthesis schemes for synthetic chemists.

## P14 High-throughput docking using constraints: Development and application of a GOLD python wrapper for automated virtual screening workflows

### Hitesh Patel, Oliver Koch

#### Department of Chemistry and Chemical Biology, TU Dortmund, Germany

##### **Correspondence:** Hitesh Patel - hitesh.patel@tu-dortmund.de

*Journal of Cheminformatics* 2016, **8(Suppl 1)**:P14

Virtual screening based on high-throughput docking of millions of compounds is still not feasible due to required computational power and time. In a constrained based docking important protein-ligand interactions can be used to include additional knowledge and leading to a possible alternative for pharmacophore based pre-filtering of huge libraries. This was successfully shown by Koch et al. who identified the first inhibitors of an important protein-protein interaction in *M. tuberculosis* [1] based on a high-throughput constraint docking campaign using GOLD [2].

The underlying workflow contains several steps like constrained docking with very fast settings, pose filtering and redocking using exhaustive settings. Up to now this processing steps had to be performed manually, since GOLD lacks a programming language API. Therefore, we developed a python wrapper for processing the gold configuration file and creating python-based automated virtual screening workflows. Using this wrapper an automated constrained docking workflow was implemented and used to analyze the performance of this approach in comparison to pharmacophore mapping-based virtual screening. First studies showed promising results on Poly [ADP-ribose] polymerase 1 (PARP1), Janus Kinase 3 (JAK3) and Phosphoinositid-3-Kinase gamma (PI3Kg) from the DEKOIS2.0 dataset [3].

**References**

1. Koch O, et al. Identification of M. tuberculosis thioredoxin reductase inhibitors based on high-throughput docking using constraints. J Med Chem. 2013;56:4849–59.

2. Jones G, Willett P, Glen RC. Molecular recognition of receptor sites using a genetic algorithm with a description of desolvation. J Mol Biol. 1995;245:43–53.

3. Bauer MR, et al. Evaluation and optimization of virtual screening workflows with DEKOIS 2.0—a public library of challenging docking benchmark sets. J Chem Inf Model. 2013;53:1447–62.

## P15 Computation and optimization of complex formation thermodynamics by a solute-solute integral equation theory

### Florian Mrugalla, Stefan M. Kast

#### Physikalische Chemie III, TU Dortmund, 44227 Dortmund, Germany

##### **Correspondence:** Stefan M. Kast - stefan.kast@tu-dortmund.de

*Journal of Cheminformatics* 2016, **8(Suppl 1)**:P15

The development of novel drugs is an arduous procedure which can take more than a decade and can accumulate expenses in the triple digit million euro range [1]. These facts remain true in spite of recent advances in virtual screening and computer-aided drug design [2]. The need for reasonably accurate and fast methods to predict and optimize the binding affinity which can drive the design process of drug-like molecules is therefore very high.

We here present novel methodology for computing binding free energies and their analytical dependence on chemical properties based on the so-called solute-solute equation of the reference interaction site model (RISM) [3]. This integral equation theory is derived from the molecular Ornstein-Zernicke equation and enables us to calculate the potential of mean force (PMF) including physics-based solvation contributions with very high computational efficiency. The PMF is the key quantity for characterizing chemical and biological processes since it represents the free energy change along a given reaction coordinate from which the binding free energy can be computed.

We derive the conceptual framework and demonstrate various application areas of the model. PMF results from the solute-solute RISM approach are compared with explicit-solvent free energy perturbation molecular dynamics simulations in order to assess the approximations inherent to the theory for simple model systems. Optimal binding chemistry is defined by minimizing the free energy with respect to nonbonded complex interaction parameters. The results lead to suggestions for novel approaches to define protein-ligand scoring functions.

**References**

1. Lavecchia A, Di Giovanni C. Virtual screening strategies in drug discovery: a critical review. Curr Med Chem Phys. 2014;20:2839–60.

2. Michel J. Current and emerging opportunities for molecular simulations in structure-based drug design. Phys Chem Chem Phys. 2014;16:4465.

3. Kloss T, Kast SM. Treatment of charged solutes in three-dimensional integral equation theory. J Chem Phys. 2008;128:134505.

## P16 Understanding the functional efficacy profiles of GABA α-1/α-2 selective modulators

### Qurrat U Ain^1^, Julian E. Fuchs^1^, Robert M. Owen^2^, Kiyoyuki Omoto^2^, Rubben Torella^2^, David C. Pryde^2^, Robert Glen^1^, Andreas Bender^1^

#### ^1^Centre for Molecular Informatics, Department of Chemistry, University of Cambridge, Lensfield Road, Cambridge, CB2 1EW, United Kingdom; ^2^Worldwide Medicinal Chemistry, Pfizer Neusentis, The Portway Building, Granta Park, Great Abington, Cambridge, Cb21 6GS, United Kingdom

##### **Correspondence:** Andreas Bender - ab454@cam.ac.uk

*Journal of Cheminformatics* 2016, **8(Suppl 1)**:P16

The emerging understanding of selective modulation of Gamma Amino Butyric Acid (GABA) receptors allows an increase in the development of clinically efficacious and subtype selective drugs for these targets [1]. The development of various α-2/3 selective agonists is in process, for the treatment of anxiety disorders [2,3], however, the binding modes of these selective modulators is still unknown. In our study, we propose a unified pipeline of chemo genomic analyses of GABA modulators and established a link between their bioactivity, efficacy and mode of action on α-1 and α-2 receptors. We applied this protocol on a dataset of 5,440 GABA modulators and extracted a subset of compounds with desired structure activity relationship (SAR) and structure efficacy relationship (SER). The similar behavior of candidate compounds in structure and activity space and their opposite behavior in functional efficacy space was assessed by assay related target similarity (ARTS) method. The dynamical behavior of GABA α-1/2 in the presence of selected compounds was analyzed using molecular dynamics simulations to further elucidate the binding modes. The results obtained showed that sequence differences (G200E, I201T, V202I) in the loop C region of GABA α-2 subunit are likely to be responsible for destabilizing the active site and hence decreasing the rigidity in presence of modulators as compared to α-1. To summarize, the behavior of the active site in presence of different modulators suggests that the difference in binding conformation of compounds could be attributed to structural differences in either the protein subunits or the compounds, and hence could possibly be a reason for their selective functional efficacy profiles.

**References**

1. Bagal SK, Brown AD, Cox PJ, Omoto K, Owen RM, Pryde DC, Sidders B, Skerratt SE, Stevens EB, Storer RI, Swain NA. Ion channels as therapeutic targets: a drug discovery perspective. J Med Chem. 2013;56:593–624.

2. Atack JR: GABAA receptor subtype-selective modulators. I. α2/α3-selective agonists as non-sedating anxiolytics. Curr Top Med Chem. 2011;11:1176–202.

3. Chen X, de Haas S, de Kam M, van Gerven J: An overview of the CNS-pharmacodynamic profiles of nonselective and selective GABA agonists. Adv Pharmacol Sci. 2012;134523.

## P17 Metadynamics viewer Metadyn View

### Petr Hošek, Vojtěch Spiwok

#### Department of Biochemistry, University of Chemistry and Technology, Prague, Technická 3, Prague 6, 166 28, Czech Republic

##### **Correspondence:** Vojtěch Spiwok - spiwokv@vscht.cz

*Journal of Cheminformatics* 2016, **8(Suppl 1)**:P17

Here we demonstrate a new tool Metadyn View for visualisation of results of metadynamics [1] simulation technique. Metadynamics enhances sampling of classical molecular dynamics simulation by use of a bias potential. This bias potential can be used to predict the free energy surfaces of the studied system, i.e. equilibria and kinetics of key conformational changes, binding events etc.

Metadyn view [2] is a web-based program for exploration and analysis of free energy surfaces calculated by metadynamics. A user can load the results from metadynamics (a HILLS file), visualise the free energy at different stages of simulation, measure free energy differences and locate representative snapshot for free energy minima. It can be accessed on-line at http://metadyn.vscht.cz.

Applications of this tool to new simulations will be presented.

**Acknowledgements:** Ministry of Education, Youth and Sports (COST action GLISTEN, CM1207, LD14133) and Czech Science Foundation (15-17269S).

**References**

1. Laio A, Parrinello M. Escaping free energy minima. Proc Natl Acad Sci 2002;99:12562–6.

2. Hošek P, Spiwok V. Metadyn view: fast web-based viewer of free energy surfaces calculated by metadynamics. Comput Phys Comm. accepted.

## P18 Application of an *in silico* Mechanism-of-Action protocol to high-Content cytotoxicity screening data

### Lewis H. Mervin^1^, Ian Barrett^2^, Mike Firth^2^, David C. Murray^3^, Lisa McWilliams^3^, Qing Cao^4^, Ola Engkvist^5^, Andreas Bender^1^

#### ^1^Centre for Molecular Informatics, Department of Chemistry, University of Cambridge, UK; ^2^Discovery Sciences, AstraZeneca R&D Cambridge, Cambridge Science Park, UK; ^3^Discovery Sciences, AstraZeneca R&D Alderley Park, Alderley Park, UK; ^4^Discovery Sciences, AstraZeneca R&D Boston, USA; ^5^Chemistry Innovation Centre, AstraZeneca R&D Mölndal, Sweden

##### **Correspondence:** Andreas Bender

*Journal of Cheminformatics* 2016, **8(Suppl 1)**:P18

*In silico* Mechanism-of-Action (MoA) analysis protocols have been developed in recent years, comprising molecule bioactivity profiling, annotation of predicted targets with pathways and calculation of enrichment factors to highlight targets and pathways likely to be implicated in the studied phenotype [1,2]. Although such analyses report that pathway annotation improves the MoA information gained than by target prediction alone, these experiments are conducted on small and inconsistent datasets (approximately 1,500 compounds[1]).

In this work, we have combined an *in silico* target prediction tool, utilising over 9.5 million and 600 million active and inactive datapoints respectively, with automated pathways annotation from the NCBI BioSystems database. This MoA protocol has been applied to over 6,800 cytotoxic and 300,000 non-cytotoxic compounds from AstraZeneca cell viability screens in order to rationalise the cytotoxic MoA. Post-processing of predictions involved the removal of targets with correlated activity due to overlapping bioactive compound annotations. Many of the enriched targets support known mechanisms of cytotoxicity, with multiple pathways clustering around processes responsible for the fidelity of cell cycle, cellular division and metabolism.

**References**

1. Liggi S, et al. Extending in silico mechanism-of-action analysis by annotating targets with pathways: application to cellular cytotoxicity readouts. Fut Med Chem. 2014;6:2029–56.

2. Liggi S, et al. Extensions to in silico bioactivity predictions using pathway annotations and differential pharmacology analysis: application to xenopus laevis phenotypic readouts. Mol Inf. 2013;32:1009–24.

## P19 Mean Information Content (MIC) algorithm: a new approach for fingerprint hybridization and reduction

### Dawid Warszycki^1^, Marek Śmieja^2^, Andrzej J. Bojarski^1^

#### ^1^Institute of Pharmacology Polish Academy of Sciences, Krakow, 31-343, Poland; ^2^Faculty of Mathematics and Computer Science Jagiellonian University, Krakow, 30-348, Poland

##### **Correspondence:** Dawid Warszycki - warszyc@if-pan.krakow.pl

*Journal of Cheminformatics* 2016, **8(Suppl 1)**:P19

Fingerprints, a bit representation of compound chemical structure, have been widely used in cheminformatics for many years. The conversion of chemical structures into the bit strings is based on either structural keys or graph representations. Despite the fact that fingerprints with the highest resolution display high performance in virtual screening campaigns [1], the presence of a relatively high number of irrelevant bits introduces noise and makes their application more time-consuming.

Here, we present a new method of hybrid reduced fingerprints construction—the MIC algorithm. The methodology was applied for fingerprints implemented in the PaDEL-Descriptor software [2]. The MIC algorithm was applied for ligands of cognate serotonin receptors (5-HT_2A_R, 5-HT_2B_R, 5-HT_2C_R, 5-HT_5A_R and 5-HT_6_R). In the study, the length and composition of fingerprint is optimized separately for every single receptor by an algorithm which iteratively maximizes the amount of information included. Generated fingerprints applied in random forest experiment outperformed every raw (original) fingerprint. Moreover, a universal fingerprint for the whole set of receptors was also developed and applied in machine learning experiments carried out on 5-HT_1A_ receptor ligands, which gave satisfactory results. The composition of hybrid fingerprints highlights the most important structural features of the serotonin receptors’ ligands in machine learning evaluation.

**Acknowledgement:** This study was partially supported by the National Centre of Science from Poland (grant no. 2014/13/N/ST6/01832).

**References**

1. Sastry M, Lowrie JF, Dixon SL, Sherman W. Large-scale systematic analysis of 2D fingerprint methods and parameters to improve virtual screening enrichments. J Chem Inf Model. 2010;50:771–84.

2. He YY, Liew CY, Sharma N, Woo SK, Chau YT, Yap CW. PaDEL-DDPredictor: open-source software for PD-PK-T prediction. J Comput Chem. 2013;34:604–10.

## P20 Prediction of various ATP-binding cassette substrates via decision trees and multi-label classification

### Natalia Aniceto^1^, Andreas Bender^2^, Alex Freitas^3^, Taravat Ghafourian^1^

#### ^1^Medway School of Pharmacy, Universities of Kent and Greenwich, Kent ME4 4TB, UK;^2^Centre for Molecular Science Informatics, Department of Chemistry, University of Cambridge, Cambridge CB2 1EW, UK; ^3^School of Computing, University of Kent, Canterbury, Kent CT2 7NF, UK

##### **Correspondence:** Taravat Ghafourian - t.ghafourian@kent.ac.uk

*Journal of Cheminformatics* 2016, **8(Suppl 1)**:P20

The pharmacokinetic profile is paramount for the successful development of a new drug, and pharmacokinetics issues have a big impact on late stage attrition [1]. The ABC transporters’ efflux is one of the main factors controlling the pharmacokinetics of a drug, hence being heavily studied. Here, we present two decision tree-based, multi-label QSAR models able to simultaneously predict substrates and non-substrates in a total of 1493 compounds for 4 of the main ABC transporters [2] (BCRP1, MDR1, MRP1 and MRP2). Each of these transporters corresponds to a binary class variable, taking the value (class label) “substrate” or “non-substrate”, and the simultaneous prediction of the four class variables is performed by a multi-label classification model, unlike the conventional (single-label) classification task where just one class variable is predicted. One of the models—a Classifier Chain - was built to consider transporter (class) interaction, and a Binary Relevance model was used as the baseline model. Training, optimization and testing were performed with separate portions of the dataset. Feature selection was optimized from 5 different methods where the best method chosen for each transporter. Both models were validated with testing against y-randomization. The applicability domain was characterized using the STD method [3], and activity cliffs and chemical space coverage were determined. Both multi-label models yielded 70% accuracy in the test set, but considering label interaction lead to more balanced models. The AD was able to reliably map the predictive accuracies across the dataset, which means that predictive reliability of new queries can be correctly determined. Moreover, various mispredictions coincided with activity cliff areas, which means that they could not have been picked up as outliers by any model using similar molecular descriptors. This multi-label classification approach is an appropriate alternative for addressing the prediction of ABC efflux, especially considering the unspecific nature of substrate recognition by the ABC family members.

**References**

1. Demel MA, Kraemer O, et al. Ensemble rule-based classification of substrates of the human ABC-transporter ABCB1 using simple physicochemical descriptors. Mol Inf. 2010;29:233–42.

2. Pinto M, Digles D, Ecker GF. Computational models for predicting the interaction with ABC transporters. Drug Discov Today Technol. 2014;12:e69–77.

3. Tetko IV, Sushko I, et al. Critical assessment of QSAR Models of environmental toxicity against tetrahymena pyriformis: focusing on applicability domain and overfitting by variable selection. J Chem Inf Model. 2008;48:1733–46.

## P21 Integration of chemical structure and full-text search: MarkLogic goes chemistry

### Guido Herrmann^1^, Valentina Eigner-Pitto^2^

#### ^1^Georg Thieme Verlag KG, Stuttgart, 70469, Germany; ^2^InfoChem GmbH, München, 81241, Germany

##### **Correspondence:** Valentina Eigner-Pitto - ve@infochem.de

*Journal of Cheminformatics* 2016, **8(Suppl 1)**:P21

The pharmaceutical and health industry makes continuous efforts to improve and increase the available variety of pharmaceutical treatments. Medicinal chemists start the innovation cycle by systematic searches in all available published information, i.e. scientific journals, chemical databases and internal documentations.

A challenge is the existence of two separate information repositories: full text information and chemical structures/reactions contained in databases. This talk will describe how InfoChem has developed a technological infrastructure within the MarkLogic platform that allows effective and integrated searches in both full-text and chemical information.

Our talk will describe the underlying technologies developed in the ongoing project and present results of combined full-text and structure/reaction searches.

## P22 A new method for binding site shape clustering to increase selectivity of PTP1B ligands

### Alexandra Naß, Gerhard Wolber

#### Institut für Pharmazie, Freie Universität Berlin, 14195 Berlin, Deutschland

##### **Correspondence:** Alexandra Naß - alexandra.nass@fu-berlin.de

*Journal of Cheminformatics* 2016, **8(Suppl 1)**:P22

Since available crystal structures usually only cover a small portion of possible protein conformations, drug development processes based on them can only detect a limited number of all possible ligands. However, ligand-based approaches often cover a broader range of binding conformations, but only a small part of possible binding site space at the protein, which is a reason to prefer structure based methods. Additionally, proteins with very similar binding site in available crystal structures can still adapt other conformations of high probability which enable the design of selective ligands.

In order to close this gap in ligand design a new method for binding site shape clustering was developed based on the test case PTP1B/TC-PTP. PTP1B is a long known validated target for type 2 diabetes and obesity, while inhibition of the closely related TC-PTP leads to intolerable side effects in mice [1].

To collect protein conformations, molecular dynamics simulations were performed. Since for TC-PTP no crystal structure with required starting conformation was available, a homology model was used instead. The collected molecular dynamics frames were aligned and for each frame the binding site was filled with a grid of points using the software POVME2 [2]. The binding site shape information derived from the grids was translated into a matrix containing binary data for presence or absence of each point inside the binding site of the corresponding frame. Different clustering algorithms were then used to extract the present information on binding site shape differences. This way, binding site shapes with high probability in PTP1B, but low probability in TC-PTP can be discovered and the evidence can be used to select favourable shapes and interaction points for selective PTP1B inhibitors.

**References**

1. Bialy L, Waldmann H. Inhibitors of protein tyrosine phosphatases: next-generation drugs? Angew Chem Int Ed. 2005;44:3814–39.

2. Durrant JD, Votapka L, Sørensen J, Amaro RE: POVME 2.0: an enhanced tool for determining pocket shape and volume characteristics. J Chem Theory Comput. 2014;10:5047–56.

## P23 New approach for evaluation of docking results based on hybrid interaction fingerprint and machine learning methods

### Rafał Kurczab, Andrzej J. Bojarski

#### Department of Medicinal Chemistry, Institute of Pharmacology Polish Academy of Sciences, 12 Smetna Street, 31-343 Cracow, Poland

##### **Correspondence:** Rafał Kurczab - kurczab@if-pan.krakow.pl

*Journal of Cheminformatics* 2016, **8(Suppl 1)**:P23

Molecular docking is a one of the key tools in computer-aided drug design that is mainly used in virtual screening of compound libraries and to study mechanism of ligand-target interaction. However, a problem of effective scoring and discrimination between active and inactive compounds is still not completely solved [1].

In this study, a novel protocol for the automatic evaluation of docking results is presented. In the first stage, the known active and inactive compounds are docked to receptor binding site; then the molecular interactions from the resulting ligand-protein complexes are encoded into binary format by means of the SIFt algorithm [2]. In parallel, conformations of docked ligands are used to calculate 3D molecular descriptors (PaDEL software [3]), and further concatenated with SIFt vectors. The obtained hybrid fingerprints are used as an input for the selected machine learning methods (e.g. SVM, RF, J48, Naïve Bayes) implemented in WEKA software [4].

Evaluation using compounds active toward 5-HT_6_, 5-HT_7_ and 5-HT_1B_ receptors, proved that the methodology can be successfully used for supporting virtual screening protocols.

**Acknowledgments:** The study was partially supported by the Polish-Norwegian Research Programme operated by the National Centre for Research and Development under the Norwegian Financial Mechanism 2009–2014 in the frame of Project PLATFORMex (Pol-Nor/198887/73/2013) and by the National Science Center Grant No DEC-2012/05/B/N27/03076.

**References**

1. Meng XY, Zhang HX, Mezei M, Cui M. Molecular docking: a powerful approach for structure-based drug discovery. Curr Comput Aided. Drug Des. 2011;7:146–57.

2. Mordalski S, Kosciolek T, Kristiansen K, Sylte I, Bojarski AJ. Protein binding site analysis by means of structural interaction fingerprint patterns. Bioorg Med Chem Lett. 2011;21:6816–19.

3. Yap, CW. PaDEL-descriptor: an open source software to calculate molecular descriptors and fingerprints. J Comput Chem. 2011;32:1466–74.

4. Hall M, Frank E, Holmes G, Pfahringer B, Reutemann P, Witten IH. The WEKA data mining software: an update. SIGKDD Explorations 2009;11:1.

## P24 Lessons learned from applying halogen bonding in molecular design—the c-Jun N-terminal kinase 3 (JNK3) as target for sulfur…halogen bonds

### Andreas Lange^1^, Marcel B. Günther^2^, Markus O. Zimmermann^1^, Susanne Hennig^1^, Felix M. Büttner^3^, Christoph Schall^3^, Adrian Sievers-Engler^4^, Francesco Ansideri^2^, Pierre Koch^2^, Thilo Stehle^3^, Stefan Laufer^2^, Frank M. Böckler^1^

#### ^1^Departement of Pharmaceutical Science, Mol. Design, Eberhard Karls University Tuebingen, Auf der Morgenstelle 8, 72076 Tuebingen, Germany; ^2^Departement of Pharmaceutical Science, Medicinal Chemistry, Eberhard Karls University Tuebingen, Auf der Morgenstelle 8, 72076 Tuebingen, Germany; ^3^Interfaculty Institute of Biochemistry, Eberhard Karls University Tuebingen, Hoppe-Seyler-Str. 4, 72076 Tuebingen, Germany; ^4^Departement of Pharmaceutical Analysis and Bioanalysis, Eberhard Karls University Tuebingen, Auf der Morgenstelle 8, 72076 Tuebingen, Germany

##### **Correspondence:** Andreas Lange - andreas.lange@uni-tuebingen.de

*Journal of Cheminformatics* 2016, **8(Suppl 1)**:P24

For a broader recognition of halogen bonding in molecular design, we have recently studied halogen bonding contacts with different interaction partners in protein binding sites [1–4]. One of our goals was to examine the accessibility of the sulfur atom in the amino acid methionine through halogen bonding [1]. We found in the c-Jun N-terminal kinases 3 (JNK3) an interesting target. In the binding pocket of JNK3 the sulfur of methionine 146 (MET146) appears to be targetable by halogen bonding (PDB 2p33) [5]. Herein, a chlorine atom shows very favourable distance (336 pm) and σ–hole angle (160.4°) to the sulfur of methionine. In panel a) of the picture below, spherical interaction energies of iodine toward sulfur are plotted onto MET146. Favourable interaction geometries are highlighted by red colouring. The placement and bond direction of chlorine in the depicted ligand suggests a reasonable good quality of halogen bond (yellow area). DFT-calculations of a meaningful part of the binding pocket (TPSS-D/SVP) indicated that an exchange of chlorine by bromine and iodine could be useful for the strength of the halogen bond (b+c). Individual synthetic access was developed for the heavier halide analogues, which were then characterized by various biophysical techniques (d+e). By showing the crystal structure of the iodine analogue in complex with JNK3, we have obtained very valuable insights into the possibility and limitations of the application of halogen bonding in molecular design, which will be communicated in this talk [6].

**References**

1. Wilcken, et al. J Chem Theory Comput. 2011;7:2307–15.

2. Wilcken, et al. J. Comput. Aided Mol. Des. 2012;26:935–45.

3. Lange, et al. J Chem Inf Model. 2013;53:3178–89.

4. Wilcken, et al. J Med Chem. 2013;56:1363–88.

5. Alam, et al. Bioorg. Med. Chem. Lett. 2007;17:3463–67.

6. Lange, et al. submitted.

## P25 Combining various open data sources for P-gp/BCRP selectivity profiling

### Barbara Zdrazil, Floriane Montanari, Gerhard F. Ecker

#### University of Vienna, Department of Pharmaceutical Chemistry, Division of Drug Design and Medicinal Chemistry, Pharmacoinformatics Research Group, Althanstraße 14, Vienna, A-1090, Austria

##### **Correspondence:** Barbara Zdrazil - barbara.zdrazil@univie.ac.at

*Journal of Cheminformatics* 2016, **8(Suppl 1)**:P25

It is widely accepted that the human ATP binding cassette transporters P-glycoprotein (P-gp, MDR1, ABCB1) and breast cancer resistance protein (BCRP, ABCG2) are working in concert in order to restrict brain penetration of certain drugs. Known substrates and inhibitors of these transporters are showing a great structural variety, however, there exists some overlap in compound selectivity between the two. Because dual inhibition of P-gp/BCRP would lead to better oral bioavailability and CNS penetration of i.e. anticancer drugs, information on determinants for specific or dual inhibition would be of great value in early phases of drug discovery.

However, the collection of pharmacological data for a target of interest is a tedious task, especially if data from open data sources (like Open PHACTS or ChEMBL) is combined with literature data. If there are multiple targets involved, then the work becomes even harder.

We tackled this research question by creating a KNIME [1] workflow for conveniently combining data from the Open PHACTS Discovery Platform [2] and other data sources, including pre-processing, filtering, annotation (e.g. creating a binary representation of the bioactivities), and visualization steps. In our use case, P-gp and BCRP inhibition data was collected and their compound overlap was determined. Different ways of solving multi-label problems were explored and compared: label-powerset, binary relevance and classifiers chain. Label-powerset revealed important molecular features for selective or polyspecific inhibitory activity, while binary relevance and classifiers chain allow for more predictive models. The models reveal important molecular features for specific or polyspecific inhibitory activity such as SlogP, the number of donors and acceptors of H bonds, the number of aromatic atoms and the length of the maximum single bond chain.

**Acknowledgements:** We acknowledge financial support provided by the Austrian Science Fund (FWF), Grant F03502 and from a ‘Back to Research Grant’ funded by the Faculty of Life Sciences, University of Vienna. We also acknowledge support from the Innovative Medicines Initiative Joint Undertaking under grant agreement no. [115191], resources of which are composed of financial contribution from the European Union’s Seventh Framework Programme (FP7/2007–2013) and in-kind contribution of EFPIA companies.

**References**

1. https://www.knime.org.

2. https://www.openphacts.org.

## P26 Are elastic network models enough? Integration of experimentally derived driving modes in ligand-protein sampling

### Christoph Grebner^1^, Anders Hogner^1^, Johan Ulander^1^, Karl Edman^3^, Victor Guallar^4,5^, Christian Tyrchan^2^

#### ^1^CVMD, AstraZeneca, Mölndal, Sweden; ^2^RIA, AstraZeneca, Mölndal, Sweden; ^3^Discovery Sciences, AstraZeneca, Mölndal, Sweden; ^4^Joint BSC-IRB Research Program in Computational Biology, BSC, Barcelona, Spain; ^5^Institució Catalana de Recerca i Estudis Avançats (ICREA), Barcelona, Spain

##### **Correspondence:** Christoph Grebner - christoph.grebner@astrazeneca.com

*Journal of Cheminformatics* 2016, **8(Suppl 1)**:P26

Understanding the underlying processes upon ligand binding is a key parameter in drug design. The early view of a lock-and-key principle has evolved to a more dynamic view of protein-ligand interactions. This requires an accurate treatment of both, the ligand and the protein dynamics. Up to now, there are still no robust methods to describe the complex interplay between ligands and proteins in an efficient manner and often these methods are limited to highly specialized hardware.

Recently, a novel Monte Carlo based algorithm combined with side chain prediction was introduced which efficiently explores protein-ligand and protein-protein dynamics [1]. This Protein Energy Landscape Exploration (PELE) method has provided efficient and accurate induced fit results in respect to ligand migration and ligand binding events [2–4]. Originally, the protein backbone motion is limited to normal modes obtained by the *anisotropic network model* (ANM), a pure theoretical approach based on a distance matrix. Experimental parameters are not directly incorporated in these simulation, despite the fact that experimental data derived from X-Ray or NMR contain valuable information regarding conformational space and protein dynamics.

To make use of public or in-house available experimental information, we incorporated driving modes based on X-Ray structures. Testing the new approach using the mineralocorticoid receptor system showed an improvement of the general performance and the description of the protein motion. Currently, we are implementing NMR chemical shift information into PELE which guides the protein motion following experimentally observed chemical shifts. Overall, this will allow for efficient sampling of ligand-protein binding events under direct consideration of experimental data.

**References**

1. Borrelli KW, Vitalis A, Alcantara R, Guallar V. PELE: protein energy landscape exploration. A novel Monte Carlo based technique. J Chem Theory Comp. 2005;6:1304–11.

2. Cossins BP, Hosseini A, Guallar V. Exploration of protein conformational change with PELE and meta-dynamics. J Chem Theory Comput 2012;8:959–65.

3. Borrelli KW, Cossins B, Guallar V. Exploring hierarchical refinement techniques for induced fit docking with protein and ligand flexibility. J Comp Chem. 2010;31:1224–35.

4. Edman K, Hogner A, Husseini A, Bjursell MK, Aagaard A, Bäckström S, Bodin C, Wissler L, Jellesmark-Jensen T, Cavallin A, Karlsson U, Nilsson E, Lecina D, Takahashi R, Grebner C, Lepistö M, Guallar V. Ligand recognition in steroid hormone receptors: from conserved plasticity to binding mechanism. To be published.

## P27 How certain are you? Incorporating assay variability in the decision process

### Johan Ulander^1^, Christian Tyrchan^2^, Wolfgang Klute^3^, Fredrik Bergström^1^, Christian Kramer^4^

#### ^1^AstraZeneca, CVMD iMED, Mölndal, Sweden; ^2^RIA iMED, Mölndal, Sweden; ^3^RDI, Mölndal, Sweden; ^4^F. Hoffmann-La Roche, Pharma Early Research and Development, Basel, Switzerland

##### **Correspondence:** Johan Ulander - johan.ulander@astrazeneca.com

*Journal of Cheminformatics* 2016, **8(Suppl 1)**:P27

We have made a comprehensive analysis of all IC50 and EC50 values with existing target gene id from the internal AstraZeneca screening and test database IBIS. The data was analyzed to arrive at statistically well-founded estimates for intra laboratory assay reproducibility. We find that the typical experimental precision is higher than commonly assumed with typical standard deviations between 0.21 and 0.26 log units. Interestingly the experimental uncertainty is relatively independent of the test technology target-class and the functional read-out (e.g. IC50 or EC50). Further we do not observe strong correlations of variability with common physico-chemical properties like logD or solubility. We also provide some explicit examples of ways to deal with uncertainty in data [1,2].

**References**

1. Kramer C, Kalliokoski T, Gedeck P, Vulpetti A. The experimental uncertainty of heterogeneous public ki data. J Med Chem. 2012;55:5165–73.

2. Wan H, Bold P, Larsson L,.Ulander J, Peters S, Löfberg B, Ungell A, Någård M, Llinas A. Impact of input parameters on the prediction of hepatic plasma clearance using the well-stirred model. Curr Drug Metab. 2010;11:583–94.

## P28 Application of novel machine learning approaches for scoring and ranking of docking poses

### Quoc Dat Nguyen, Wolfgang Sippl

#### Institute of Pharmacy, University of Halle, Halle (Saale), 06120 Germany

##### **Correspondence:** Quoc Dat Nguyen - dat.nguyen@pharmazie.uni-halle.de

*Journal of Cheminformatics* 2016, **8(Suppl 1)**:P28

Molecular docking is the most widely used method in modern computer-aided drug design, regardless of being used in virtual screen or lead optimization. Molecular docking predicts the preferred orientation of one molecule (ligand) to a bigger molecule (protein) to form a stable complex. The most complex and important step in this docking process is scoring. In this process, a large number of binding poses are computationally generated and then evaluated using a scoring function (SF), which is a mathematical or predictive model that produces a score representing binding stability of the pose. Generally, three main aspects of a SF define its goodness, they are “docking power”, “ranking power” and “scoring power” [1]. Reportedly, conventional SFs are able to predict binding modes while mostly failed to predict binding affinities. In literature, SFs are typically classified as force-field-based, empirical, and knowledge-based. A first application of machine learning using Random Forest to predict binding affinities shows an increasing of more than 20% in term of Pearson’s correlation coefficient in a generic benchmark set with 195 protein-ligand complexes [2]. Since then, a new class of SF has been intensively studied, the machine learning-based SF. Using CASF databases [3], we evaluate 11 novel conventional SFs (e.g. goldscore, DrugScore) and 10 machine learning-based SFs, we could show that scoring and ranking power of machine learning-based SFs are superior in comparison to conventional SFs. Especially ensemble learning classifier like Rotation Forest, in combination with diverse modifications by our own to guarantee diversity and accuracy, achieves the best performance in all cases.

**References**

1. Cheng T. Comparative assessment of scoring functions on a diverse test set. J Chem Inf Comput Model. 2009;49:1079–93.

2. Ballester PJ. A machine learning approach to predicting protein-ligand binding affinity with applications to molecular docking. Bioinformatics 2010;26:1169–75.

3. Li Y, Han L, Liu Z, Wang R. Comparative assessment of scoring functions on an updated benchmark. J Chem Inf Comput Model. 2014;54:1717–36.

## P29 Electronic structure at high solvent pressure

### Roland Frach^1^, Patrick Kibies^1^, Steven Strohfeldt^1^, Saraphina Böttcher^1^, Tim Pongratz^1^, Dominik Horinek^2^, Stefan M. Kast^1^

#### ^1^Physikalische Chemie III, TU Dortmund, 44227 Dortmund, Germany; ^2^Institut für Physikalische und Theoretische Chemie, Universität Regensburg, 93040 Regensburg, Germany

##### **Correspondence:** Stefan M. Kast - stefan.kast@tu-dortmund.de

*Journal of Cheminformatics* 2016, **8(Suppl 1)**:P29

While the majority of life on earth is adapted to ambient pressure conditions, on aver-age about 88% of the oceanic water on earth is 3800 m deep, corresponding to a hydrostatic pressure of 380 bar, which is by far not the highest value on earth [1]. Bio-chemical processes for the vast number of lifeforms accommodated to these extreme conditions are barely understood.

Applying high solvent pressure to biomolecules has substantial impact on their free energy surfaces that govern structure, function and dynamics. This poses a challenge to computational modeling approaches since the applicability of conventional empirical force fields is not known. As a step toward clarifying the situation, we need to ac-count for high pressure in quantum-chemical calculations.

A suitable methodology is provided by integral equation theories, in particular the “embedded cluster reference interaction site model” (EC-RISM) [2,3] that combines statistical-mechanical 3D RISM integral equation theory and quantum-chemical calculations self-consistently. In this context the impact of pressure is naturally accounted for since the solvent susceptibility function that enters the theory contains the pure solvent correlation functions at the pressure chosen. Here we illustrate the methodology for several benchmark applications in a pressure range of 1 bar up to 10 kbar, including the effect of pressure on molecular structure, the relevance of electronic polarizability un-der extreme conditions, and the pressure dependence of nuclear magnetic resonance parameters.

**References**

1. Daniel I, Oger P, Winter R. Origins of life and biochemistry under high-pressure conditions. Chem Soc Rev 2006;35:858–75.

2. Kloss T, Heil, J, Kast SM. Quantum chemistry in solution by combining 3D integral equation theory with a cluster embedding approach. J Phys Chem B 2008;112:4337–43.

3. Frach R, Kast SM. Solvation effects on chemical shifts by embedded cluster integral equation theory. J Phys Chem A 2014;118:11620–8.

## P30 DACS: Chronological database development of the commercially available chemical space

### Bernd Rupp, Raed Al-Yamori, Michael Lisurek, Ronald Kühne

#### Structural Biology, AG Computational Chemistry/Drug Design, Leibniz-Institut für Molekulare Pharmakologie (FMP), Berlin, D-13125, Germany

##### **Correspondence:** Bernd Rupp - rupp@fmp-berlin.de

*Journal of Cheminformatics* 2016, **8(Suppl 1)**:P30

DACS (Database of Available Chemical Compounds) is a virtual compound collection of chemical substances prepared for analysis of the chemical space at the FMP. The database contains compounds offered since 2005 by more than 50 different vendors. Up to now we collected altogether 375 million data records, which were extracted from nearly 3000 files. These data records represent more than 80 million unique chemical structures.

In July 2015, the beta release of open-dacs.de combined with an active web-interface is available for users. This release contains more than 19 million unique structures. In the next release we are planning to include the 3D representations of the unique compounds in complementary to the 2D-structures to facilitate the direct application for virtual screening and docking studies.

Furthermore, a first standard set of descriptors, such as number of H-bond donors/acceptors (NHBA/D), psa, alogp and divers solubility models [1–3] is available for filtering the search results. All chemical structures are tagged according to their content of reactive substructures following a list of SMART based rules, which are developed in the context of several library design initiatives [4, 5].

**References**

1. Wichard J, Kuehne R: Predicting aqueous solubility from structure. J Univ Appl Sci Mittweida. In Proceedings of the 20th IWKM, 28–29, 2009.

2. Cheng A, Merz K Jr. Prediction of aqueous solubility of a diverse set of compounds using quantitative structure-property relationships. J Med Chem. 2003;46:3572–80.

3. Tetko I, Tanchuk V, Kasheva T, Villa A: Estimation of aqueous solubility of chemical compounds using E-state indices. J Chem Inf Comput Sci. 2001;41:1488–93.

4. Lisurek M, Rupp B, Wichard J, Neuenschwander M, von Kries JP, Frank R, Rademann J, Kühne R. Design of chemical libraries with potentially bioactive molecules applying a maximum common substructure concept. Mol Divers. 2010;14:401–8.

5. Horvath D, Lisurek M, Rupp B, Kühne R, Specker E, von Kries JP, Rognan D, Andersson CD, Almqvist F, Elofsson M, Enqvist P-A, Gustavsson A-L, Remez N, Mestres J, Marcou G, Varnek A; Hibert M, Quintana J, Frank R. Design of a General-Purpose European compound screening library for EU-OPENSCREEN. ChemMedChem 2014;9:2309–26.

## P31 RADAR: a research data repository for the “long-tail of science”

### Filipe Furtado^1^, Karina van den Broek^2^, Ludger Wessjohann^1^

#### ^1^Department of Bioorganic Chemistry, Leibniz-Institute of Plant Biochemistry Weinberg 3, D-06120 Halle (Saale); ^2^Chemistry Department, Ludwig-Maximilians-Universität München, Butenandtstr. 7, 81377 Munich

##### **Correspondence:** Ludger Wessjohann - Ludger.Wessjohann@ipb-halle.de

*Journal of Cheminformatics* 2016, **8(Suppl 1)**:P31

The ever growing production of digital data as basis for scientific research has led funding agencies to regard data preservation and reuse as transparency-enhancing and cost-effective policies. On the other hand, scientific communities have early recognized the advantage in the up-scaling and reuse of scientific data, and some subject-specific repositories have emerged within the respective research fields, while interdisciplinary ones [1–3] still remain scarce.

RADAR (Research Data Repository) [4,5] aims to provide a significant contribution to the above described scenario, and to establish itself as discipline-agnostic, sustainable, and cost-transparent infrastructure, guaranteeing long-term digital preservation and thereby enhancing the reuse of research data. An additional and key aim of the project is to serve as a platform to support the peer-review of scientific articles, in which the underlying primary data is jointly submitted with the written manuscript.

Specifically, our project offers two distinct services: I) a preservation service, in which datasets are digitally preserved, and kept externally inaccessible, and II) a publication service, in which datasets receive a DOI and are accessible, free of costs, through our site [4,5]. The implementation of an OAI-PMH standard and an open API, for the harvesting of metadata and (published) datasets, respectively, is under development.

RADAR is a DFG-funded (2013–2016) initiative, consisting of an interdisciplinary team formed by the FIZ Karlsruhe, TIB Hannover, KIT/SCC, LMU Munich and the IPB Halle. We actively seek cooperation and feedback, not only from publishers and manuscript submission systems, but especially from the scientific community.

**References**

1. Dryad: http://datadryad.org/. Accessed 04 Aug 2015.

2. Zenodo: http://www.zenodo.org/. Accessed 04 Aug 2015).

3. figshare: http://www.figshare.com/. Accessed 04 Aug 2015.

4. RADAR Project: http://www.radar-projekt.org/. Accessed 04 Aug 2015.

5. RADAR Test system: http://dev.fiz-karlsruhe.de/radar/de/index. Accessed 04 Aug 2015.

## P32 Class probability estimates for defining an applicability domain

### Miriam Mathea, Knut Baumann

#### Institute of Medicinal and Pharmaceutical Chemistry, Braunschweig University of Technology, Braunschweig, Germany

##### **Correspondence:** Miriam Mathea - m.mathea@tu-bs.de

*Journal of Cheminformatics* 2016, **8(Suppl 1)**:P32

The performance of chemoinformatic classification models is usually characterised by the prediction error (PE), which is estimated with an independent, external test data set. While the PE of the independent test set quantifies the overall model performance, rating the reliability of an individual prediction is of importance in decision making. To this end class probability estimates can be used. Generally, many supervised learning algorithms output some sort of probability estimate, but they need not be well calibrated. However, when assessing the reliability of a prediction well calibrated probabilities are definitely needed. Logistic regression is most commonly applied for calibrating class probability estimates and was originally used to calibrate support vector machine (SVM) outputs [1, 2].

In this study, logistic regression was employed to calibrate the outputs of six classification techniques (random forest (RF), k-nearest neighbour (KNN), SVM, partial least squares discriminant analysis (PLSDA), linear discriminant analysis (LDA) and naïve Bayes classifier (NBC). In addition to that, six regression techniques (random forest regression (RFR), support vector regression (SVR), sparse partial least squares (SPLS), lasso, elastic net and ridge regression) were also used to estimate class probabilities. The quality of the calibration is assessed with reliability diagrams [3]. In detail, the mean squared error of prediction (MSE) for predicted and actual class membership probabilities are computed to quantify the degree of improvement. Furthermore, the influence of accuracy, correlation structure of the predictor matrix, and dataset size on the quality of class probability estimation was analysed in several simulation studies as well as with real data sets. From these studies, the following conclusions can be drawn: 1) within the accuracy range of 0.70 to 0.95, the probability estimates are reliable; 2) accuracy and correlation have the largest influence on the quality of class probability estimation; 3) The data set size has minor influence.

With calibrated class probability estimates, it is straightforward to define the applicability domain by setting a threshold for the class membership probability below which the prediction of an object is rejected. Calibration is advantageous in this context to assure that the threshold coincides with the nominal probability.

**References**

1. Platt JC. Probabilistic outputs for support vector machines and comparisons to regularized likelihood methods. Adv Large Margin Class. 1999;10:61–74.

2. Niculescu-Mizil AJ. Predicting good probabilities with supervised learning. In Proceedings of the 22nd international conference of machine learning. 2005; Saso Dzeroski, editors. p. 625–632; 2005.

3. DeGroot MH. The comparison and evaluation of forecasters. Statistician 1982;32:12–22.

## P33 Global mapping of Traditional Chinese Medicine (TCM) into bioactivity space and pathways annotation improve mechanistic understanding and discovers relationships between therapeutic action (sub-)classes

### Siti Zuraidah Mohamad-Zobir^1^, Xianjun Fu^2^, Tai-Ping Fan^3^, Andreas Bender^1^

#### ^1^Centre for Molecular Informatics, Department of Chemistry, University of Cambridge, Lensfield Road, CB2 1EW, United Kingdom; ^2^School of Information Management, Shandong University of Traditional Chinese Medicine, 250355 Jinan, China; ^3^Department of Pharmacology, University of Cambridge, Tennis Court Road, Cambridge CB2 1PD, United Kingdom

##### **Correspondence:** Andreas Bender - ab454@cam.ac.uk

*Journal of Cheminformatics* 2016, **8(Suppl 1)**:P33

While Traditional Chinese Medicine (TCM) has been of great importance consistently throughout the years in China, there is still some more scientific rationale needs to be proven for it to be accepted further in the West. Given the increasing data of TCM chemical ingredients and the availability of large-scale bioactivity data, we are now in the position to provide computational hypotheses for the mode-of-actions (MOAs) of some TCM treatments, and to discover the relationship between the treatments. By using in silico target prediction algorithms, we generated a hierarchical clustering of 45 TCM therapeutic action (sub-)classes based on predicted bioactivity spaces, which were further annotated with KEGG pathways. The in silico target prediction of 10,079 TCM compounds showed a diverse bioactivity space, predicting a total of 409 unique targets and 171 unique pathways and 183 enriched targets and 99 enriched pathways based on Estimation Score ≤ 0 and ≥5% of compounds/targets in a (sub)-class. The TCM (sub-)classes were compiled into 14 clusters and the MOAs of each (sub)-class was established by associating the top three enriched targets/pathways to its indications with supporting literature. Overall, the most frequent top three enriched targets were immune-related targets such as tyrosine-protein phosphatase non-receptor type 2 (PTPN2) and protein-kinase C family while the most frequent enriched pathway was related to digestive system such as mineral absorption and bile secretion. For instance, compounds from “Hemostatic, stasis resolving” showed wound healing activity, which can be suggested from the action of compounds against PTPN2 [1,2] and the mineral absorption pathway [3]. The annotation of the protein family showed that the G-protein coupled receptor (GPCR) and protein kinase family were mainly contributed to the diversity of the bioactivity space and pathway mapping indicated that the digestive system was consistently annotated, which agreed with the important treatment’s thought of TCM, “the foundation of acquired constitution” that includes spleen and stomach. This global overview enables the observation of similarities and the differences between (sub-)classes, which are not apparent from the names given. Hence, this analysis hopefully helps to bridge the gap between TCM and Western medicine a bit further.

**References**

1. Doody KM, et al.. Immunol Rev. 2009;228:325–41.

2. Karodi R, et al. Int J App Res Nat Prod. 2009;2:12–8.

3. Lim Y, et al. J Nutr. 2004;134:811–6.

## P34 Optimising MIP inhibitors via docking and molecular dynamics simulations

### Maximilian A. Kuhn, Christoph A. Sotriffer

#### Institute of Pharmacy and Food Chemistry, University of Würzburg, Würzburg, 97074, Germany

##### **Correspondence:** Maximilian A. Kuhn - maximilian.kuhn@uni-wuerzburg.de

*Journal of Cheminformatics* 2016, **8(Suppl 1)**:P34

Bacterial Macrophage Infectivity Potentiator (MIP) proteins and human FK506-binding proteins (FKBPs) both belong to the class of peptidyl-prolyl-isomerases and exhibit a highly conserved binding pocket. The MIP protein of *Burkholderia pseudomallei* is important for replication and full virulence, rendering it a potential target for antimicrobial substances. Human FKBPs play miscellaneous roles, as for example in the regulation of steroid hormone receptor function and the immune response.

Several small-molecule pipecolic acid derivatives are capable of binding to MIP of *B. pseudomallei* [1], human FKBP and other MIP proteins, for example from *Legionella pneumophila* [2]. Analysing and comparing the structure-activity relationships for these complexes is crucial for the development of new MIP inhibitors with sufficient selectivity.

In this study, differences in binding affinity between these proteins were investigated using docking studies and molecular dynamics simulations. These analyses and the subsequent optimisation of the ligands are challenging owing to the narrow affinity range of the inhibitors and the shallow binding sites. A docking protocol was set up and validated by redocking to 24 crystal structures of FKBPs and MIP proteins of different organisms. Several directions for *in-silico* design of molecules exhibiting augmented selectivity are shown. Furthermore, a scoring-function-based binomial logistic regression model for prediction of *B. pseudomallei* MIP inhibitors with submicromolar affinity is presented.

**References**

1. Begley D, Fox III D, Jenner D, Juli C, Pierce P, Abendroth J, Muruthi M, Safford K, Anderson V, Atkins K, Barnes S, Moen S, Raymond A, Stacy R, Myler P, Staker B, Harmer N, Norville I, Holzgrabe U, Sarkar-Tyson M, Edwards T, Lorimer D. A structural biology approach enables the development of antimicrobials targeting bacterial immunophilins. Antimicrob Agents Chemother. 2014;58:1458–67.

2. Juli C, Sippel M, Jäger J, Thiele A, Weiwad M, Schweimer K, Rösch P, Steinert M, Sotriffer C, Holzgrabe U. Pipecolic acid derivatives as small-molecule inhibitors of the legionella MIP protein. J Med Chem. 2011;54:277–283.

## P35 Common mechanisms-of-action and molecular targets identified by pairwise associations of compounds based upon BioMAP cellular readouts

### Azedine Zoufir^1^, Xitong Li^2^, Lewis Mervin^1^, Ellen Berg^2^, Mark Polokoff^2^, Andreas Bender^1^

#### ^1^Unilever Centre for Molecular Informatics, Department of Chemistry, Lensfield Road, CB2 1EW Cambridge, UK; ^2^BioSeek, Inc., 310 Utah 100, South San Francisco, CA 94080, USA

##### **Correspondence:** Azedine Zoufir - az338@cam.ac.uk

*Journal of Cheminformatics* 2016, **8(Suppl 1)**:P35

The BioMAP systems are high-quality phenotypic screening assays and part of EPA’s ToxCast effort to prioritise chemical testing. Based on a robust metric, the nScore, 370 compounds were grouped into 82 clusters. These clusters are the results of common underlying mode-of-action and 4 out of the 82 clusters were identified with specific molecular targets using a novel target prediction algorithm. Indeed, only these four clusters were composed of compounds which all had at least one shared target in common. Moreover, these four clusters are equally identified when using two different metrics as activity cutoff, further confirming their selective mode-of-action as compared to other clusters. Different activities were found for these four clusters. The first and third clusters seemed to be associated with targets involved in nuclear receptor transcription, and are composed of compounds associated with cardiovascular toxicity biomarkers. However the third cluster is composed of compounds targeting proteins belonging to the family of retinoic acid receptors which additionally target lipid metabolism and adipocyte differentiation, while the first cluster is slightly more diverse and have compounds associated with fibrosis and restenosis biomarkers in addition of the cardiovascular inflammation biomarkers. The second cluster seemed to be associated with one specific target, the Sphingosine 1-phosphate receptor 2, involved in G alpha signalling events and lysosphingolipid activity, and compounds belonging to this cluster do not seem to be associated with specific biomarkers. The last cluster is associated with 7 targets active mostly in steroid hormone bio-synthesis, and with cardiovascular and fibrosis biomarkers. The utility of this approach lies in the fact that compounds having the same target profile may be associated with one of these clusters to better understand their mode-of-action. As an example, 16 compounds were associated with one of the 4 clusters, based on their predicted targets. For 3 of the 4 clusters, the biomarker profiles of the newly associated compounds were similar to those already in the cluster confirming the relevance of this approach. This approach may enable a comprehensive understanding of compound in terms of mechanism-of-action, associated pathways and phenotypic profile.

## P36 Will that nitrogen please stop jumping around!?!

### Wolf D. Ihlenfeldt

#### Xemistry GmbH, Königstein, D-61462, Germany

##### **Correspondence:** Wolf D. Ihlenfeldt - wdi@xemistry.com

*Journal of Cheminformatics* 2016, **8(Suppl 1)**:P36

Generating quality 2D layout coordinates of structures from connection tables is a surprisingly difficult problem. Even if the general connectivity of a compound is mapped to a visually pleasing layout, this is not sufficient to result in a compound rendering which is immediately recognizable to the eye of the pattern-trained chemist. There are many implicit conventions in the layout of chemical structures, concerning standard orientations of ring systems as well as the placement of hetero atom within this ring and substituents on it or the selection of bonds with wedges to indicate stereochemistry. Textbooks generally agree on the layout of basic compounds, while most 2D chemical layout software has no notion about these. The consequence is, for example, that one can find in online databases drawings of pyridine (a trivial layout case where a simple polygon ring drawing algorithm is sufficient) with the nitrogen in all six possible positions, while we were unable to find any textbook where it was not on the bottom.

The Cactvs toolkit is the layout and rendering engine behind the PubChem database. Since more than a decade, the software has been one of the few engines which have been using advanced layout rules implementing implicit conventions in addition to computing a reasonable 2D graph. This rule set has been significantly expanded, and includes improvements implemented in response to feedback of PubChem users and developers, as well as 3rd party vendors which have licensed the engine. We discuss details and examples of these developments.

## P37 The Cactvs KNIME node compiler

### Wolf D. Ihlenfeldt

#### Xemistry GmbH, Königstein, D-61462, Germany

##### **Correspondence:** Wolf D. Ihlenfeldt - wdi@xemistry.com

*Journal of Cheminformatics* 2016, **8(Suppl 1)**:P37

Within a few short years, the KNIME [1] dataflow environment has become an indispensable tool in chemical data analysis. The basic software already ships with a reasonable collection of chemistry nodes. It also enjoys the support of many vendors which provide a rich set if additional nodes covering most standard data processing needs for typical chemistry studies.

However, it frequently happens that there are minor gaps in the capabilities of the turnkey nodes. The development of custom nodes within KNIME is possible, but requires an intimate knowledge of its software architecture and Java. A tool which would allow the specification of custom chemistry-aware nodes on a high level, for example by encapsulating scripts of a chemical information processing toolkit, would be a welcome addition. While scripting nodes exist in the KNIME collection, they either lack chemistry awareness, or are very limited in which actions they can perform.

We have now developed a KNIME node compiler for the Cactvs Cheminformatics Toolkit as a new toolkit module. It directly generates a complete KNIME plugin-in Jar file from a very terse and high-level node description and a custom script. This script can perform any operation the toolkit in its stand-alone form is capable of. The new feature is a significant addition to the capabilities of the toolkit. Examples are shown and the unique development process for these custom nodes outside of the KNIME software proper is explained.

**Reference**

1. Berthold MR, et al. KNIME: The {K}onstanz {I}nformation {M}iner. studies in classification, data analysis, and knowledge organization (GfKL 2007). Berlin: Springer; 2007.

## P38 Virtual screening and biochemical testing of new *M. tuberculosis* thioredoxin reductase inhibitors

### Jette Pretzel, Oliver Koch

#### Department of Chemistry and Chemical Biology, TU Dortmund University, Otto-Hahn-Str. 6, 44227 Dortmund, Germany

##### **Correspondence:** Jette Pretzel - jette.pretzel@tu-dortmund.de

*Journal of Cheminformatics* 2016, **8(Suppl 1)**:P38

Tuberculosis, caused by *Mycobacterium tuberculosis*, is one of the world’s deadliest infectious diseases affecting nine million people worldwide [1]. Due to emerging resistance against currently available antituberculotic drugs [2], novel potent inhibitors are urgently needed. *M. tuberculosis* thioredoxin reductase (*Mt*TrxR) present a promising drug target since it plays a crucial role in antioxidative defense, proliferation, and growth of *M. tuberculosis*, based on thioredoxin-dependent reduction of target proteins e.g. ribonucleotide reductase with downstream effects on DNA synthesis [3, 4].

In a previous high-throughput docking approach, promising small molecule inhibitors of *Mt*TrxR were identified that target the thioredoxin binding site [5]. In order to improve inhibition activity, extend the structure-activity relationship, and validate selected initial hits as real hits, we performed virtual screening campaigns for these scaffold classes.

We will present the results of this *in-silico* analysis and the biochemical testing.

**References**

1. WHO: Global Tuberculosis Report 2014.

2. Aziz M, Wright A, et al. Epidemiology of antituberculotic drug resistance. Lancet 2006;368:2142–54.

3. Master S, Springer B, et al. Oxidative stress response genes in Mycobacterium tuberculosis. Microbiol. 2002;148:3139–44.

4. Arner E, Holmgren A: Physiological functions of thioredoxin and thioredoxin reductase. FEBS J. 2002;267:6102–09.

5. Koch O, Jaeger T, et al. Identification of M. tuberculosis thioredoxin reductase inhibitors based on high-throughput docking using constrains. J Med Chem. 2013, 56:4849–59.

## P39 Homology modelling, molecular dynamics and virtual screening of the mitochondrial sirtuins SIRT4 and SIRT5

### Zayan Alhalabi, Wolfgang Sippl

#### Institute of Pharmacy, Martin-Luther University Halle-Wittenberg, Germany

##### **Correspondence:** Zayan Alhalabi - zayan.alhalabi@pharmazie.uni-halle.de

*Journal of Cheminformatics* 2016, **8(Suppl 1)**:P39

The histone deaceylases SIRT4, and SIRT5 are mitochondrial proteins and are considered as metabolic sensors of cell’s energetic status. The two enzymes have important role in several human diseases such as cancer, and diabetes. SIRT4 regulates the glutamate dehydrogenase activity and insulin secretion, SIRT4 also functions as a cellular lipoamidase that regulates the pyruvate dehydrogenase (PDH), Its catalytic efficiency for lipoyl and biotinyl lysine modifications is superior to its deacetylation activity [1–3] whereas SIRT5 removes post-translational modifications such as lysine malonylation and succinylation [4]. Recent studies reported that SIRT4 seems to have a tumor-suppressive function [5,6] and may serve as a novel therapeutic target in colorectal cancer [7]. SIRT5 regulates urea production, reactive oxygen species (ROS) metabolism, via regulating the carbamoyl phosphate synthetase (CPS1) [3].

Due to the absence of a crystal structure for human SIRT4, a homology model was generated using different templates and computational approaches. The template selection identified human SIRT5 as most suitable template. MD simulations were carried out on the SIRT4 models in complex with the cofactor and different substrates using program AMBER 12 to understand the stability and conformational changes of the modeled proteins in holo and apo form. In addition, several MD simulations of available crystal structures of SIRT5 in complex and in apo form have been performed and compared with the results obtained for SIRT4. In addition, shape-based virtual screening for inhibitors and activators have been carried out.

**References**

1. Mathias RA, Rowland EA, Greco TM, et al. Sirtuin 4 is a lipoamidase regulating pyruvate dehydrogenase complex activity. Cell 2014;159:1615–25.

2. Shih J, Donmez G: Mitochondrial sirtuins as therapeutic targets for age-related disorders. Genes & Cancer 2013;4:91–6.

3. Parihar P, Solanki I, et al. Mitochondrial sirtuins: Emerging roles in metabolic regulations, energy homeostasis and diseases. Exp Gerontol. 2015;61:130–41.

4. He W, Newman J, Wang M, Ho L, Verdin E. Mitochodrial sirtuins: regulators of protein acylation and metabolism. Trends Endocrin Metabol. 2012;23:467–76.

5. Vatrinet R, Iommarini L, et al. Targeting respiratory complex I to prevent the Warburg effect. Int J Biochem Cell Biol. 2015;63:41–5.

6. Jeong SM, Xiao C, et al. SIRT4 has tumor-suppressive activity and regulates the cellular metabolic response to DNA damage by inhibiting mitochondrial glutamate metabolism. Cancer Cell 2013;23:450–63.

7. Miyo M, Yamamoto H, Konno M, et al. Tumour-suppressive function of SIRT4 in human colorectal cancer. Br J Cancer. 2015.

## P40 QSAR modeling independent of input tautomers

### Robert Fraczkiewicz, Marvin Waldman, Robert D. Clark

#### Simulations Plus, Inc., Lancaster, CA 93534, USA

##### **Correspondence:** Robert Fraczkiewicz - robert@simulations-plus.com

*Journal of Cheminformatics* 2016, **8(Suppl 1)**:P40

The quality and predictivity of most QSAR models used in drug design and development often depend on the particular tautomeric and valence structures used to represent the molecules of interest. This is because the location of hydrogens, bond orders, and formal charges affect the values of atomic and molecular descriptors upon which the models are based. For descriptors such as partial charges, this dependence is typically due to basing atom typing on hybridization: e.g., sp^3^-hybridized oxygen in -OH is treated differently than its sp^2^-hybridized counterpart in the =O group. That same oxygen may well occur in both forms in different tautomers of the same molecule. In the aqueous environment of interest in drug design applications, representing a compound as any one of its tautomers is likely to distort QSAR models trained using that tautomer. Even worse is the danger of choosing a tautomer present at low abundance, which will bias the model-building process or the reliability of predictions or both. We have developed a descriptor generation method which is independent of tautomer and valence structure representation to address this problem and will illustrate its application to the atomic descriptors used in S+pKa, which is our global model of protic ionization constants. Preliminary results will be shown and compared with the “traditional” approach along with a discussion of advantages and potential pitfalls of the method. For example, “traditional” pKa predictions for amitrole produced five different sets of results depending on input tautomer (10.73, 3.56; 11.16, 4.84; 12.84, 9.94, 5.53; 11.31, 4.37; 14.79, 11.42, 7.47) whereas the new method yielded unique results (9.41, 4.02) regardless of the input tautomer.

## P41 A proteochemometrics based approach for therapeutic target prediction

### Neem Shaikh, Prabha Garg

#### Department of Pharmacoinformatics, National Institute of Pharmaceutical Education and Research (NIPER), Sector-67, S.A.S. Nagar, Punjab 160 062, India

##### **Correspondence:** Prabha Garg - prabhagarg@niper.ac.in

*Journal of Cheminformatics* 2016, **8(Suppl 1)**:P41

Despite recent advancements in biological and medicinal chemistry research, the numbers of new approved drugs are decreasing. There are more than 3000 potential drug targets surfaced as an outcome of Genomics revolution [1] and nearly 1033 compounds are estimated as drug-like chemical space [2]. Profiling interactions of these entities are much needed to purpose or repurpose chemical substances towards therapeutic targets. Since experimental ways to profile these chemical substances are costly, time-consuming and impossible on such large scale, computational prediction becomes an impending complement that offers valuable information in an efficient way. Amongst many available approaches, proteochemometrics (PCM) is distinctly beneficial in exploiting ever-increasing drug-target interaction data [3]. PCM is an extrapolation of classical Quantitative structure–activity relationship (QSAR), which were trained only on chemical data for particular target. On the other hand, PCM models are trained on both biological and chemical data for multiple targets by various machine learning techniques.

**Fig. 5:**
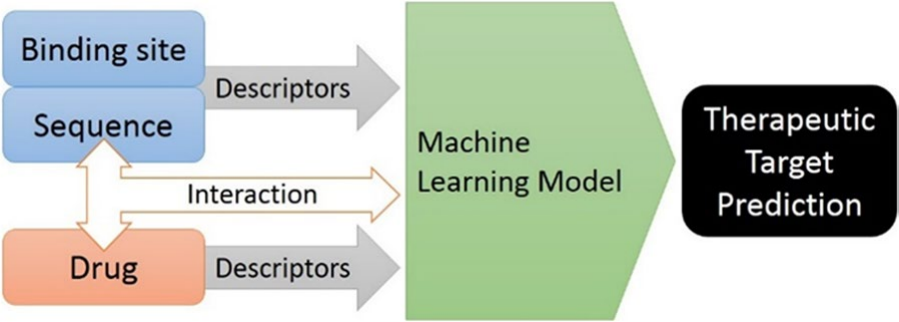
Schematic overview of the proteochemometrics based approach

In this work, a systematic approach based on PCM is reported (Fig. 5), which can predict interaction profile of given chemical moiety against therapeutic targets. Total 3036 interactions of 1473 proteins extracted from sc-PDB database are used for training the models [4]. This study also compares set of machine-learning algorithms (Random Forest and Support Vector Machine) and protein descriptors (Structure-based and Sequence-based) for the proposed task. Best results were obtained by Random Forest model trained on Structure-based protein descriptors with the accuracy of 99% and 86% for test and external validation set respectively. Outcome of the study suggest that proposed models could be used to not only elucidate the intricate mechanism of action of small molecules by identifying novel therapeutic target but also provide an opportunity to reposition existing drugs for new therapeutic application.

**References**

1. Russ AP, Lampel S. The druggable genome: an update. Drug Discov Today 2005;10:1607–10.

2. Polishchuk P, Madzhidov T, Varnek A: Estimation of the size of drug-like chemical space based on GDB-17 data. J Comput Aided Mol Des. 2013;27:675–9.

3. van Westen GJ, Wegner JK, IJzerman AP, van Vlijmen HM, Bender A. Proteochemometric modeling as a tool to design selective compounds and for extrapolating to novel targets. MedChemComm. 2011;2:16–30.

4. Meslamani J, Rognan D, Kellenberger E. sc-PDB: a database for identifying variations and multiplicity of ‘druggable’ binding sites in proteins. Bioinformatics 2011;27:1324–6.

## P42 iScienceSearch: the Internet search engine for chemists & biologists

### Alexander Kos, Hans-Jürgen Himmler

#### AKos GmbH, Steinen, Germany

##### **Correspondence:** Alexander Kos - software@akosgmbh.de

*Journal of Cheminformatics* 2016, **8(Suppl 1)**:P42

iScienceSearch (http://isciencesearch.com/iss) is a free of charge application to search over 100 databases on the Internet, by structure, substructure, similarity, text and synonyms. If you type a name, we generate in the background automatically the chemical structure, the CAS Registry Number, other identifiers like AKOS number, and different names, if possible. We call this extended search. This means if you type Tamiflu, you also get answers that are perfectly correct, and where only the word Oseltamivir appears. Oseltamivir is a synonym for Tamiflu. You cannot search Google by chemical structure, and SciFinder is not an Internet search engine. iScienceSearch is the system of choice searching the Internet for chemists and anybody who needs chemical information.

## P43 Chemical Registration and Publishing System (ChemRPS) with Windows client

### Alexander Kos, Hans-Jürgen Himmler

#### AKos GmbH, Steinen, Germany

##### **Correspondence:** Alexander Kos

*Journal of Cheminformatics* 2016, **8(Suppl 1)**:P43

Small chemical companies or suppliers of chemicals you would like to have a chemical information systems. However, you need to overcome a number of problems.

- You have limited know-how about chemoinformatics
- Systems are expensive, especially if you want to equip everybody in your company
- Systems are difficult to maintain

**Fig. 6:**
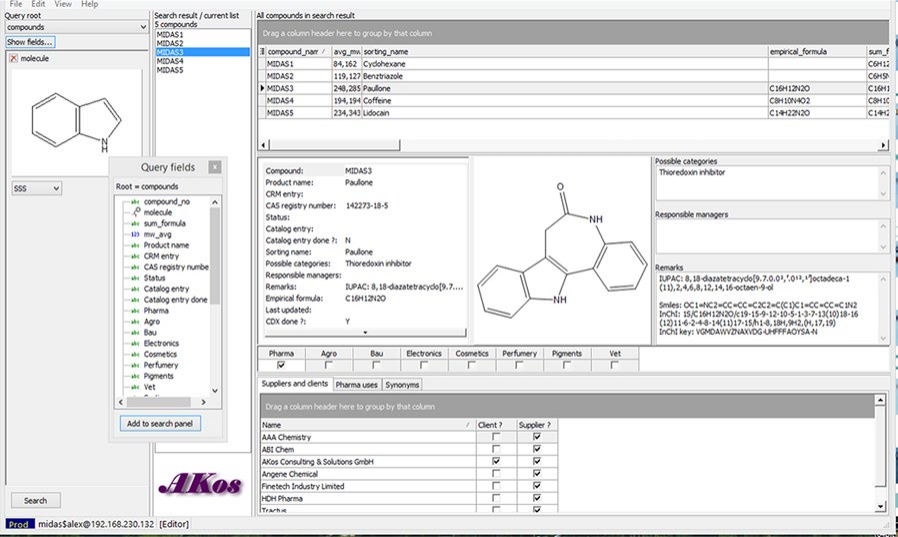
Screenshot of the Windows client

We offer you a solution that is based on Open Access software (Fig. 6).

## P44 Different readout mechanisms for protein-DNA interactions investigated with MD simulations

### Achim Sandmann, Christophe Jardin, Heinrich Sticht

#### Bioinformatics, Institute for Biochemistry, FAU Erlangen-Nürnberg, Fahrstr. 17, Erlangen, 91054 Germany

##### **Correspondence:** Achim Sandmann - Achim.Sandmann@fau.de

*Journal of Cheminformatics* 2016, **8(Suppl 1)**:P44

The global regulator Carbon catabolite Protein A (CcpA) controls carbon metabolism in Bacillus subtilis. It does so by binding to the degenerate consensus site WTGNNARCGNWWWCAW [1]. To investigate how CcpA can bind to such diverse sequence motifs, we tried to identify contributions to binding selectivity. On the one hand, direct contacts via hydrogen bonds and nonpolar interactions to the nucleotide bases (‘base readout’) strongly influence the preference of proteins for specific sequences. On the other hand, the base composition also influences the shape and flexibility of the DNA, thereby modulating the strength of the interactions.

The strongest conserved bases of the consensus site are the central CG bases, at which the DNA is bent in the complex structure. The direct contacts of the bases cannot explain the selectivity in respect to central GC bases. In addition, CG represents a pyrimidine-purine base step, which is known to facilitate kinks, which might favour shape readout at this site.

To dissect the individual contributions of base and shape readout, the CcpA-DNA complex was simulated as wild type and compared to a mutant, in which the central CG base step was replaced by GC. The CcpA-DNA hydrogen bonds remained stable throughout the simulation of the mutant, whereas the comparison of different MMPBSA analysis protocols showed a higher energy requirement to bend the mutant DNA sequence.

**Reference**

1. Schumacher MA, Sprehe M, Bartholomae M, Hillen W, Brennan RG. Structures of carbon catabolite protein A-(HPr-Ser46-P) bound to diverse catabolite response element sites reveal the basis for high-affinity binding to degenerate DNA operators. Nucleic Acids Res. 2011;39:2931–42.

## P45 Free energy calculations in fragment based drug design: Applying FEP in practical ligand optimization

### Thomas B. Steinbrecher^1^, Markus Dahlgren^2^, Daniel Cappel^1^, Teng Lin^2^, Lingle Wang^2^, Goran Krilov^2^, Robert Abel^2^, Richard Friesner^3^, Woody Sherman^2^

#### ^1^Schrödinger GmbH, Dynamostr. 13, 68165 Mannheim, Germany; ^2^Schrödinger Inc., 120 West 45th Street, 17th Floor, New York, NY, 10036, USA; ^3^Department of Chemistry, Columbia University, 3000 Broadway New York, NY, 10027, USA

##### **Correspondence:** Thomas B. Steinbrecher - thomas.steinbrecher@schrodinger.com

*Journal of Cheminformatics* 2016, **8(Suppl 1)**:P45

It has long been the holy grail of computational structure-based ligand design to accurately predict binding free energies for novel compounds. This task is of special importance in fragment-based drug design, where typically multiple rounds of potency improvement are necessary to generate highly active lead structures out of micromolar initial inhibitors.

Molecular Dynamics based free energy calculations (or FEP for free energy perturbation) are among the most suitable methods to reach this goal, which would significantly impact the modern drug design process. Many of the issues previously encountered with FEP have been mitigated by our introduction of the FEP+ (free energy perturbation plus REST, i.e. replica-exchange with solute tempering) methodology along with state-of-the-art modern force field, together with the computational power offered by GPU computing.

The lack of large scale validation studies on diverse series of ligands are another obstacle for the practical application of FEP, due to the lack of computational resources and the time consuming process of simulation setup and analysis. Recently, we have conducted a validation study of FEP results on more than 10 targets and more than 500 compounds, offering an order of magnitude more data than typical FEP studies and allowing statistically valid conclusion about their efficacy.

Here, we extend this validation study to fragment hit series binding a variety of pharmaceutically relevant target systems. Results for seven diverse protein systems with about 100 ligands are presented. Relative binding free energies can be calculated with good accuracy, typically with R-squared values in the range of 0.5–0.8 and mean unsigned errors (MUE) of about 1 kcal/mol when comparing to experimental data. FEP+ significantly outperforms alternative methods to determine binding free energies. This suggests that FEP+ binding energy predictions offer unsurpassed accuracy for fragments and lead precursors as well as for drug-like molecules and could become a regular part of FBDD projects.

## P46 What determines docking performance in drug discovery? A case study of PTR1, an anti-parasitic target

### Ina A. Pöhner^1^, Joanna Panecka^1^, Rebecca C. Wade^1,2^

#### ^1^Molecular and Cellular Modeling Group, Heidelberg Institute for Theoretical Studies (HITS) gGmbH, Heidelberg, Germany; ^2^ZMBH-DKFZ Alliance, Center for Molecular Biology, Heidelberg University, Heidelberg, Germany

##### **Correspondence:** Ina A. Pöhner - ina.poehner@h-its.org

*Journal of Cheminformatics* 2016, **8(Suppl 1)**:P46

The target-based identification and optimization of new drug leads heavily relies on computational techniques. Among these, virtual docking is a common method for investigation and evaluation of interactions between small molecules and their receptors. Crucial questions for any docking study are: How well do the docking results agree with experimental data? Do the observed complexes occur in reality and is there a relationship between the docking score and the binding affinity of a compound? It is common knowledge that the performance of docking procedures is generally target dependent. However, there may be many other factors that limit the predictive capabilities of docking.

In our research, we utilize docking for drug discovery against neglected tropical diseases like Human African Trypanosomiasis and leishmaniasis, which present a threat to millions of people world wide, who suffer from the limited number of currently available treatment options. One enzyme involved in the parasitic folate metabolism, pteridine reductase 1 (PTR1) is unique to the parasites, which makes it a promising target for anti-parasitic compound design.

We investigated different sets of chemically diverse PTR1 inhibitors with a small set of receptor template structures, but varying other aspects like docking methodology, protonation states and presence of structural waters. Our results show some key aspects for the choice of the method and pinpoint pitfalls in the selection of a receptor template. As such effects are likely to impact other targets as well, the study should aid future target-based drug design efforts by highlighting important considerations beyond the selection of a receptor template for docking studies.

**Acknowledgement:** We gratefully acknowledge the EU (FP7 NMTrypI project grant agreement No. 603240) for financial support and members of NMTrypI for providing experimental data.

## P47 SMARTSminer: Interactively mining discriminative patterns

### Stefan Bietz, Karen T. Schomburg, Matthias Hilbig, Matthias Rarey

#### Center for Bioinformatics, University of Hamburg, Hamburg, Germany

##### **Correspondence:** Matthias Rarey - rarey@zbh.uni-hamburg.de

*Journal of Cheminformatics* 2016, **8(Suppl 1)**:P47

Discriminative chemical patterns are used to distinguish molecules with different effects. Classifying the inhibiting, activating or toxicological potential of an unknown molecule constitutes a central aspect in cheminformatics. The structural features of a molecule responsible for a certain effect can be represented with a chemical pattern. A chemical pattern is a substructure of the molecule, generalized or specialized with molecular features and logic operators. The SMARTS language [1] is the most frequently used representation for chemical patterns. While SMARTS strings constitute a powerful concept for the representation and processing of abstract chemical patterns, their manual generation remains a complex and time-consuming process.

Here, we introduce SMARTSminer [2], a new algorithm for the automatic derivation of discriminative SMARTS patterns from preclassified molecule sets. Based on a specially adapted subgraph mining algorithm, SMARTSminer identifies structural features that are frequent in only one of the given molecule classes. In comparison to elemental substructures, it also supports the consideration of general and specific SMARTS features. Furthermore, SMARTSminer is integrated into an interactive pattern editor named SMARTSeditor [3]. This allows for an intuitive visualization on the basis of the SMARTSviewer [4] concept as well as interactive adaption and further improvement of the generated patterns. Additionally, a new molecular matching feature provides an immediate feedback on a pattern’s matching behavior across the molecule sets. SMARTSminer’s functionality and its integration into the SMARTSeditor software is shown in different classification scenarios.

**References**

1. James CA, Weininger D: Daylight theory manual, version 4.9; Laguna Niguel, CA: Daylight Chemical Information Systems, Inc.;2011.

2. Bietz S, Schomburg KT, Hilbig M, Rarey M: Discriminative chemical patterns: automatic and interactive design. J Chem Inf Model. 2015;55:1535–46.

3. Schomburg KT, Wetzer L, Rarey M. Interactive design of generic chemical patterns. Drug Discov Today 2013;18:651–8.

4. Schomburg K, Ehrlich HC, Stierand K, Rarey M. From structure diagrams to visual chemical patterns. J Chem Inf Model. 2010;50:1529–35.

## P48 The use of force field and quantum chemistry based methods to overcome the lack of structural information in PDB structures with very low resolution

Christian Jäger^1^, Vivien Wieczorek^1^, Lance M. Westerhoff^2^, Oleg Y. Borbulevych^2^, Hans-Ulrich Demuth^1^, Mirko Buchholz^1^

### ^1^Fraunhofer Institute for Cell Therapy and Immunology, Department of Drug Design and Target Validation (IZI-MWT), 06120 Halle (Saale), Germany; ^2^QuantumBio Inc, 2790 West College Avenue, Suite 900, State College PA 16801, United States

#### **Correspondence:** Christian Jäger - christian.jaeger@izi.fraunhofer.de

*Journal of Cheminformatics* 2016, **8(Suppl 1)**:P48

For structure based approaches a reliable structural model of the target protein, ideally bound with one or more effectors, is an essential requirement.

Besides internal industrial databases, a primary resource for such models is represented by the World Wide Protein Data Bank (wwPDB) [1], or PDB for short. Its repositories contain more than 113000 biological macromolecular structures of various qualities (as of August 2015). Most of these structures referring to a resolution below 3 Å. This number is a critical threshold for an appropriate refinement and structural meaningful optimization, to achieve the best possible fit between observed experimental structure factors (e.g. electron densities) and the chosen target function.

However, more than 7000 structures listed in the PDB were deposited with a resolution worse than 3 Å. The use of these structural models is questionable for structure-based approaches. Such low resolution structures are also often lacking the information of essential interactions between the effector molecule and the target protein because of unresolved side chains.

For such purposes we hereby present an extended refinement strategy to increase the structural information content of a crystal structure model. As an example a crystal structure of the Vascular Endothelial Groth Factor Receptor 3 (VEGFR-3) with its ligand VEGF-C is used for further *in silico* modelling and binding studies. The resolution of this structure is 4.2 Å and lacks the information about crystallographic water molecules. Therefore a solvent analysis with 3D-RISM was applied to the complex. A combined standard crystallographic model-building of PHENIX and the structure modelling methodology of Rosetta was used, that utilizes an all-atom force field [2]. We will demonstrate the impact of a semi-empirical, linear-scaling, quantum-mechanic method on the PHENIX refinement process [3]. The combination of all 3 different approaches was optimized to a workflow that produces a reliable structural model, useful for a meaningful analysis of ligand–receptor interactions, not seen in the original model. We believe such a workflow could be routinely established and may be important for several drug discovery programs.

**References**

1. http://www.wwpdb.org/.

2. DiMaio F, Echols N, Headd JJ, Terwilliger TC, Adams PD, Baker D. Improved low-resolution crystallographic refinement with Phenix and Rosetta. Nat Methods. 2013;10:1102–4.

3. Borbulevych OY, Plumley JA, Martin RI, Merz Jr KM, Westerhoff LM: Accurate Macromolecular Crystallographic Refinement: Accurate macromolecular crystallographic refinement: incorporation of the linear scaling, semiempirical quantum-mechanics program DivCon into the PHENIX refinement package. Acta Cryst. 2014;D70:1233–47.

## P49 DUPED: A concept for decoy protein binding pockets

### Denis Schmidt^1^, Thomas Rickmeyer^1^, Timo Krotzky^1,2^, Peter Kolb^1^

#### ^1^Pharmaceutical Chemistry, Philipps-University, Marburg, Germany; ^2^The Cambridge Crystallographic Data Centre, Cambridge, UK

##### **Correspondence:** Denis Schmidt - denis.schmidt@uni.marburg.de

*Journal of Cheminformatics* 2016, **8(Suppl 1)**:P49

We present the complement of small molecule decoys on the side of protein binding pockets. The underlying concept of decoys is that they match the physico-chemical properties of their respective reference, while being structurally distinct [1]. Using Cavbase [2], a resource for abstract descriptions of protein binding pockets by so-called “pseudocenters“, protein decoy pockets are selected for any given reference binding site. We consider the types and number of pseudocenters as descriptor for the physico-chemical properties of a binding pocket. Accordingly, these descriptors should match between reference and decoy pocket. The recently developed RAPMAD approach [3] is used to select the most diverse pockets from those with matching physico-chemical properties to ensure structural dissimilarity. RAPMAD is a comparison method for Cavbase pockets, which is sufficiently fast for database-wide screening approaches.

Protein decoy pockets can be applied in contexts comparable to those for ligand decoys, i.e. to assess docking performance in inverse docking setups or to test new ligand decoy sets. Another particularly interesting application is the use of protein decoys in virtual screening to rescale docking scores. Docking scores corrected by these means can reduce the number of false positive hits as it has been described for ligand decoys [4].

Details on the decoy pocket selection method and results will be presented at the conference.

**References**

1. Mysinger MM, Carchia M, Irwin JJ, Shoichet BK. Directory of useful decoys, enhanced (DUD-E): better ligands and decoys for better benchmarking. J Med Chem. 2012;55:6582–94.

2. Schmitt S, Kuhn D, Klebe G. A new method to detect related function among proteins independent of sequence and fold homology. J Mol Biol. 2001;323:387–406.

3. Krotzky T, Grunwald C, Egerland U, Klebe G. Large-scale mining for similar protein binding pockets: with RAPMAD retrieval on the fly becomes real. J Chem Inf Model. 2015;55:165–79.

4. Wallach I, Jaitly N, Nguyen K, Schapira M, Lilien R: Normalizing molecular docking rankings using virtually generated decoys. J Chem Inf Model. 2011;51:1817–30.

## P50 Influence of molecular environment on protein interactions

### Sumit Mittal, Elsa Sánchez-García

#### Max-Planck-Institut für Kohlenforschung, Kaiser-Wilhelm-Platz 1, 45470 Mülheim an der Ruhr, Germany

##### **Correspondence:** Sumit Mittal - mittal@mpi-muelheim.mpg.de

*Journal of Cheminformatics* 2016, **8(Suppl 1)**:P50

Molecular environment of a biomolecule can affect its structure and function to different extents. We aim to study such influences for the following systems:

1. Human Islet amyloid polypeptide (hIAPP) is a 37-residue peptide which forms amyloid deposits associated with type-II diabetes mellitus. It is still unclear what triggers the conversion of soluble monomeric hIAPP into insoluble amyloid fibrils. We used replica exchange molecular dynamics (REMD) to sample the conformational space of the hIAPP dimer in explicit water, thus our work provides insights into the structural properties relevant to amyloid formation.

2. Huntington’s disease (HD) is associated with the expansion of polyglutamine (polyQ) stretch of the huntingtin (htt) protein. Above a threshold of 37 glutamines huntingtin exon 1 starts to aggregate in a nucleation dependent manner. A 17-residue N-terminal fragment of exon 1 (N17) was shown to play a crucial role in modulating aggregation propensity and toxicity of htt exon 1. We used molecular dynamics simulations to show that binding of CLR01 induces structural rearrangements within the N17 region of htt exon 1 monomer that leads to change in aggregation pathway of htt.

**References**

1. Luca S, Yau WM, Leapman R, Tycko R. Biochemistry 2007;46.

2. Acharya S, et al. J Biol Chem. 2014;289:10727.

## P51 Combination of multivariate analysis, chemosystematics, and QSRR study for identification and targeted isolation of natural products with anti-protozoal activity from Asteraceae

### Mauro S. Nogueira^1^, Tiago B. Oliveira^2^, Fernando B. da Costa^2^, Thomas J. Schmidt^1^

#### ^1^Institute of Pharmaceutical Biology and Phytochemistry, University of Muenster, Muenster, Correnstraße 48, 48149, Germany; ^2^University of São Paulo, Ribeirão Preto, Av. do Café S/N, Brazil

##### **Correspondence:** Mauro S. Nogueira - nogueira@uni-muenster.de

*Journal of Cheminformatics* 2016, **8(Suppl 1)**:P51

Sleeping Sickness, Leishmaniasis and Malaria, infections transmitted by protozoan parasites, are responsible for a great number of deaths and disease burden in tropical countries. New treatments are an urgent need. In this work combined correlative modelling (PLS) linking plant metabolite profiles and antiprotozoal activity with chemosystematic information, and *in silico* chromatographic retention time (*tR*) prediction to aid dereplication (the identification of known compounds) and the targeted isolation of known and new natural products with anti-protozoal activity. First, PLS correlative modeling (*R* 3.1.1 and *The Unscrambler* 9.2) was performed for UHPLC/ESI-QqTOF MS/MS data of extracts from Brazilian Asteraceae (n=140) and their *in vitro* activities against the protozoa *Trypanosoma brucei rhodesiense*, *Leishmania donovani*, and *Plasmodium falciparum* to select variables (metabolite data: *m/z*-*tR*) that were positively correlated with the activity. Secondly, the MS-data of these variables were related to the entries of an in house database containing the elemental formula and calc. *m/z* for 1360 natural products and taxonomic information on the plant species containing them. Hits for a particular elemental formula which were not reported as constituents of related plants (genus and tribus level) were excluded (chemosystematic filter). Then, 3D structures of the remaining hits were generated and 3D descriptors calculated (MOE 2011.10) and treated as previously reported [1]. An artificial neural network (ANN) model (Weka 3.6.6) as described in [1] was used to predict the hits’ retention time (*tRp*) in reverse phase HPLC-UV and to compare them with the experimental values (*tRe*). Selected compounds were isolated and structural elucidation was performed by means of MS and NMR. Several variables/compounds (n = 12) were positively correlated with the antiprotozoal activity. The initial number of possible structures related to an elemental formula (exact mass error: 20 ppm) queried in the database ranged from 5–70. Chemosystematic filtering reduced the number of possible structures to a range of 4–15. Comparison of *tRe* and *tRp* then greatly facilitated the identification of known compounds since only 1–2 possibilities remained. Seven of the compounds were isolated and all shown to be active against the protozoan parasites as suggested by the PLS correlation models. Three of them were new natural products. Overall, the combination of techniques proposed herein may complement MS-based dereplication, and can be successfully applied to streamline the discovery of new compounds with biological activity, such as an anti-protozoal potential, in crude extracts without time-consuming activity-guided fractionation.

**Reference**

1. Oliveira TB, Gobbo-Neto L, Da Costa FB, Schmidt, TJ. Study of chromatographic retention of natural terpenoids by chemoinformatic tools. J Chem Inf Model. 2014;55:26–38.

